# Supplementary material for: Clinical and cost-effectiveness of DREAMS START (Dementia RElAted Manual for Sleep; STrAtegies for RelaTives) for people living with dementia and their carers: a study protocol for a parallel multicentre randomised controlled trial
Source: BMJ Open. 2024 Feb 1;14(2):e075273. doi: 10.1136/bmjopen-2023-075273 (PMC10836385; doi:10.1136/bmjopen-2023-075273)
Supplement: Supplementary data [file bmjopen-2023-075273supp001.pdf]

Appendix 1 Informed consent forms

Site/Centre Number:

Dyad Number:

Participant Identification Number:

[INSERT NHS TRUST LOGO HERE]

FAMILY CARER CONSENT FORM

Title: **DREAMS START: Dementia-related manual for sleep strategies for relatives**

Name of Researcher: Please initial box

1. I confirm that I have read the information sheet dated 20-DEC-2020 (version 2.1) for the above study and understand the purpose of the research and I have received a copy of the information sheet and informed consent form to keep
2. I have had the opportunity to consider the information, ask questions and have had these answered satisfactorily
3. I understand that my participation is voluntary and that I am free to withdraw from the research study at any time without my legal rights or medical care being affected.
4. I understand that that my personal information will be processed only for the purposes of research and that all material and personal information will be kept in accordance with General Data Protection Regulation (GDPR) and the Data Protection Act 2018.
5. I understand that relevant data collected during the study may be looked at by individuals from the sponsor of the trial (Camden & Islington NHS Foundation Trust), UCL, regulatory authorities or from the NHS Trust, where it is relevant to my taking part in this research. I give permission for these individuals to have access to this data
6. I understand that a UCL-approved transcription company will have temporary access to the information I provide in the audio recordings and I agree for my sessions and interview with the researcher to be audiotaped *(Optional)*
7. I agree to be contacted again for future research studies *(Optional)*
8. I understand that the information I share, including anonymised direct quotes, may be included in any resulting report, publications and conference presentations *(Optional)*
9. I agree that the information collected about me can be used to support other research in the future, and may be shared anonymously with other researchers *(Optional)*
10. I would like to receive a summary of the research findings *(Optional)*
11. I agree to take part in the above study
12. I agree to share my mobile phone number to receive text reminders as part of the intervention and for my mobile number only to be transferred to a UCL-approved text messaging service

Name of Participant	Date	Signature
Name of Person taking consent	Date	Signature

DREAMS START FC_CONSENT_IRAS 272935_v2.1_20-DEC-2020 Page 1 of 1

Site/Centre Number:

Dyad Number:

Participant Identification Number:

[INSERT NHS TRUST LOGO HERE]

CONSULTEE DECLARATION FORM

Title: **DREAMS START: Dementia-related manual for sleep strategies for relatives**

Please initial box

1. I [insert name] have been consulted about [insert name of participant]'s participation in this research project. I confirm that I have read the information sheet dated 20-DEC-2020 (version 2.1) for the above study and understand the purpose of the research and I have received a copy of the information sheet and consultee declaration form to keep.
2. I have had the opportunity to consider the information, ask questions and have had these answered satisfactorily.
3. I understand that his/her participation is voluntary and that I am free to request that they are withdrawn from the study at any time without their legal or medical rights being affected.
4. I understand that his/her personal information will be processed only for the purposes of research and that all material and personal information will be kept in accordance with General Data Protection Regulation (GDPR) and the Data Protection Act 2018.
5. I understand that relevant data collected during the study may be looked at by individuals from the sponsor of the trial (Camden & Islington NHS Foundation Trust), UCL, regulatory authorities or from the NHS Trust, where it is relevant to their taking part in this research. I give permission for these individuals to have access to this data.
6. I agree for his/her GP being informed of their participation in the study, including any necessary exchange of information about him/her between their GP and the research team
7. I understand that an external UCL-approved transcription company will have temporary access to the information he/she provides in the audio recording and I agree for their sessions and interview with the researcher to be audiotaped. *(optional)*
8. I agree to he/she being contacted again for future research studies *(optional)*
9. I understand that the information he/shares, including anonymised direct quotes, may be included in any resulting report, publications and conference presentations *(optional)*
10. I understand that the information collected regarding he/she will be used to support other research in the future, and may be shared anonymously with other researchers *(optional)*
11. He/she would like to receive a summary of the research findings *(optional)*
12. In my opinion he/she would have no objection to taking part in the above study.

Name of Consultee ________ Relationship to participant ________ Date ________ Signature ________

Name of Person taking consent ________ Date ________ Signature ________

DREAMS START PCONSULTEE_CONSENT_IRAS 272935 _v2.1_20-DEC--2020 page 1 of 1

Site/Centre Number:

Dyad Number:

Participant Identification Number:

[INSERT TRUST LOGO HERE]

PERSON LIVING WITH DEMENTIA CONSENT FORM

Title: DREAMS START: Dementia-related manual for sleep strategies for relatives

Please initial box

1. I confirm that I have read the information sheet dated 20-DEC-2020 (version 2.1) for the above study and understand the purpose of the research and I have received a copy of the information sheet and consent form to keep.
2. I have had the opportunity to consider the information, ask questions and have had these answered satisfactorily
3. I understand that my participation is voluntary and that I am free to withdraw from the research study at any time without my legal rights or medical care being affected.
4. I agree that if I lose capacity to consent during the study, I would/I would not be willing for my relative to act as a personal legal representative and give consent on my behalf
5. I understand that that my personal information will be processed only for the purposes of research and that all material and personal information will be kept in accordance with General Data Protection Regulation (GDPR) and the Data Protection Act 2018.
6. I understand that relevant data collected during the study may be looked at by individuals from the sponsor of the trial (Camden & Islington NHS Foundation Trust), UCL, regulatory authorities or from the NHS Trust, where it is relevant to my taking part in this research. I give permission for these individuals to have access to this data.
7. I agree to my General Practitioner being informed of my participation in the study, including any necessary exchange of information about me between my GP and the research team
8. I understand that an external UCL-approved transcription company will have temporary access to the information I provide in the audio recordings I agree for my sessions and interview with the researcher to be audiotaped *(optional)*
9. I agree to be contacted again for future research studies *(optional)*
10. I understand that the information I share, including anonymised direct quotes, may be included in any resulting report, publications and conference presentations *(optional)*
11. I agree that the information collected about me can be used to support other research in the future, and may be shared anonymously with other researchers *(optional)*
12. I would like to receive a summary of the research findings *(optional)*
13. I agree to take part in the above study

Name of Participant	Date	Signature
Name of Person taking consent	Date	Signature

DREAMS START PLWD_CONSENT_IRAS 272935_v2.1_20-DEC-2020 Page 1 of 1
