# Supplementary material for: Clinical and cost-effectiveness of DREAMS START (Dementia RElAted Manual for Sleep; STrAtegies for RelaTives) for people living with dementia and their carers: a study protocol for a parallel multicentre randomised controlled trial
Source: BMJ Open. 2024 Feb 1;14(2):e075273. doi: 10.1136/bmjopen-2023-075273 (PMC10836385; doi:10.1136/bmjopen-2023-075273)
Supplement: Supplementary data [file bmjopen-2023-075273supp002.pdf]

Supplementary Table 1 Schedule of visits and assessments

	Screening / Baseline	Randomisation	Session 1	Session 2	Session 3	Session 4	Session 5	Session 6	4mth follow up	8mth follow up	+ 8month
Informed Consent	x										
Sleep disorders inventory	x								x	x	
Eligibility confirmation	x										
Demographics (person with dementia)	x										
Neuropsychiatric Inventory	x								x	x	
Epworth Sleepiness Scale	x								x	x	
DEMQOL-Proxy	x								x	x	
CSRI/medication (proxy, for person with dementia)	x								x	x	
EQ-5D-5L (proxy, for person with dementia)	x								x	x	
Side effects	x								x	x	
Actigraphy	x								x	x	
Demographics (carer)	x										
Sleep Condition Indicator (carer)	x										

Hospital Anxiety and Depression Scale (carer)	x								x	x	
Zarit Burden Interview (carer)	x								x	x	
Health Status Questionnaire (12) (carer)	x								x	x	
CSRI/medication (carer)	x								x	x	
EQ-5D-5L (carer)	x								x	x	
Randomisation		x									
DREAMS START intervention			x	x	x	x	x	x			
Intervention acceptability (qualitative interview)											x
