# Supplementary material for: Clinical and cost-effectiveness of DREAMS START (Dementia RElAted Manual for Sleep; STrAtegies for RelaTives) for people living with dementia and their carers: a study protocol for a parallel multicentre randomised controlled trial
Source: BMJ Open. 2024 Feb 1;14(2):e075273. doi: 10.1136/bmjopen-2023-075273 (PMC10836385; doi:10.1136/bmjopen-2023-075273)
Supplement: Supplementary data [file bmjopen-2023-075273supp003.pdf]

Appendix 2 Intervention components

Session/Section	Derived from
**Session 1**	
Sleep and dementia –	material provided by Simon D Kyle.
What is sleep? –	material provided by Simon D Kyle.
What causes sleep problems in dementia? –	written by Penny Rapaport, Simon D Kyle and Gill Livingston for DREAMS.
Making changes to improve sleep –	adapted from cognitive–behavioural therapy (CBT) work by Colin A Espie.
The impact of sleep problems on you and our relative	developed for DREAMS.
Managing the stress that sleep problems can bring –	adapted from START*.
Managing stress: the signal breath –	adapted from START.
Summary –	adapted from START.
Putting it into practice –	adapted from START.
**Session 2**	
Recap on understanding sleep and dementia.	
Establishing a good day and night routine	adapted from CBT work by Colin A Espie.
Your relative’s sleep pattern –	developed for DREAMS.
Light and sleep –	material provided by Simon D Kyle.
Light, dementia and the body clock –	material provided by Simon D Kyle.
Making a light therapy plan –	developed for DREAMS.
Making a new sleep routine: your relative’s plan –	developed for DREAMS based on work on sleep efficiency by Colin A Espie and Simon D Kyle.
Managing stress 2: focused breathing –	adapted from START.
Summary –	adapted from START.
Putting it into practice –	adapted from START.
**Session 3**	
Recap on making a plan.	
The importance of daytime activity and routine –	adapted from START.
Planning daytime activity –	adapted from START.

Sleep, exercise and physical activity –	developed for DREAMS by Penny Rapaport, Gill Livingston and Simon D Kyle.
Managing stress 3: guided imagery –	adapted from START.
Summary –	adapted from START.
Putting it into practice –	adapted from START.
Seated exercises visual guide –	from NHS Choices website.66
**Session 4**	
Recap on daytime activity and routine.	
Troubleshooting: putting plans into action –	developed for DREAMS.
Your relative's plan	developed for DREAMS.
Managing night-time behaviour problems –	adapted from MARQUE**/START.
Describing and investigating behaviours –	adapted from MARQUE/START.
Managing stress 4: stretching –	adapted from START.
Summary –	adapted from START.
Putting it into practice –	adapted from START.
**Session 5**	
Recap on night-time behaviour problems.	
Creating strategies for managing behaviours –	adapted from MARQUE.
Managing your own sleep –	developed for DREAMS.
Managing thoughts and feelings –	adapted from CBT work by Colin A Espie.
Challenging unhelpful thoughts and feelings –	adapted from START.
Making time for yourself	adapted from START.
Managing stress 5: guided imagery – ocean escape –	adapted from START.
Summary –	adapted from START.
Putting it into practice –	adapted from START.
**Session 6**	**Overall structure based on that developed in START and refined in MARQUE.**
Putting it all together.	
What works? Light, sleep and dementia –	written by Simon D Kyle for DREAMS.
What works? The importance of daytime activity –	adapted from START.

What works? Making a new sleep routine –	based on the sleep manual by Colin A Espie and Simon D Kyle.
What works? Making changes to improve sleep –	based on the sleep manual by Colin A Espie and Simon D Kyle.
What works? Managing night-time behaviours –	based on MARQUE/START.
What works? Challenging unhelpful thoughts and feelings –	based on START and CBT work by Colin A Espie.
What works? Relaxation –	adapted from START.
Keeping it going – developing an action plan –	developed for DREAMS.
Action plan for you and your relative –	developed for DREAMS.
Summary –	developed for DREAMS.

* STrAtegies for RelaTives (START) for further information see https://www.thelancet.com/journals/lanpsy/article/PIIS2215-0366(14)00073-X/fulltext

** Managing Agitation and Raising Quality of Life (MARQUE) for further information see https://pubmed.ncbi.nlm.nih.gov/30872010/
