# Supplementary material for: Clinical and cost-effectiveness of DREAMS START (Dementia RElAted Manual for Sleep; STrAtegies for RelaTives) for people living with dementia and their carers: a study protocol for a parallel multicentre randomised controlled trial
Source: BMJ Open. 2024 Feb 1;14(2):e075273. doi: 10.1136/bmjopen-2023-075273 (PMC10836385; doi:10.1136/bmjopen-2023-075273)
Supplement: Author's manuscript [file bmjopen-2023-075273.draft_revisions.pdf]

BMJ Open is committed to open peer review. As part of this commitment we make the peer review history of every article we publish publicly available.

When an article is published we post the peer reviewers' comments and the authors' responses online. We also post the versions of the paper that were used during peer review. These are the versions that the peer review comments apply to.

The versions of the paper that follow are the versions that were submitted during the peer review process. They are not the versions of record or the final published versions. They should not be cited or distributed as the published version of this manuscript.

BMJ Open is an open access journal and the full, final, typeset and author-corrected version of record of the manuscript is available on our site with no access controls, subscription charges or pay-per-view fees (http://bmjopen.bmj.com).

If you have any questions on BMJ Open's open peer review process please email info.bmjopen@bmj.com

BMJ Open

Clinical and cost-effectiveness of DREAMS START (Dementia RElAted Manual for Sleep; STrAtegies for RelaTives) for people living with dementia and their carers. A study protocol for a parallel multi-centre randomised controlled trial.

Journal:	*BMJ Open*
Manuscript ID	bmjopen-2023-075273
Article Type:	Protocol
Date Submitted by the Author:	02-May-2023
Complete List of Authors:	Rapaport, Penny; University College London, Division of Psychiatry<br>Amador, Sarah ; University College London, Division of Psychiatry<br>Adeleke, Mariam; University College London, Department of Statistical Science<br>Banerjee, Sube; Brighton and Sussex Medical School, Centre for Dementia Studies<br>Barber, Julie; University College London, Department of Statistical Science<br>Charlesworth, Georgina; University College London, Division of Psychology and Language Sciences; North East London NHS Foundation Trust<br>Clarke, Christopher; Tees Esk and Wear Valleys NHS Foundation Trust<br>Connell, Caroline; Tees Esk and Wear Valleys NHS Foundation Trust<br>Espie, Colin; University of Oxford, Sleep and Circadian Neuroscience Institute<br>Horsley, Rossana; Alzheimer's Society<br>Hunter, Rachael; University College London, Research Dept of Primary Care and Population Health<br>Kyle, Simon; University of Oxford, Sleep and Circadian Neuroscience Institute<br>Le Novere, Marie; UCL, PCPH<br>Manela, Monica; University College London, Division of Psychiatry<br>Morris, Sarah; Tees Esk and Wear Valleys NHS Foundation Trust<br>Pikett, Liam; University College London<br>Raczek, Malgorzata; BSMS Centre for Dementia Studies<br>Thornton, Emma; Tees Esk and Wear Valleys NHS Foundation Trust<br>Walker, Zuzana; University College London, Division of Psychiatry<br>Webster, Lucy; University College London, Division of Psychiatry; University of Kent<br>Livingston, Gill; University College London, Division of Psychiatry
Keywords:	Dementia, Health Services for the Aged, Clinical Trial

Clinical and cost-effectiveness of DREAMS START (Dementia RElAted Manual for Sleep; STrAtegies for RelaTives) for people living with dementia and their carers. A study protocol for a parallel multi-centre randomised controlled trial.

Corresponding author:
Dr Penny Rapaport
Principal Research Fellow / Clinical Psychologist*
Tel: 0207 679 9647
Email: p.rapaport@ucl.ac.uk

Authors:
Penny Rapaport[1]
Sarah Amador[1]
Mariam Adeleke[2]
Sube Banerjee[3]
Julie Barber[2]
Georgina Charlesworth[4]
Christopher Clarke[5]
Caroline Connell[6]
Colin A. Espie[7]
Rossana Horsley[8]
Rachael Hunter[9]
Simon Kyle[7]
Marie Le Novere[9]
Monica Manela[1]
Sarah Morris[5]
Liam Pikett[1]
Malgorzata Racze[6]
Emma Thornton[5]
Zuzana Walker[1]
Lucy Webster[1, 10]
Gill Livingston[1]

For peer review only

For peer review only - http://bmjopen.bmj.com/site/about/guidelines.xhtml

1 Department of Mental Health of Older People, Division of Psychiatry, University College London, London, UK

2 Department of Statistical Science, University College London, London, UK

3 Faculty of Health, University of Plymouth, England, UK

4 Division of Psychology and Language Sciences, University College London, London, UK and North East London NHS Foundation Trust, London, UK

5 Tees, Esk and Wear Valley NHS Foundation Trust , UK

6 BSMS Centre for Dementia Studies, Brighton, UK

7 Sir Jules Thorn Sleep & Circadian Neurosciences Institute, University of Oxford, UK

8 Patient and Public Involvement member

9 Department of Primary Care and Population Health, Institute of Epidemiology and Health Care, University College London, London, UK

10 University of Kent, UK

Word count: 3951

Tables: 1

Figures: 1

Supplementary materials:

Appendix 1: Informed consent form

Appendix 2: Summary of intervention components

ABSTRACT (291/300)

Introduction: Many people living with dementia experience sleep disturbance and there are no known effective treatments. Non-pharmacological treatment options should be first-line sleep management. For family carers relatives' sleep disturbance leads to interruption of their sleep, low mood, and breakdown of care. Our team developed and delivered DREAMS START (Dementia RElAted Manual for Sleep; STrAtegies for RelaTives), a multimodal non-pharmacological intervention, showing it to be feasible and acceptable. The aim of this randomised controlled trial (RCT) is to establish whether DREAMS START is clinically and cost-effective in reducing sleep disturbances in people living with dementia living at home compared to usual care.

Methods and analysis: We will recruit 370 participant dyads (people living with dementia and family carers) from memory services, community mental health teams and the Join Dementia Research

For peer review only - http://bmjopen.bmj.com/site/about/guidelines.xhtml

For peer review only

Website in England. Those meeting inclusion criteria will be randomised (1:1) either to DREAMS START or to usual treatment. DREAMS START is a six-session, manualised intervention delivered by supervised, non-clinically trained graduates. Outcomes will be collected at baseline, 4 months and 8 months with the primary outcome the Sleep Disorders Inventory (SDI) score at 8 months. Secondary outcomes for the person with dementia (all proxy) include quality of life, daytime sleepiness, neuropsychiatric symptoms and cost-effectiveness. Secondary outcomes for the family carer include quality of life, sleep disturbance, mood, burden and service use and caring/work activity. Analyses will be intention-to-treat and we will conduct a process evaluation.

Ethics and dissemination: London – Camden & Kings Cross Ethics Committee (20/LO/0894) approved the study. We will disseminate our findings in high-impact peer-reviewed journals and at national and international conferences. This research has the potential to improve sleep and quality of life for people living with dementia and their carers, in a feasible and scalable intervention.

Trial registration number: ISRCTN13072268

ARTICLE SUMMARY

Strengths and limitations of this study:

- This multi-centre randomised controlled trial recruiting 370 participants will be the only definitive randomised controlled trial of a multicomponent non-pharmacological intervention targeting sleep disturbance in dementia to date.
- The study will provide definitive clinical and cost-effectiveness evidence for the DREAMS START intervention.
- Robust cost-effectiveness data and a mixed-methods process evaluation will, if the intervention is effective, support the potential roll out of the intervention into NHS services.
- In our feasibility trial, we found that actigraphy had lower acceptability, and validity than needed as an outcome measure, we are therefore using proxy questionnaires to measure outcomes and using actigraphy as a measure of movement to inform our process evaluation.
- The nature of the intervention is such that participants and those delivering the intervention will not be blinded to treatment allocation.

For peer review only - http://bmjopen.bmj.com/site/about/guidelines.xhtml

For peer review only

INTRODUCTION

The 47 million people living with dementia worldwide is projected to increase to 131 million by 2050; at an estimated trillion United States Dollar (USD) cost(1). Sleep disturbance affects 25-40% of people living with dementia(2-6) with a meta-analysis finding a pooled prevalence of 24% among people living at home with Alzheimer's disease (AD) and 49% of those living at home with Lewy body dementia(6). Sleep disturbance affects all aspects of mental and physical functioning and quality of life(7) and may increase the risk of developing or worsening of AD(8). People living with dementia may wake during the night unaware of the time or be distressed or disorientated. Family members provide most care for the two-thirds of people living with dementia living at home(9), finding it difficult to cope with persistently disturbed sleep, impacting on quality of life(10, 11). Sleep disturbances also predict family carer depressive symptoms, burden and care home admission, elevating the individual, societal and economic impact of dementia(12-14).

As with other older people, most people living with dementia have other illnesses and over 90% have at least one long-term health condition and may experience pain, discomfort or mood disturbances(15) which can all contribute to sleep disturbance(16). Moreover, neurodegeneration of brain structures involved in the regulation of sleep and circadian rhythms, including the suprachiasmatic nucleus (SCN), likely mediate changes in sleep timing, sleep continuity, and sleep architecture in dementia(17). Dementia can therefore lead to impaired sleep initiation, reduced night-time sleep, difficulty maintaining sleep, increased night-time wandering, and excessive daytime sleepiness(2, 3, 18). Treating sleep problems may not only improve wellbeing, daytime functioning, and quality of life of those living with dementia but given the bi-directional relationship between sleep disruption and amyloid deposition and tau pathology(19) could potentially slow disease progression(20).

There is no conclusive evidence that any therapy to treat sleep disturbance in dementia is effective(11, 21). A Cochrane review of pharmacotherapies found no conclusive randomised controlled trial (RCT) evidence for people living with dementia(22). Antipsychotics and benzodiazepines have adverse effects(23) and increase mortality in older adults(24). Pharmacological interventions, including melatonin, are not recommended as treatments(25) and patients and their doctors prefer non-drug approaches for sleep problems(26). A Cochrane review found insufficient evidence for light therapy alone in dementia(27). As sleep disturbances in people living with dementia have mixed causes, promising interventions are likely to be multi-component. Although there is no definitive evidence of any effective non-pharmacological treatment for sleep disturbances in people living with dementia(28), two pilot studies in community-dwelling people

For peer review only

For peer review only - http://bmjopen.bmj.com/site/about/guidelines.xhtml

living with dementia, and our DREAMS START (Dementia RElAted Manual for Sleep; STrAtegies for RelaTives) feasibility RCT, found potential benefits of combining light with other components, including sleep education and hygiene, exercise, daytime activities and cognitive behavioural therapy (CBT) (29-31). We need clinically and cost-effective ways to improve disrupted sleep for people living with dementia, their families, health and social care systems, society and global economies.

This research builds on our successful feasibility RCT (NIHR/HTA 14/220/06)(29, 32). Our vision was to synthesise and add to incomplete evidence, co-produce a multi-component non-pharmacological intervention, and deliver it individually, tailoring it to fit each participant's needs. We co-produced DREAMS START using the best available evidence, patient and public involvement (PPI) and our clinical and research expertise in sleep and dementia(33). The intervention is delivered to family carers, who implement strategies to reduce the person with dementia's sleep disturbances. It uses natural daylight (where feasible) and if necessary, timed phototherapy to strengthen and stabilise sleep-wake timing. Additionally, it alerts carers to consider pain, uses strategies to increase comfort, reduce anxiety, increase daytime activity and uses CBT for sleep management, which Cochrane reviews found effective for older adults and family carers of people without dementia(34, 35). Our feasibility RCT found that the study design and the intervention were feasible and acceptable; 63/95 (65%; 95% CI 55% to 75%) eligible referrals consented, 62/95 (65%; 95% CI 55% to 75%) were randomised, and 37/42 (88%; 95% CI 75% to 96%) randomised to the intervention adhered to it. Qualitative interviews indicated that it was acceptable.

Study objectives

This study aims to determine whether our manualised, multi-component, non-pharmacological treatment package delivered by supervised non-clinically qualified psychology graduates, will deliver significant benefits for people living with dementia and their family carers. Additional aims are to assess the process and fidelity of delivery of the intervention and explore the experiences of family carers receiving the intervention. We will use the data generated to make evidence-based clinical practice recommendations on managing sleep disturbance in dementia.

Primary objective: To determine whether the DREAMS START intervention improves sleep disturbance in people living with dementia at home at 8 months compared to usual NHS treatment.

Secondary objectives: To determine:

1. Whether the DREAMS START intervention improves sleep disturbance in people living with dementia at home at 4-months.

For peer review only - http://bmjopen.bmj.com/site/about/guidelines.xhtml

2. Whether it reduces daytime sleepiness.
3. Whether it improves people living with dementia's quality of life.
4. Whether it is cost-effective.
5. The role of psychotropic medication and melatonin in any change.
6. Whether it improves family carer quality of life.
7. Whether it improves family carer's sleep and decreases their affective symptoms and burden.
8. The mechanisms of change
9. If effective, how we can optimise the intervention for implementation at scale in the NHS

METHODS AND ANALYSIS

Trial design

This is a multi-centre, parallel-group, superiority randomised controlled trial (RCT) with masked outcome assessment to test whether the DREAMS START intervention improves sleep disturbance in people living with dementia compared to usual treatment. Participants will be enrolled in the trial for eight months, assessments will take place at baseline, four months, and eight months post-randomisation (see Figure 1 for trial flow diagram). The trial is registered with the ISRCTN and an independent trial steering committee (TSC) and data monitoring and ethics committee (DMEC) provide oversight.

Participants and recruitment

Potential participant dyads (family carers and people living with dementia) will be recruited from memory services and older adult mental health services in NHS Trusts in London, Essex, Sussex, and Tees, Esk and Wear Valleys and the Join Dementia Research (JDR) website. JDR is a free, secure online and telephone service developed and launched in 2014/2015 by the National Institute of Health and Care Research (NIHR).

Eligibility will be checked by researchers during initial screening calls to potential participants. People will be included in the study if they meet the following criteria:

1. People living with dementia (any type/severity), except those currently drinking alcohol heavily.
2. Sleep disorders inventory (SDI) score ≥4. The SDI is a valid and reliable standalone tool for sleep disorder in people with dementia(36). Those who score ≥4 have clinically significant sleep disturbance.

For peer review only

3. Sleep that patient or their family judge as problematic. This is a pragmatic study and if the patient and family are unconcerned, treatment is unnecessary.
4. Patient with capacity gives consent, or if not capacitous, consultee gives consent with patient not unwilling.
5. Family carer gives informed consent.
6. Family carer supports the person with dementia emotionally or practically at least weekly.
7. Person with dementia lives in their own home at the beginning of the study with someone present at night.

Exclusion criteria (any):

1. Known primary sleep disorder diagnosis preceding dementia (e.g. sleep apnoea).
2. Current known heavy alcohol use (Alcohol Use Disorders Identification Test – Consumption (AUDIT C) Score ≥8).
3. People unavailable for >3 weeks (e.g. planned holiday or hospital admission) of intervention and follow-up.
4. Currently enrolled in another non-pharmacological dementia RCT.

If potential participants meet eligibility criteria, researchers will offer either a baseline home visit or if necessary, an appointment via telephone or video call. At this appointment, if potential participants are willing, researchers will obtain written informed consent (see Appendix 1) or where necessary (audio-recorded) verbal consent to take part in the study from family carers and people living with dementia who have capacity (or a personal consultee will sign for people living with dementia who lack capacity to consent). They will then complete the baseline assessments (see Assessments below, and Table 1).

Intervention

DREAMS START is a six-session manual-based intervention for carers of people living with dementia to make changes to improve their relatives' sleep(29, 37). We co-designed the intervention based on academic expertise and existing evidence, patient and public involvement (PPI) and clinician expertise(33) (see Appendix 2 for a summary of intervention components). The intervention provides information about sleep and dementia, supports carers to use practical *zeitgebers* (cues that influence the person's biological rhythms e.g. regular timing of bed and rising, morning wake-up light, regular mealtimes) and to establish adaptive stimulus control (e.g. pre-bed settling routine, management of wakeful episodes). It uses strategies to promote de-arousal at night (e.g. relaxation, bedroom comfort, no caffeine or alcohol pre-bed, relaxation) and daytime behavioural activation to

For peer review only - http://bmjopen.bmj.com/site/about/guidelines.xhtml

maintain alertness and reduce daytime naps. The intervention also focuses on helping carers to look after their own (sleep) health, incorporating aspects of CBT. We use actigraphic data from devices worn by participants with dementia at baseline to inform the personalisation of intervention strategies. Over the sessions, carers will build a personalised action plan to continue using after the sessions are completed. We will aim to deliver the sessions weekly or fortnightly depending on the availability of the carer but will be flexible to maximise adherence. We will deliver either in person, online via video call or by telephone depending on the preferences of the carer and Covid 19 restrictions.

Training and supervising facilitators: DREAMS START Facilitators will be non-clinically qualified psychology graduates who have participated in a two-day training programme delivered by the study team online. Training will focus on dementia and sleep-wake regulation, empathic listening skills, facilitating behaviour change, interpreting actigraphy, using supervision effectively and working collaboratively with family carers and people living with dementia. Facilitators will receive fortnightly group supervision with a clinical psychologist with additional individual supervision as requested by facilitators or the study team.

Usual treatment: We are comparing the new treatment with treatment as usual (TAU). Participants randomised to the new treatment will also receive usual care. TAU varies according to where the person with dementia is treated and their individual needs, but incorporates National Institute for Health and Care Excellence (NICE) guidelines for dementia and consists of assessment, diagnosis, symptomatic interventions, risk assessment and management, advice and information(25). There is currently no consistent approach to the treatment of sleep difficulties in dementia. We expect usual treatment to reflect that identified during our feasibility trial, where interventions were medical, psychological and social(29). During our feasibility trial, 45% of participants were prescribed one or more psychotropic medications, with 11% prescribed anxiolytic or hypnotic medication. We will not exclude those taking medication for sleep but will note psychotropic medication prescribed and taken.

Randomisation and blinding

Participants will be randomised following completion of baseline assessments. Randomisation will be provided by a web-based system (Sealed Envelope) at the level of the patient and will be blocked and stratified by site, with a 1:1 intervention:TAU ratio. The researchers collecting follow-up data will be masked to group allocations. The person delivering the intervention will be independent of the person who would be carrying out the follow-up assessments, who will be blinded to the

For peer review only - http://bmjopen.bmj.com/site/about/guidelines.xhtml

intervention allocation. Clinical supervision will be conducted in separate groups to avoid unmasking during discussions.

Assessments

The primary outcome is sleep disturbance at 8 months and will be measured using the SDI. The SDI is validated for measuring sleep disturbance in people living with dementia and describes the frequency and severity of sleep-disturbed behaviours. It is validated against clinical variables and used in recent promising pilot studies of non-pharmacological interventions(29-31, 38). The SDI will be collected at baseline, 4 months, and 8 months post-randomisation. All secondary outcome measures will be taken at baseline, 4 months, and 8 months (see Table 1). The SDI will be collected at baseline, 4 months, and 8 months post-randomisation. All secondary outcome measures will be taken at baseline, 4 months, and 8 months (see Table 1).

Secondary outcomes for the person living with dementia are all proxy measures to minimise burden. These include neuropsychiatric symptoms (Neuropsychiatric Inventory (NPI))(39), daytime sleepiness (Epworth sleepiness scale (ESS))(40), and quality of life (DEMQOL-Proxy)(41). A Modified Client Service Receipt Inventory (CSRI)(42) and EQ-5D 5 level (EQ-5D-5L)(43) proxy will be collected to inform the cost-effectiveness analysis and we will also collect details of medication including side effects. We will collect one week of actigraphy for the person living with dementia (using the Axivity AX3)(44))(44) from baseline and at 4 and 8-month follow-ups) to inform understandings of the mechanism of change of the intervention. For family carers, we will assess sleep disturbance (Sleep Condition Indicator (SCI))(45), carer mood (Hospital Anxiety and Depression Scale (HADS))(46), carer burden (Zarit Burden Interview (ZBI))(47) and health related quality of life (Health Status Questionnaire (HSQ-12))(48). To inform the cost-effectiveness analysis a Modified CSRI(42) incorporating the Valuation of Informal Care Questionnaire (iVICQ) a measure of carer time and activity and the Brief Work Productivity and Activity Impairment (WPAI) a measure of productivity loss as well as the EQ-5D-5L(43) will be collected at each time point.

Sample size calculation

We used the standard deviation (SD) of baseline SDI scores (15.74) and the correlation between baseline and follow-up measurements (0.57) observed in our feasibility trial for the power calculation(29). There is no published SDI minimum clinically important difference. We aim to detect a difference of ≥5.5 points, consistent with important differences identified through consultation with experts. This corresponds to a small to medium effect size of 0.35 and is realistic (an average difference of 5.6 was observed in feasibility work). To account for potential facilitator clustering in

For peer review only - http://bmjopen.bmj.com/site/about/guidelines.xhtml

the intervention arm, we assumed an intercluster correlation coefficient (ICC) as observed in earlier studies(49, 50) (0.03) and an average of 15 participants per facilitator. Based on these assumptions, to detect a difference in average SDI of 5.5 between intervention and TAU with power 90% and 5% significance requires 370 participants; 185 in each arm. This calculation allows for up to 15% drop out and includes an inflation for potential non-normality of SDI (as observed in feasibility work)(51). Calculation of sample size was carried out using STATA version 14.

Adverse events

All adverse events (except those listed below as expected) will be recorded in the medical records/case notes/source data in the first instance and will be recorded with clinical symptoms and accompanied by a simple, brief description of the event, including dates as appropriate. All adverse events will be recorded until the participant has completed the intervention.

All serious adverse events (except those listed below as expected) will be recorded in the case report form (CRF) and SAE log. This is because the intervention is not invasive, already has a well-known safety profile and therefore, the collection of AE data will not add any value to the safety profile of the intervention. We will collect information on any planned hospitalization at baseline and these will not be considered adverse events. Based on our feasibility work we expect few side effects specific to the intervention. The events listed below describe expected procedural/disease-related AEs/SAEs:

- Possible emotional distress or discomfort as a result of the questionnaires or intervention.
- Potential for falls as a result of increased physical activity or getting out and about more or because of other ageage-related conditions.
- For those with more severe ,dementia, death, hospitalisation due to common causes related to the condition (e.g. UTIs, delirium, common infections) or transition to a care home as a result of the progression of illness.

Analysis plan

Statistical analysis

For each randomised group we will summarise the primary outcome (SDI at 8-month) using means with standard deviations and medians with interquartile ranges. We will also graphically examine the distribution of the score. We will describe the effect of the intervention using the difference in mean SDI score calculated with a 95% confidence interval. This estimate will be obtained from a mixed effects multiple regression model adjusting for baseline SDI score and site and allowing for facilitator

For peer review only - http://bmjopen.bmj.com/site/about/guidelines.xhtml

clustering in the intervention arm(52). If assumptions of the regression model are violated, the SDI score will be analysed after appropriate transformation (e.g. a log transformation). We will calculate the intra-cluster correlation coefficient (with 95% confidence interval) to describe facilitator clustering.

In secondary analyses, we will examine repeated measurements of outcome at 4 and 8-month by treatment group using summary statistics and profile plots. We will extend the mixed effects model to include these repeated measurements and allow consideration of the effect of the intervention over time. We will carry out all analyses by intention to treat (ITT), comparing the groups as randomised. Analyses of secondary clinical outcomes will take a similar approach to the analysis of the primary outcome, with models adjusting for associated baseline measurement.

Where there is missing outcome data, analyses will be based on people with an available outcome and will therefore rely on an assumption that data is missing at random. We will describe the number (%) of participants with missing outcome in each group, look at reasons for missingness and consider characteristics of the patients excluded from the ITT analysis. In a sensitivity analysis, we will re-estimate the treatment effect, with additional adjustment for baseline predictors of missingness. Further analyses based on multiple imputation methods will be considered if appropriate.

Health economic analysis

We will calculate the incremental cost per QALY gained of DREAMS START plus TAU compared to TAU only, over 8 months from a health and social care cost perspective using the EQ-5D-5L proxy to calculate QALYs in line with NICE guidance. Secondary analyses will calculate QALYs using the DEMQOL and relevant tariff and include impact and cost of carers (paid as well as family and close others) out of pocket costs and relevant wider societal costs.

We will include the EQ-5D-5L (a generic measure of health-related quality of life) for carers in the economic evaluation. We will not measure carer productivity loss alone, some carers will be retired, so measuring productivity loss, which focuses on absenteeism from paid employment and presenteeism within paid employment, is not sufficient here. Instead, we will capture the equivalent of "productivity loss" in our carer population by measuring days off and changes in employment such as moving from full-time to part-time employment and retirement. This will be costed using the human capital approach. The most important component of this analysis will be the impact on carer time and healthcare resource use which will be measured using the Valuation of Informal Care Questionnaire and a modified CSRI respectively. We will also include the brief Work Productivity and

For peer review only - http://bmjopen.bmj.com/site/about/guidelines.xhtml

For peer review only

Activity Impairment (WPAI) questionnaire for caregivers, to ensure we fully capture productivity losses in carers that are in employment.

We will use the number of sessions attended, duration of the session and grade of the staff delivering the session to calculate participant-level costs of DREAMS START, in addition to the training and supervision cost. We modified the CSRI based on data from our feasibility study and will use costs from published sources to cost healthcare resource use.

Carers will complete the questionnaires about resource use, time spent caring and productivity loss in the past 4 months at baseline, 4-, and 8 -month. We will calculate QALYs as the area under the curve, adjusting for baseline, over 8 months using the EQ-5D-5L and DEMQOL-Proxy to calculate QALYs. We will report descriptive statistics for resource use, costs and QALYs for baseline, 4 and 8 -month. We will calculate the mean incremental costs and QALYs of DREAMS START compared to usual care over 8 months using patient-level data linear regression, adjusting for site and facilitator clustering in line with the statistical analysis plan, with 95% confidence intervals, cost-effectiveness planes and cost-effectiveness acceptability curves calculated using bootstrapping. We will conduct sensitivity analyses for any assumptions made. We will assess the level and type of missing data and in conjunction with the statistician determine the most appropriate method for accounting for missing data, which is likely to be multiple imputation using chained equations.

Process evaluation

Qualitative analysis

We will purposively sample and conduct qualitative interviews with 15-20 participants (selected for maximum variation, men and women, completers and non-completers, spouses and non-spouse family carers and people living with dementia, across sites) and 5 staff delivering the intervention. We will transcribe qualitative data verbatim, anonymise the transcripts and use NVivo 12. We will use a thematic analytic approach(53). The team will organise data into preliminary themes iteratively identifying similarities and differences in the data. We will triangulate qualitative data with fidelity ratings, intervention adherence (session attendance; action plans, strategy tried) and quantitative outcomes (including patterns in activity from actigraphy).

Quantitative analysis

In considering change mechanisms, the analysis will focus on movement as a potentially important indicator. We will consider rest-activity amplitude, reflecting the relative difference between the least active five hours (L5) and the most active ten hours (M10) in the day. These will be recorded via the actigraphs worn for a week at baseline and for a week at the 4 and 8--month follow-up points.

For peer review only - http://bmjopen.bmj.com/site/about/guidelines.xhtml

The mediating effect of these measures on our primary and secondary outcomes will be examined using the stepped approach described by Whittle et al (54). Models fitted in all steps will be mixed models that account for facilitator clustering.

Fidelity analysis

To analyse the fidelity of delivery of DREAMS START, we will assess the number of appointments delivered across all intervention participants. Checklists will be applied independently of the facilitator to a random selection of one recorded intervention session for each participant. A mean fidelity score will be produced by dividing the number of items on the checklist identified as being delivered, by the number of items on the checklist that should have been delivered per appointment, per researcher and across all appointments. We will adopt thresholds used in other intervention fidelity work: where 81–100% constitutes high fidelity, 51-80% is moderate fidelity and 50% or lower constitutes low fidelity(55).

Patient and public involvement

We are partnered by Alzheimer's Society and their research network volunteers, including our co-applicant RH, who has experience as a carer and will continue her input, having contributed to our earlier feasibility study and development of the grant application for this RCT. We have a virtual PPI group and PPI members on our TSC and Trial Management Group (TMG), to provide partnership and enhance the relevance, practicality and utility of our materials and findings. They have advised on the language and content of information sheets, contributed to the revision of the intervention having been integral to the initial intervention co-production and are working with us on knowledge exchange and dissemination activities.

ETHICS AND DISSEMINATION

London – Camden & Kings Cross Research Ethics Committee (Reference: 20/LO/0894) approved the study on 18th September 2020.

We will disseminate our findings in high-impact peer-reviewed journals and at national and international conferences. We will present findings in appropriate local forums for health and social care professionals; participants who have indicated they are interested in the results will be sent a lay summary. We are working with an extensive network of academic researchers, policymakers, stakeholders and PPI, acting as a 'Community of Interest' to advise on and ensure acceptability for the dissemination of the research, and to ensure both national and international reach. We aim to

For peer review only - http://bmjopen.bmj.com/site/about/guidelines.xhtml

For peer review only

produce an implementation toolkit alongside our DREAMS START intervention and facilitator training materials.

This proposed research has the potential to improve sleep and quality of life for people living with dementia and their family carers, in a feasible and scalable intervention, without medication side effects, and to elucidate the mechanisms of impact. If clinically effective, our intervention should be cost-effective: it is cheap and potentially will reduce the health and social care burden, particularly if it delays care home admission.

TRIAL STATUS

The trial commenced recruitment in February 2021 and completed recruitment in March 2023 with final primary outcome data collected by November 2023 and the final report submitted in May 2024.

Author's contributions

All authors made a substantial contribution to this work. PR drafted the manuscript. All authors were involved in revising the manuscript, giving final approval of the version to be published and agree to be accountable for all aspects of the work. GL and PR conceived of the study and acquired funding. GL, PR, JB, RH, CC, SB, MG, GC, LW, CE, SK, RH, SA contributed to developing the protocol and its implementation.

Funding Statement

This study/project is funded by the National Institute for Health and Care Research (NIHR) Health Technology Assessment Programme (NIHR HTA 128761). The views expressed are those of the author(s) and not necessarily those of the NIHR or the Department of Health and Social Care. SDK and CAE are supported by the NIHR Oxford Health Biomedical Research Centre.

For peer review only

Competing interests The present manuscript is supported by a National Institute for Health and Care Research (NIHR) Health Technology Assessment Programme (HTA) grant (NIHR HTA 128761)**.** SB declares grants from NIHR, CIHR, ESRC, HEE, ESPRC, Alzheimer's Society, and the Alzheimer's Association with no COI with current work. CE declares grants from NIHR-HTA, NIHR-EME, NIHR-BRC, Wellcome Trust with no COI with current work. MR declares a grant from NIHR ARC KSS with no COI with current work. Outside the submitted work SK declares non-financial support from Big Health Ltd. in the form of no cost access to the digital sleep improvement programme, Sleepio, for use in clinical research. Outside the submitted work CE declares stock/stock options and other salary contributions from Big Health Limited developers of Sleepio. Outside the submitted work SSB declares personal fees and non-financial support from Lilly, personal fees from Boehringer-Ingelheim, personal fees from Axovant, personal fees from Lundbeck, personal fees from Nutricia and honoraria from the Hamad Medical Service for lectures and talks. Outside the submitted work MR declares Honorarium for presentation on Lewy body dementias for GE. SB is a trustee of the Alzheimer's Society and NED at the Somerset NHS Foundation Trust. CE is deputy editor of the Journal of Sleep Research and on the editorial board of Sleep Medicine reviews.

Data Sharing statement

On completion of the RCT the data will be made available by the authors on request.

For peer review only

REFERENCES

1. Prince M, Wimo A, Guerchet M, Ali G, Wu Y, Prina M. World Alzheimer Report 2015. The global impact of dementia. An analysis of prevalence, incidence, cost & trends; Alzheimer's Disease International: London. 2015.
2. Dauvilliers Y. Insomnia in patients with neurodegenerative conditions. Sleep Medicine. 2007;8:S27-S34.
3. Moran M, Lynch CA, Walsh C, Coen R, Coakley D, Lawlor BA. Sleep disturbance in mild to moderate Alzheimer's disease. Sleep Medicine. 2005;6(4):347-52.
4. de Oliveira AM, Radanovic M, de Mello PC, Buchain PC, Vizzotto AD, Celestino DL, et al. Nonpharmacological Interventions to Reduce Behavioral and Psychological Symptoms of Dementia: A Systematic Review. Biomed Res Int. 2015;2015:218980.
5. Ooms S, Ju Y-E. Treatment of sleep disorders in dementia. Current treatment options in neurology. 2016;18(9):40.
6. Koren T, Fisher E, Webster L, Livingston G, Rapaport P. Prevalence of sleep disturbances in people with dementia living in the community: A systematic review and meta-analysis. Ageing Research Reviews. 2022:101782.
7. Kyle SD, Espie CA, Morgan K. "… Not just a minor thing, it is something major, which stops you from functioning daily": quality of life and daytime functioning in insomnia. Behavioral sleep medicine. 2010;8(3):123-40.
8. Ju Y-ES, Lucey BP, Holtzman DM. Sleep and Alzheimer disease pathology—a bidirectional relationship. Nature reviews Neurology. 2014;10(2):115.
9. Prince M, Knapp M, Guerchet M, McCrone P, Prina M, Comas-Herrera A, et al. Dementia UK: update. London: Alzheimer's Society; 2014.
10. Gehrman P, Gooneratne NS, Brewster GS, Richards KC, Karlawish J. Impact of Alzheimer disease patients' sleep disturbances on their caregivers. Geriatric Nursing. 2018;39(1):60-5.
11. Kinnunen KM, Vikhanova A, Livingston G. The management of sleep disorders in dementia: an update. Curr Opin Psychiatry. 2017;30(6):491-7.
12. Gehrman P, Gooneratne NS, Brewster GS, Richards KC, Karlawish J. Impact of Alzheimer disease patients' sleep disturbances on their caregivers. Geriatric Nursing. 2017.
13. McCurry SM, Logsdon RG, Teri L, Vitiello MV. Sleep disturbances in caregivers of persons with dementia: contributing factors and treatment implications. Sleep medicine reviews. 2007;11(2):143-53.
14. McCrae CS, Dzierzewski JM, McNamara JP, Vatthauer KE, Roth AJ, Rowe MA. Changes in sleep predict changes in affect in older caregivers of individuals with Alzheimer's dementia: a multilevel model approach. Journals of Gerontology Series B: Psychological Sciences and Social Sciences. 2014;71(3):458-62.
15. Browne J, Edwards DA, Rhodes KM, Brimicombe DJ, Payne RA. Association of comorbidity and health service usage among patients with dementia in the UK: a population-based study. BMJ open. 2017;7(3):e012546.
16. Parliamentary Office of Science and Technology (POST). POSTbrief 29: Sleep and Long-Term Health. UK: Houses of Parliament; 2018.
17. Wang C, Holtzman DM. Bidirectional relationship between sleep and Alzheimer's disease: role of amyloid, tau, and other factors. Neuropsychopharmacology. 2020;45(1):104-20.
18. Cagnin A, Fragiacomo F, Camporese G, Turco M, Bussè C, Ermani M, et al. Sleep-wake profile in dementia with Lewy bodies, Alzheimer's disease, and normal aging. Journal of Alzheimer's Disease. 2017;55(4):1529-36.
19. Mander BA, Winer JR, Jagust WJ, Walker MP. Sleep: A Novel Mechanistic Pathway, Biomarker, and Treatment Target in the Pathology of Alzheimer's Disease? Trends Neurosci. 2016;39(8):552-66.
20. Holth JK, Patel TK, Holtzman DM. Sleep in Alzheimer's Disease–beyond amyloid. Neurobiology of sleep and circadian rhythms. 2017;2:4-14.

For peer review only - http://bmjopen.bmj.com/site/about/guidelines.xhtml

21. Livingston G, Sommerlad A, Orgeta V, Costafreda SG, Huntley J, Ames D. Dementia prevention, intervention, and care. 2017;390:2673-734.
22. McCleery J, Sharpley AL. Pharmacotherapies for sleep disturbances in dementia. Cochrane Database of Systematic Reviews. 2020(11).
23. Berry SD, Placide SG, Mostofsky E, Zhang Y, Lipsitz LA, Mittleman MA, et al. Antipsychotic and benzodiazepine drug changes affect acute falls risk differently in the nursing home. Journals of Gerontology Series A: Biomedical Sciences and Medical Sciences. 2015;71(2):273-8.
24. Weich S, Pearce HL, Croft P, Singh S, Crome I, Bashford J, et al. Effect of anxiolytic and hypnotic drug prescriptions on mortality hazards: retrospective cohort study. Bmj. 2014;348:g1996.
25. National Institute for Health and Care Excellence (NICE). Dementia: assessment, management and support for people living with dementia and their carers. NICE guideline (NG97): Methods, evidence and recommendations. . 2018.
26. Dzierzewski JM, O'Brien EM, Kay D, McCrae CS. Tackling sleeplessness: psychological treatment options for insomnia in older adults. Nature and science of sleep. 2010;2:47.
27. Forbes D, Blake CM, Thiessen EJ, Peacock S, Hawranik P. Light therapy for improving cognition, activities of daily living, sleep, challenging behaviour, and psychiatric disturbances in dementia. The Cochrane Library. 2014.
28. Brown CA, Berry R, Tan MC, Khoshia A, Turlapati L, Swedlove F. A critique of the evidence base for non-pharmacological sleep interventions for persons with dementia. Dementia. 2013;12(2):210-37.
29. Livingston G, Barber JA, Kinnunen KM, Webster L, Kyle SD, Cooper C, et al. DREAMS-START (Dementia RElAted Manual for Sleep; STrAtegies for RelaTives) for people with dementia and sleep disturbances: a single-blind feasibility and acceptability randomized controlled trial. International psychogeriatrics. 2018:1-15.
30. Gibson RH, Gander PH, Dowell AC, Jones LM. Non-pharmacological interventions for managing dementia-related sleep problems within community dwelling pairs: A mixed-method approach. Dementia. 2017;16(8):967-84.
31. Tewary S, Cook N, Pandya N, McCurry SM. Pilot test of a six-week group delivery caregiver training program to reduce sleep disturbances among older adults with dementia (Innovative practice). Dementia. 2018;17(2):234-43.
32. Kinnunen K, P R, L W, J B, D KS, B H, et al. A manual-based intervention for carers of people with dementia and sleep disturbances: an acceptability and feasibility RCT. . Health Technol Assess. 2018;22(71).
33. Rapaport P, Webster L, Horsley R, Kyle SD, Kinnunen KM, Hallam B, et al. An intervention to improve sleep for people living with dementia: Reflections on the development and co-production of DREAMS:START (Dementia RElAted Manual for Sleep: STrAtegies for RelaTives). Dementia. 2018;17(8):976-89.
34. Montgomery P, Dennis JA. Cognitive behavioural interventions for sleep problems in adults aged 60+. The Cochrane Library. 2003.
35. Sivertsen B, Nordhus IH. Management of insomnia in older adults. The British Journal of Psychiatry. 2007;190(4):285-6.
36. Tractenberg RE, Singer CM, Cummings JL, Thal LJ. The Sleep Disorders Inventory: an instrument for studies of sleep disturbance in persons with Alzheimer's disease. Journal of sleep research. 2003;12(4):331-7.
37. Kinnunen KM, Rapaport P, Webster L, Barber J, Kyle SD, Hallam B, et al. A manual-based intervention for carers of people with dementia and sleep disturbances: an acceptability and feasibility RCT. Health Technology Assessment (Winchester, England). 2018;22(71):1-408.
38. McCurry SM, Pike KC, Vitiello MV, Logsdon RG, Larson EB, Teri L. Increasing walking and bright light exposure to improve sleep in community-dwelling persons with Alzheimer's Disease: results of a randomized, controlled trial. Journal of the American Geriatrics Society. 2011;59(8):1393-402.

For peer review only - http://bmjopen.bmj.com/site/about/guidelines.xhtml

39. Cummings JL. The Neuropsychiatric Inventory: assessing psychopathology in dementia patients. Neurology. 1997;48(5 Suppl 6):S10-6.
40. Johns MW. Sleepiness in different situations measured by the Epworth Sleepiness Scale. Sleep. 1994;17(8):703-10.
41. Smith SC, Lamping DL, Banerjee S, Harwood RH, Foley B, Smith P. Development of a new measure of health-related quality of life for people with dementia: DEMQOL. 2007;37:737-46.
42. Beecham J, Knapp M. Costing Psychiatric Interventions. In: Thornicroft G, Brewin CR, Wing J, editors. Measuring Mental Health Needs. London: Gaskell/Royal College of Psychiatrists; 1992.
43. Bryan S, Hardyman W, Bentham P, Buckley A, Laight A. Proxy completion of EQ-5D in patients with dementia. Quality of Life Research. 2005;14(1):107-18.
44. Clarke CL, Taylor J, Crighton LJ, Goodbrand JA, McMurdo ME, Witham MD. Validation of the AX3 triaxial accelerometer in older functionally impaired people. Aging clinical and experimental research. 2017;29(3):451-7.
45. Espie CA, Kyle SD, Hames P, Gardani M, Fleming L, Cape J. The Sleep Condition Indicator: a clinical screening tool to evaluate insomnia disorder. BMJ open. 2014;4(3):e004183.
46. Zigmond AS, Snaith RP. The hospital anxiety and depression scale. Acta psychiatrica scandinavica. 1983;67(6):361-70.
47. Zarit SH, Reever KE, Bach-Peterson J. Relatives of the impaired elderly: correlates of feelings of burden. Gerontologist. 1980;20(6):649-55.
48. Barry TL, Kaiser KL, Atwood JR. Reliability, validity, and scoring of the Health Status Questionnaire-12 version 2.0. J Nurs Meas. 2007;15(1):24-35.
49. Livingston G, Barber J, Rapaport P, Knapp M, Griffin M, King D. Clinical effectiveness of a manual based coping strategy programme (START, STrAtegies for RelaTives) in promoting the mental health of carers of family members with dementia: pragmatic randomised controlled trial. 2013;347:f6276.
50. Livingston G, Barber J, Marston L, Stringer A, Panca M, Hunter R, et al. Clinical and cost-effectiveness of the Managing Agitation and Raising Quality of Life (MARQUE) intervention for agitation in people with dementia in care homes: a single-blind, cluster-randomised controlled trial. The Lancet Psychiatry. 2019.
51. RH R, DA. W. Introduction to the theory of nonparametric statistics. : Krieger; 1991.
52. Walwyn R, Roberts C. Therapist variation within randomised trials of psychotherapy: implications for precision, internal and external validity. Statistical Methods in Medical Research. 2010;19(3):291-315.
53. Braun V, Clarke V. Using thematic analysis in psychology. Qualitative Research in Psychology. 2006;3(2):77-101.
54. Whittle R, Mansell G, Jellema P, van der Windt D. Applying causal mediation methods to clinical trial data: What can we learn about why our interventions (don't) work? European Journal of Pain. 2017;21(4):614-22.
55. Noell GG, FM; Gansle, K. Does treatment integrity matter? A preliminary investigation of instructional implementation and mathematical performance. Journal of Behavioral Education 2002;11:15.

For peer review only - http://bmjopen.bmj.com/site/about/guidelines.xhtml

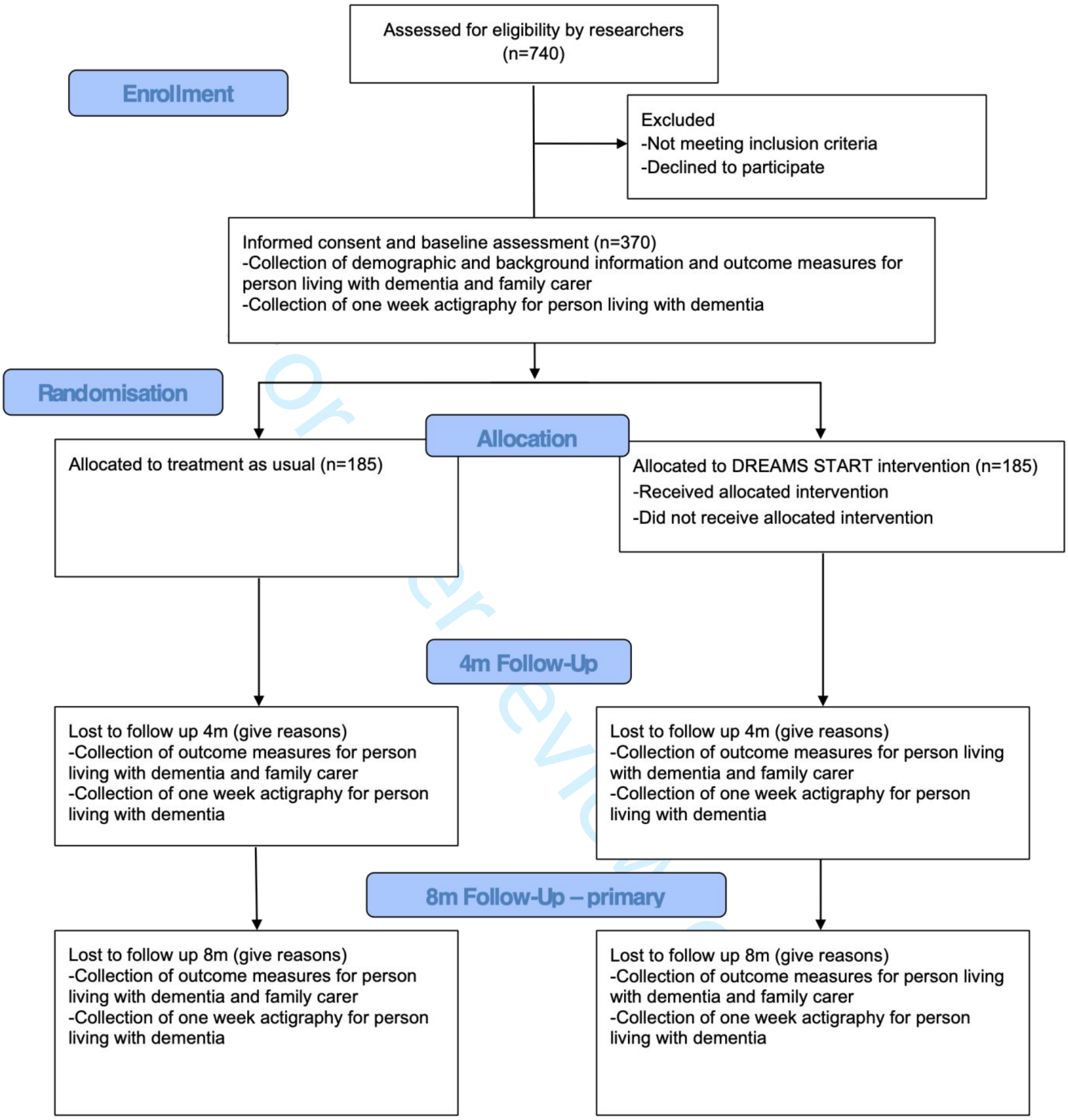

Figure 1: DREAMS START RCT flow diagram

For peer review only - http://bmjopen.bmj.com/site/about/guidelines.xhtml

Table 1: Schedule of visits and assessments

	Screening / Baseline	Randomisation	Session 1	Session 2	Session 3	Session 4	Session 5	Session 6	4mth follow up	8mth follow up	+ 8month
Informed Consent	x										
Sleep disorders inventory	x								x	x	
Eligibility confirmation	x										
Demographics (person with dementia)	x										
Neuropsychiatric Inventory	x								x	x	
Epworth Sleepiness Scale	x								x	x	
DEMQOL-Proxy	x								x	x	
CSRI/medication (proxy, for person with dementia)	x								x	x	
EQ-5D-5L (proxy, for person with dementia)	x								x	x	
Side effects	x								x	x	

For peer review only

Actigraphy	x								x	x	
Demographics (carer)	x										
Sleep Condition Indicator (carer)	x										
Hospital Anxiety and Depression Scale (carer)	x								x	x	
Zarit Burden Interview (carer)	x								x	x	
Health Status Questionnaire (12) (carer)	x								x	x	
CSRI/medication (carer)	x								x	x	
EQ-5D-5L (carer)	x								x	x	
Randomisation		x									
DREAMS START intervention			x	x	x	x	x	x			
Intervention acceptability (qualitative interview)											x

For peer review only

For peer review only - http://bmjopen.bmj.com/site/about/guidelines.xhtml

Appendix 1 Informed consent forms

**Site/Centre Number:**
**Dyad Number:**
**Participant Identification Number:**

UCL

[INSERT NHS TRUST LOGO HERE]

FAMILY CARER CONSENT FORM

Title: DREAMS START: Dementia-related manual for sleep strategies for relatives

Name of Researcher:

Please initial box

1. I confirm that I have read the information sheet dated 20-DEC-2020 (version 2.1) for the above study and understand the purpose of the research and I have received a copy of the information sheet and informed consent form to keep ☐
2. I have had the opportunity to consider the information, ask questions and have had these answered satisfactorily ☐
3. I understand that my participation is voluntary and that I am free to withdraw from the research study at any time without my legal rights or medical care being affected. ☐
4. I understand that that my personal information will be processed only for the purposes of research and that all material and personal information will be kept in accordance with General Data Protection Regulation (GDPR) and the Data Protection Act 2018. ☐
5. I understand that relevant data collected during the study may be looked at by individuals from the sponsor of the trial (Camden & Islington NHS Foundation Trust), UCL, regulatory authorities or from the NHS Trust, where it is relevant to my taking part in this research. I give permission for these individuals to have access to this data ☐
6. I understand that a UCL-approved transcription company will have temporary access to the information I provide in the audio recordings and I agree for my sessions and interview with the researcher to be audiotaped *(Optional)* ☐
7. I agree to be contacted again for future research studies *(Optional)* ☐
8. I understand that the information I share, including anonymised direct quotes, may be included in any resulting report, publications and conference presentations *(Optional)* ☐
9. I agree that the information collected about me can be used to support other research in the future, and may be shared anonymously with other researchers *(Optional)* ☐
10. I would like to receive a summary of the research findings *(Optional)* ☐
11. I agree to take part in the above study ☐
12. I agree to share my mobile phone number to receive text reminders as part of the intervention and for my mobile number only to be transferred to a UCL-approved text messaging service ☐

Name of Participant	Date	Signature
Name of Person taking consent	Date	Signature

DREAMS START FC_CONSENT_IRAS 272935_v2.1_20-DEC-2020 Page 1 of 1

For peer review only - http://bmjopen.bmj.com/site/about/guidelines.xhtml

**Site/Centre Number:**
**Dyad Number:**
**Participant Identification Number:**

[INSERT NHS TRUST LOGO HERE]

CONSULTEE DECLARATION FORM

Title: DREAMS START: Dementia-related manual for sleep strategies for relatives

Please initial box

1. I [insert name] have been consulted about [insert name of participant]'s participation in this research project. I confirm that I have read the information sheet dated 20-DEC-2020 (version 2.1) for the above study and understand the purpose of the research and I have received a copy of the information sheet and consultee declaration form to keep.
2. I have had the opportunity to consider the information, ask questions and have had these answered satisfactorily.
3. I understand that his/her participation is voluntary and that I am free to request that they are withdrawn from the study at any time without their legal or medical rights being affected.
4. I understand that his/her personal information will be processed only for the purposes of research and that all material and personal information will be kept in accordance with General Data Protection Regulation (GDPR) and the Data Protection Act 2018.
5. I understand that relevant data collected during the study may be looked at by individuals from the sponsor of the trial (Camden & Islington NHS Foundation Trust), UCL, regulatory authorities or from the NHS Trust, where it is relevant to their taking part in this research. I give permission for these individuals to have access to this data.
6. I agree for his/her GP being informed of their participation in the study, including any necessary exchange of information about him/her between their GP and the research team
7. I understand that an external UCL-approved transcription company will have temporary access to the information he/she provides in the audio recording and I agree for their sessions and interview with the researcher to be audiotaped. *(optional)*
8. I agree to he/she being contacted again for future research studies *(optional)*
9. I understand that the information he/shares, including anonymised direct quotes, may be included in any resulting report, publications and conference presentations *(optional)*
10. I understand that the information collected regarding he/she will be used to support other research in the future, and may be shared anonymously with other researchers *(optional)*
11. He/she would like to receive a summary of the research findings *(optional)*
12. In my opinion he/she would have no objection to taking part in the above study.

Name of Consultee Relationship to participant Date Signature

Name of Person taking consent Date Signature

DREAMS START PCONSULTEE_CONSENT_IRAS 272935 _v2.1_20-DEC--2020 page 1 of 1

Site/Centre Number:

Dyad Number:

Participant Identification Number:

[INSERT TRUST LOGO HERE]

PERSON LIVING WITH DEMENTIA CONSENT FORM

Title: DREAMS START: Dementia-related manual for sleep strategies for relatives

Please initial box

1. I confirm that I have read the information sheet dated 20-DEC-2020 (version 2.1) for the above study and understand the purpose of the research and I have received a copy of the information sheet and consent form to keep. ☐
2. I have had the opportunity to consider the information, ask questions and have had these answered satisfactorily ☐
3. I understand that my participation is voluntary and that I am free to withdraw from the research study at any time without my legal rights or medical care being affected. ☐
4. I agree that if I lose capacity to consent during the study, I would/I would not be willing for my relative to act as a personal legal representative and give consent on my behalf ☐
5. I understand that that my personal information will be processed only for the purposes of research and that all material and personal information will be kept in accordance with General Data Protection Regulation (GDPR) and the Data Protection Act 2018. ☐
6. I understand that relevant data collected during the study may be looked at by individuals from the sponsor of the trial (Camden & Islington NHS Foundation Trust), UCL, regulatory authorities or from the NHS Trust, where it is relevant to my taking part in this research. I give permission for these individuals to have access to this data. ☐
7. I agree to my General Practitioner being informed of my participation in the study, including any necessary exchange of information about me between my GP and the research team ☐
8. I understand that an external UCL-approved transcription company will have temporary access to the information I provide in the audio recordings I agree for my sessions and interview with the researcher to be audiotaped *(optional)* ☐
9. I agree to be contacted again for future research studies *(optional)* ☐
10. I understand that the information I share, including anonymised direct quotes, may be included in any resulting report, publications and conference presentations *(optional)* ☐
11. I agree that the information collected about me can be used to support other research in the future, and may be shared anonymously with other researchers *(optional)* ☐
12. I would like to receive a summary of the research findings *(optional)* ☐
13. I agree to take part in the above study ☐

Name of Participant	Date	Signature
Name of Person taking consent	Date	Signature

DREAMS START PLWD_CONSENT_IRAS 272935_v2.1_20-DEC-2020 Page 1 of 1

Appendix 2 Intervention components

SESSION/SECTION	DERIVED FROM
**SESSION 1**	
Sleep and dementia –	material provided by Simon D Kyle.
What is sleep? –	material provided by Simon D Kyle.
What causes sleep problems in dementia? –	written by Penny Rapaport, Simon D Kyle and Gill Livingston for DREAMS.
Making changes to improve sleep –	adapted from cognitive–behavioural therapy (CBT) work by Colin A Espie.
The impact of sleep problems on you and our relative	developed for DREAMS.
Managing the stress that sleep problems can bring –	adapted from START*.
Managing stress: the signal breath –	adapted from START.
Summary –	adapted from START.
Putting it into practice –	adapted from START.
**SESSION 2**	
Recap on understanding sleep and dementia.	
Establishing a good day and night routine	adapted from CBT work by Colin A Espie.
Your relative's sleep pattern –	developed for DREAMS.
Light and sleep –	material provided by Simon D Kyle.
Light, dementia and the body clock –	material provided by Simon D Kyle.
Making a light therapy plan –	developed for DREAMS.
Making a new sleep routine: your relative's plan –	developed for DREAMS based on work on sleep efficiency by Colin A Espie and Simon D Kyle.
Managing stress 2: focused breathing –	adapted from START.
Summary –	adapted from START.
Putting it into practice –	adapted from START.
**SESSION 3**	
Recap on making a plan.	
The importance of daytime activity and routine –	adapted from START.
Planning daytime activity –	adapted from START.
Sleep, exercise and physical activity –	developed for DREAMS by Penny Rapaport, Gill Livingston and Simon D Kyle.
Managing stress 3: guided imagery –	adapted from START.
Summary –	adapted from START.
Putting it into practice –	adapted from START.
Seated exercises visual guide –	from NHS Choices website.66
**SESSION 4**	
Recap on daytime activity and routine.	
Troubleshooting: putting plans into action –	developed for DREAMS.
Your relative's plan	developed for DREAMS.
Managing night-time behaviour problems –	adapted from MARQUE**/START.
Describing and investigating behaviours –	adapted from MARQUE/START.
Managing stress 4: stretching –	adapted from START.
Summary –	adapted from START.
Putting it into practice –	adapted from START.
**SESSION 5**	
Recap on night-time behaviour problems.	

For peer review only - http://bmjopen.bmj.com/site/about/guidelines.xhtml

Creating strategies for managing behaviours –	adapted from MARQUE.
Managing your own sleep –	developed for DREAMS.
Managing thoughts and feelings –	adapted from CBT work by Colin A Espie.
Challenging unhelpful thoughts and feelings –	adapted from START.
Making time for yourself	adapted from START.
Managing stress 5: guided imagery – ocean escape –	adapted from START.
Summary –	adapted from START.
Putting it into practice –	adapted from START.
**SESSION 6**	**Overall structure based on that developed in START and refined in MARQUE.**
Putting it all together.	
What works? Light, sleep and dementia –	written by Simon D Kyle for DREAMS.
What works? The importance of daytime activity –	adapted from START.
What works? Making a new sleep routine –	based on the sleep manual by Colin A Espie and Simon D Kyle.
What works? Making changes to improve sleep –	based on the sleep manual by Colin A Espie and Simon D Kyle.
What works? Managing night-time behaviours –	based on MARQUE/START.
What works? Challenging unhelpful thoughts and feelings –	based on START and CBT work by Colin A Espie.
What works? Relaxation –	adapted from START.
Keeping it going – developing an action plan –	developed for DREAMS.
Action plan for you and your relative –	developed for DREAMS.
Summary –	developed for DREAMS.

* STrAtegies for RelaTives (START) for further information see https://www.thelancet.com/journals/lanpsy/article/PIIS2215-0366(14)00073-X/fulltext
** Managing Agitation and Raising Quality of Life (MARQUE) for further information see https://pubmed.ncbi.nlm.nih.gov/30872010/

For peer review only - http://bmjopen.bmj.com/site/about/guidelines.xhtml

Reporting checklist for randomised trial.

Based on the CONSORT guidelines.

Instructions to authors

Complete this checklist by entering the page numbers from your manuscript where readers will find each of the items listed below.

Your article may not currently address all the items on the checklist. Please modify your text to include the missing information. If you are certain that an item does not apply, please write "n/a" and provide a short explanation.

Upload your completed checklist as an extra file when you submit to a journal.

In your methods section, say that you used the CONSORTreporting guidelines, and cite them as:

Schulz KF, Altman DG, Moher D, for the CONSORT Group. CONSORT 2010 Statement: updated guidelines for reporting parallel group randomised trials

		Reporting Item	Page Number
**Title and Abstract**			
Title	#1a	Identification as a randomized trial in the title.	1
Abstract	#1b	Structured summary of trial design, methods, results, and conclusions	2
**Introduction**			

For peer review only

Background and objectives	#2a	Scientific background and explanation of rationale	4
Background and objectives	#2b	Specific objectives or hypothesis	5
**Methods**			
Trial design	#3a	Description of trial design (such as parallel, factorial) including allocation ratio.	6
Trial design	#3b	Important changes to methods after trial commencement (such as eligibility criteria), with reasons	n/a
Participants	#4a	Eligibility criteria for participants	6
Participants	#4b	Settings and locations where the data were collected	6
Interventions	#5	The experimental and control interventions for each group with sufficient details to allow replication, including how and when they were actually administered	7
Outcomes	#6a	Completely defined prespecified primary and secondary outcome measures, including how and when they were assessed	9
Outcomes	#6b	Any changes to trial outcomes after the trial commenced, with reasons	n/a
Sample size	#7a	How sample size was determined.	9

For peer review only

Sample size	#7b	When applicable, explanation of any interim analyses and stopping guidelines	n/a
Randomization – Sequence generation	#8a	Method used to generate the random allocation sequence 8.	
Randomization - Sequence generation	#8b	Type of randomization; details of any restriction (such as blocking and block size) 8	
Randomization - Allocation concealment mechanism	#9	Mechanism used to implement the random allocation sequence (such as sequentially numbered containers), describing any steps taken to conceal the sequence until interventions were assigned	n/a
Randomization - Implementation	#10	Who generated the allocation sequence, who enrolled participants, and who assigned participants to interventions	8
Blinding	#11a	If done, who was blinded after assignment to interventions (for example, participants, care providers, those assessing outcomes) and how.	8
Blinding	#11b	If relevant, description of the similarity of interventions	n/a
Statistical methods	#12a	Statistical methods used to compare groups for primary and secondary outcomes	10/11
Statistical methods	#12b	Methods for additional analyses, such as subgroup analyses and adjusted analyses	10/11

Results

For peer review only

Participant flow diagram (strongly recommended)	#13a	For each group, the numbers of participants who were randomly assigned, received intended treatment, and were analysed for the primary outcome	19
Participant flow	#13b	For each group, losses and exclusions after randomization, together with reason	n/a
Recruitment	#14a	Dates defining the periods of recruitment and follow-up	n/a
Recruitment	#14b	Why the trial ended or was stopped	n/a
Baseline data	#15	A table showing baseline demographic and clinical characteristics for each group	n/a
Numbers analysed	#16	For each group, number of participants (denominator) included in each analysis and whether the analysis was by original assigned groups	n/a
Outcomes and estimation	#17a	For each primary and secondary outcome, results for each group, and the estimated effect size and its precision (such as 95% confidence interval)	n/a
Outcomes and estimation	#17b	For binary outcomes, presentation of both absolute and relative effect sizes is recommended	n/a
Ancillary analyses	#18	Results of any other analyses performed, including subgroup analyses and adjusted analyses, distinguishing pre-specified from exploratory	n/a
Harms	#19	All important harms or unintended effects in each group (For specific guidance see CONSORT for harms)	n/a

For peer review only

Discussion

Limitations	#20	Trial limitations, addressing sources of potential bias, imprecision, and, if relevant, multiplicity of analyses	n/a
Generalisability	#21	Generalisability (external validity, applicability) of the trial findings	n/a
Interpretation	#22	Interpretation consistent with results, balancing benefits and harms, and considering other relevant evidence	n/a
Registration	#23	Registration number and name of trial registry	3

Other information

Interpretation	#22	Interpretation consistent with results, balancing benefits and harms, and considering other relevant evidence	n/a
Registration	#23	Registration number and name of trial registry	3
Protocol	#24	Where the full trial protocol can be accessed, if available	n/a
Funding	#25	Sources of funding and other support (such as supply of drugs), role of funders	4

None The CONSORT checklist is distributed under the terms of the Creative Commons Attribution License CC-BY. This checklist can be completed online using https://www.goodreports.org/, a tool made by the EQUATOR Network in collaboration with Penelope.ai

For peer review only

BMJ Open

Clinical and cost-effectiveness of DREAMS START (Dementia RElAted Manual for Sleep; STrAtegies for RelaTives) for people living with dementia and their carers. A study protocol for a parallel multi-centre randomised controlled trial.

Journal:	*BMJ Open*
Manuscript ID	bmjopen-2023-075273.R1
Article Type:	Protocol
Date Submitted by the Author:	21-Dec-2023
Complete List of Authors:	Rapaport, Penny; University College London, Division of Psychiatry<br>Amador, Sarah ; University College London, Division of Psychiatry<br>Adeleke, Mariam; University College London, Department of Statistical Science<br>Banerjee, Sube; Brighton and Sussex Medical School, Centre for Dementia Studies<br>Barber, Julie; University College London, Department of Statistical Science<br>Charlesworth, Georgina; University College London, Division of Psychology and Language Sciences; North East London NHS Foundation Trust<br>Clarke, Christopher; Tees Esk and Wear Valleys NHS Foundation Trust<br>Connell, Caroline; Tees Esk and Wear Valleys NHS Foundation Trust<br>Espie, Colin; University of Oxford, Sleep and Circadian Neuroscience Institute<br>Gonzalez, Lina; University College London, Research Department of Primary Care and Population Health<br>Horsley, Rossana; Alzheimer's Society<br>Hunter, Rachael; University College London, Research Dept of Primary Care and Population Health<br>Kyle, Simon; University of Oxford, Sleep and Circadian Neuroscience Institute<br>Manela, Monica; University College London, Division of Psychiatry<br>Morris, Sarah; Tees Esk and Wear Valleys NHS Foundation Trust<br>Pikett, Liam; University College London<br>Raczek, Malgorzata; BSMS Centre for Dementia Studies<br>Thornton, Emma; Tees Esk and Wear Valleys NHS Foundation Trust<br>Walker, Zuzana; University College London, Division of Psychiatry<br>Webster, Lucy; University College London, Division of Psychiatry; University of Kent<br>Livingston, Gill; University College London, Division of Psychiatry
**Primary Subject Heading**:	Neurology
Secondary Subject Heading:	Mental health

For peer review only - http://bmjopen.bmj.com/site/about/guidelines.xhtml

Keywords:	Dementia, Health Services for the Aged, Clinical Trial

SCHOLARONE™
Manuscripts

Clinical and cost-effectiveness of DREAMS START (Dementia RElAted Manual for Sleep; STrAtegies for RelaTives) for people living with dementia and their carers. A study protocol for a parallel multi-centre randomised controlled trial.

Corresponding author:
Dr Penny Rapaport
Principal Research Fellow / Clinical Psychologist*
Tel: 0207 679 9647
Email: p.rapaport@ucl.ac.uk

Authors:
Penny Rapaport[1]
Sarah Amador[1]
Mariam Adeleke[2]
Sube Banerjee[3]
Julie Barber[2]
Georgina Charlesworth[4]
Christopher Clarke[5]
Caroline Connell[6]
Colin A. Espie[7]
Lina Gonzalez[9]
Rossana Horsley[8]
Rachael Hunter[9]
Simon D Kyle[7]
Monica Manela[1]
Sarah Morris[5]
Liam Pikett[1]
Malgorzata Raczek[6]
Emma Thornton[5]
Zuzana Walker[1]
Lucy Webster[1, 10]
Gill Livingston[1]

For peer review only

1 Department of Mental Health of Older People, Division of Psychiatry, University College London, London, UK
2 Department of Statistical Science, University College London, London, UK
3 Faculty of Health, University of Plymouth, England, UK
4 Division of Psychology and Language Sciences, University College London, London, UK and North East London NHS Foundation Trust, London, UK
5 Tees, Esk and Wear Valley NHS Foundation Trust , UK
6 BSMS Centre for Dementia Studies, Brighton, UK
7 Sir Jules Thorn Sleep & Circadian Neurosciences Institute, University of Oxford, UK
8 Patient and Public Involvement member
9 Department of Primary Care and Population Health, Institute of Epidemiology and Health Care, University College London, London, UK
10 University of Kent, UK

Word count: 5418

Figures: 2

Supplemental materials:

Supplementary Table 1: Schedule of visits and assessments

Appendix 1: Informed consent form

Appendix 2: Summary of intervention components

ABSTRACT (299/300)

Introduction: Many people living with dementia experience sleep disturbance and there are no known effective treatments. Non-pharmacological treatment options should be first-line sleep management. For family carers relatives' sleep disturbance leads to interruption of their sleep, low mood, and breakdown of care. Our team developed and delivered DREAMS START (Dementia RElAted Manual for Sleep; STrAtegies for RelaTives), a multimodal non-pharmacological intervention, showing it to be feasible and acceptable. The aim of this randomised controlled trial (RCT) is to establish whether DREAMS START is clinically and cost-effective in reducing sleep disturbances in people living with dementia living at home compared to usual care.

For peer review only

Methods and analysis: We will recruit 370 participant dyads (people living with dementia and family carers) from memory services, community mental health teams and the Join Dementia Research Website in England. Those meeting inclusion criteria will be randomised (1:1) either to DREAMS START or to usual treatment. DREAMS START is a six-session (1hour/session), manualised intervention delivered every 1-2 weeks by supervised, non-clinically trained graduates. Outcomes will be collected at baseline, 4 months and 8 months with the primary outcome the Sleep Disorders Inventory (SDI) score at 8 months. Secondary outcomes for the person with dementia (all proxy) include quality of life, daytime sleepiness, neuropsychiatric symptoms and cost-effectiveness. Secondary outcomes for the family carer include quality of life, sleep disturbance, mood, burden and service use and caring/work activity. Analyses will be intention-to-treat and we will conduct a process evaluation.

Ethics and dissemination: London – Camden & Kings Cross Ethics Committee (20/LO/0894) approved the study. We will disseminate our findings in high-impact peer-reviewed journals and at national and international conferences. This research has the potential to improve sleep and quality of life for people living with dementia and their carers, in a feasible and scalable intervention.

Trial registration number: ISRCTN13072268

ARTICLE SUMMARY

Strengths and limitations of this study:

- This multi-centre randomised controlled trial recruiting 370 participants will be the largest, and only fully powered randomised controlled trial of a multicomponent non-pharmacological intervention targeting sleep disturbance in people living at home with dementia to date.
- The study will provide conclusive clinical and cost-effectiveness evidence for the DREAMS START intervention.
- Robust cost-effectiveness data and a mixed-methods process evaluation will, if the intervention is effective, support the potential roll out of the intervention into UK NHS services and other international care settings.
- In our feasibility trial, we found that actigraphy had lower acceptability, and validity than needed as an outcome measure, we are therefore using proxy questionnaires to measure outcomes and using actigraphy as a measure of movement to inform our process evaluation.
- The nature of the intervention is such that participants and those delivering the intervention will not be blinded to treatment allocation.

For peer review only

1

For peer review only

INTRODUCTION

The 47 million people living with dementia worldwide is projected to increase to 131 million by 2050; at an estimated trillion United States Dollar (USD) cost(1). Sleep disturbance affects 25-40% of people living with dementia(2-6) with a meta-analysis finding a pooled prevalence of 24% among people living at home with Alzheimer's disease (AD) and 49% of those living at home with Lewy body dementia(6). Sleep disturbance affects all aspects of mental and physical functioning and quality of life(7) and may increase the risk of developing or worsening of AD(8). People living with dementia may wake during the night unaware of the time or be distressed or disorientated. Family members provide most care for the two-thirds of people living with dementia living at home(9), finding it difficult to cope with persistently disturbed sleep, impacting on quality of life(10, 11). Sleep disturbances also predict family carer depressive symptoms, burden and care home admission, elevating the individual, societal and economic impact of dementia(12-14). Although there is not currently data available for reliable estimates for the costs associated with sleep disturbances and dementia(15), we do know that sleep disturbances can be a contributing factor to transition to care homes which increases health and social care resource costs. It may also increase the likelihood of falls and loss of weight as people are more tired during the day and this may increase use of resources(16).

As with other older people, most people living with dementia have other illnesses and over 90% have at least one long-term health condition and may experience pain, discomfort or mood disturbances(17) which can all contribute to sleep disturbance(18). Moreover, neurodegeneration of brain structures involved in the regulation of sleep and circadian rhythms, including the suprachiasmatic nucleus (SCN), likely mediate changes in sleep timing, sleep continuity, and sleep architecture in dementia(19). Dementia can therefore lead to impaired sleep initiation, reduced night-time sleep, difficulty maintaining sleep, increased night-time wandering, and excessive daytime sleepiness(2, 3, 20). Treating sleep problems may not only improve wellbeing, daytime functioning, and quality of life of those living with dementia but given the bi-directional relationship between sleep disruption and amyloid deposition and tau pathology(21) could potentially slow disease progression(22).

There is no conclusive evidence that any therapy to treat sleep disturbance in dementia is effective(11, 15, 23). A Cochrane review of pharmacotherapies found no conclusive randomised controlled trial (RCT) evidence for people living with dementia(24). Antipsychotics and benzodiazepines have adverse effects(25) and increase mortality in older adults(26). Pharmacological interventions, including melatonin, are not recommended as treatments(27) and

For peer review only

patients and their doctors prefer non-drug approaches for sleep problems(28). Cochrane reviews have found insufficient evidence for light therapy alone in dementia(15, 29). A recent Cochrane review of non-pharmacological interventions for sleep disturbance in people living with dementia found no conclusive evidence from the 19 included RCTs with none of the included studies identified to be at low risk of bias(15). This review found some positive effects for carer focused interventions and for interventions which promoted physical and social activities. Additionally, only four of the included studies recruited people living at home in the community, with most conducted in nursing homes which highlights a gap in good quality evidence conducted with people living with dementia in their own homes. As sleep disturbances in people living with dementia have mixed causes, promising interventions are likely to be multi-component. Wilfling et al conclude that multimodal and complex interventions have the strongest potential to improve sleep disturbance in people living with dementia(15). Although there is no conclusive evidence of any effective non-pharmacological treatment for sleep disturbances in people living with dementia(30), two pilot studies in community-dwelling people living with dementia, and our DREAMS START (Dementia RElAted Manual for Sleep; STrAtegies for RelaTives) feasibility RCT, found potential benefits of combining light with other components, including sleep education and hygiene, exercise, daytime activities and cognitive behavioural therapy (CBT) (31-33). We need clinically and cost-effective ways to improve disrupted sleep for people living with dementia, their families, health and social care systems, society and global economies.

This research builds on our successful feasibility RCT (NIHR/HTA 14/220/06)(31, 34). Our vision was to synthesise and add to incomplete evidence, co-produce a multi-component non-pharmacological intervention, and deliver it individually, tailoring it to fit each participant's needs. We co-produced DREAMS START using the best available evidence, patient and public involvement (PPI) and our clinical and research expertise in sleep and dementia(35). We incorporated the different components, including light, increased activity and exercise and carer's support which show promise in improving outcomes for people living with dementia and sleep disturbance(15) The intervention is delivered to family carers, who implement strategies to reduce the person with dementia's sleep disturbances. It uses natural daylight (where feasible) and if necessary, timed phototherapy to strengthen and stabilise sleep-wake timing. Additionally, it alerts carers to consider pain, uses strategies to increase comfort, reduce anxiety, increase daytime activity and uses CBT for sleep management, which Cochrane reviews found effective for older adults and family carers of people without dementia(36, 37). Our feasibility RCT found that the study design and the intervention were feasible and acceptable; 63/95 (65%; 95% CI 55% to 75%) eligible referrals consented, 62/95 (65%;

For peer review only

95% CI 55% to 75%) were randomised, and 37/42 (88%; 95% CI 75% to 96%) randomised to the intervention adhered to it. Qualitative interviews indicated that it was acceptable.

Study objectives

This study aims to determine whether our manualised, multi-component, non-pharmacological treatment package delivered by supervised non-clinically qualified psychology graduates, will deliver significant benefits for people living with dementia and their family carers. Additional aims are to assess the process and fidelity of delivery of the intervention and explore the experiences of family carers receiving the intervention. We will use the data generated to make evidence-based clinical practice recommendations on managing sleep disturbance in dementia.

Primary objective: To determine whether the DREAMS START intervention improves sleep disturbance in people living with dementia at home at 8 months compared to usual NHS treatment.

***Secondary objectives*:** To determine:

1. Whether the DREAMS START intervention improves sleep disturbance in people living with dementia at home at 4-months.
2. Whether it reduces daytime sleepiness (at 4 and 8 months).
3. Whether it improves people living with dementia's quality of life (at 4 and 8 months).
4. The probability that it is cost-effective compared to usual NHS treatment in regard to costs and preference based health related quality of life
5. The role of psychotropic medication and melatonin in any change.
6. Whether it improves family carer quality of life (at 4 and 8 months).
7. Whether it improves family carer's sleep and decreases their affective symptoms and burden (at 4 and 8 months).
8. The mechanisms of change.
9. If effective, how we can optimise the intervention for implementation at scale in the NHS.

METHODS AND ANALYSIS

Trial design

This is a multi-centre, parallel-group, superiority randomised controlled trial (RCT) with masked outcome assessment to test whether the DREAMS START intervention improves sleep disturbance in people living with dementia compared to usual treatment. Participants will be enrolled in the trial for eight months, assessments will take place at baseline, four months, and eight months post-randomisation (see Figure 1 for trial flow diagram). The trial is registered with the ISRCTN and an

For peer review only

independent trial steering committee (TSC) and data monitoring and ethics committee (DMEC) provide oversight.

Participants and recruitment

Potential participant dyads (family carers and people living with dementia) will be recruited from memory services and older adult mental health services in NHS Trusts in London, Essex, Sussex, and Tees, Esk and Wear Valleys and the Join Dementia Research (JDR) website. JDR is a free, secure online and telephone service developed and launched in 2014/2015 by the National Institute of Health and Care Research (NIHR).

Eligibility will be checked by researchers during initial screening calls to potential participants. People will be included in the study if they meet the following criteria:

1. People living with dementia (any type/severity/on any or no medication), except those currently drinking alcohol heavily.
2. Sleep disorders inventory (SDI) score ≥4. The SDI is a valid and reliable standalone tool for sleep disorder in people with dementia(38). Those who score ≥4 have clinically significant sleep disturbance.
3. Sleep that patient or their family judge as problematic. This is a pragmatic study and if the patient and family are unconcerned, treatment is unnecessary, as is the case in clinical practice. We asked the referrers at the point of entry into the study and assessors asked the family carers and person living with dementia again as part of eligibility criteria by during screening calls and initial assessment meetings.
4. Patient with capacity gives consent, or if not capacitous, consultee gives consent with patient not unwilling.
5. Family carer gives informed consent.
6. Family carer supports the person with dementia emotionally or practically at least weekly.
7. Person with dementia lives in their own home at the beginning of the study with someone present at night.

Exclusion criteria (any):

1. Known primary sleep breathing disorder diagnosis preceding dementia (e.g. sleep apnoea).
2. Current known heavy alcohol use (Alcohol Use Disorders Identification Test – Consumption (AUDIT C) Score ≥8). We have this criterion as during our earlier feasibility trial two participants drank alcohol during the intervention sessions during the day. These people were unable to work with a plan to change their sleep, which involved reducing alcohol, and we

For peer review only

were concerned for the safety of our facilitators visiting their homes alone. The participants dropped out.

3. People unavailable for >3 weeks (e.g. planned holiday or hospital admission) of intervention and follow-up.
4. Currently enrolled in another non-pharmacological dementia RCT.

If potential participants meet eligibility criteria, researchers will offer either a baseline home visit or if necessary, an appointment via telephone or video call. At this appointment, if potential participants are willing, researchers will obtain written informed consent (see Appendix 1) or where necessary (audio-recorded) verbal consent to take part in the study from family carers and people living with dementia who have capacity (or a personal consultee will sign for people living with dementia who lack capacity to consent). They will then complete the baseline assessments (see Assessments below, and Supplementary Table 1).

Intervention

DREAMS START is a six-session manual-based intervention for carers of people living with dementia to make changes to improve their relatives' sleep(31, 34). People living with dementia can join their carers in sessions if they wish. (see Appendix 2 for a summary of intervention components). The intervention provides information about sleep and dementia, supports carers to use practical *zeitgebers* (cues that influence the person's biological rhythms e.g. regular timing of bed and rising, morning wake-up light, regular mealtimes) and to establish adaptive stimulus control (e.g. pre-bed settling routine, management of wakeful episodes). It uses strategies to promote de-arousal at night (e.g. relaxation, bedroom comfort, no caffeine or alcohol pre-bed, relaxation) and daytime behavioural activation to maintain alertness and reduce daytime naps. The intervention also focuses on helping carers to look after their own (sleep) health, incorporating aspects of CBT. We use actigraphic data from devices worn by participants with dementia at baseline to inform the personalisation of intervention strategies. Over the sessions, carers will build a personalised action plan to continue using after the sessions are completed. We will aim to deliver the sessions weekly or fortnightly depending on the availability of the carer but will be flexible to maximise adherence. We will deliver either in person, online via video call or by telephone depending on the preferences of the carer.

Training and supervising facilitators: DREAMS START Facilitators will be non-clinically qualified psychology graduates who have participated in a two-day training programme delivered by the study team online. We have successfully adopted this approach in previous trials(39-41). This level of

For peer review only - http://bmjopen.bmj.com/site/about/guidelines.xhtml

expertise ensures the breadth of necessary skills (to impart information, knowledge of dementia, mental health and knowing when to ask for help). Our team has extensive experience in training psychology graduates and psychology assistants in memory and mental health services to deliver non-pharmacological interventions. DREAMS START was designed to be delivered by this same group, to maximise the potential for delivery at scale and at low cost (compared to less available and more expensive qualified psychologists or nurses delivering the intervention) and is compatible with a range of commissioning models. Training will focus on dementia and sleep-wake regulation, empathic listening skills, facilitating behaviour change, interpreting actigraphy, using supervision effectively and working collaboratively with family carers and people living with dementia. Facilitators will receive fortnightly group supervision with a clinical psychologist with additional individual supervision as requested by facilitators or the study team. We used this model in our feasibility RCT and found that the intervention was delivered to a high level of fidelity and was acceptable to and the facilitators positively experienced by participants.

Usual treatment: We are comparing the new treatment with treatment as usual (TAU). Participants randomised to the new treatment will also receive usual care. TAU varies according to where the person with dementia is treated and their individual needs, but incorporates National Institute for Health and Care Excellence (NICE) guidelines for dementia and consists of assessment, diagnosis, symptomatic interventions, risk assessment and management, advice and information(27). There is currently no consistent approach to the treatment of sleep difficulties in dementia. We expect usual treatment to reflect that identified during our feasibility trial, where interventions were medical, psychological and social(31). During our feasibility trial, 45% of participants were prescribed one or more psychotropic medications, with 11% prescribed anxiolytic or hypnotic medication. We will not exclude those taking medication for sleep but will note psychotropic medication prescribed and taken.

Randomisation, allocation concealment and blinding

The Trial Manager will perform the randomisation procedure, after consent and baseline data collection are completed. If the Trial Manager is not available, randomisation will be by a member of the research team who is not involved in participant recruitment or follow-up. Randomisation will be provided by a web based system using the company Sealed Envelope. Randomisation will occur at the level of the patient and will be blocked and stratified by site using a 1:1 intervention: treatment as usual ratio. Participants will be assigned to treatment groups through consecutive allocation of participant numbers and the use of a Trial Participant Log. The Trial Manager will notify the intervention facilitators of allocation, who will either arrange the intervention sessions or inform the

For peer review only

participant that they have been randomised to the control arm of the trial. Researchers collecting data will be masked to group allocations. The person facilitating the intervention will be different from the researcher conducting the follow-up assessments, if an individual has both roles this will not be in the same research sites. Clinical supervision will be conducted in separate groups based on different research sites to avoid unmasking of intervention facilitators who may also be collecting research data in a different site to that they are delivering in.

Participants and those delivering the intervention will be unmasked and aware of intervention status, which is common in behavioural and psychological intervention trials(42). As follow up assessments will be masked, there is a small risk that assessors may become unmasked accidentally by the participant or carer. We will minimise this risk by the following: assessors will remind participants at each stage that they must not discuss their intervention with their assessor and remind participants to hide any study related materials or equipment. If an assessor does become unmasked, we will make a note of this and ask an alternative assessor to complete future outcome measures for this participant. We have successfully adopted these procedures in previous trials of non-pharmacological interventions(31, 40, 43).

Assessments

The primary outcome is sleep disturbance at 8 months and will be measured using the SDI. The SDI is validated for measuring sleep disturbance in people living with dementia and describes the frequency and severity of sleep-disturbed behaviours. It is validated against clinical variables and used in recent promising pilot studies of non-pharmacological interventions(31-33, 44). The SDI will be collected at baseline, 4 months, and 8 months post-randomisation. We originally envisaged the primary outcome in this study would be sleep inferred through actigraphy, which fits with chosen primary outcomes in the recent Cochrane review of non-pharmacological interventions for sleep disturbance in dementia (15), However, the feasibility study indicated its unsuitability. Actigraphy is not validated in people with dementia and infers sleep from lack of movement. Many carers in our feasibility study informed us it was inaccurate e.g. one participant lay still in bed and screamed at night and actigraph output indicated excellent sleep. Others had movement disorders which are common in some dementias and at times did not lie still although they and their family thought they were asleep. In addition, markers of going to bed and getting up, which are necessary for actigraphy data interpretation (for at least seven days; the "gold" standard), were only available for 68% of people randomised in our earlier study(45). Conversely, in our feasibility RCT, the SDI was completed at follow-up by 92% of those recruited. This appeared the best way to measure sleep disturbance,

For peer review only

and family members in the trial and PPI said that they found it relevant and reflected their difficulties. All secondary outcome measures will be taken at baseline, 4 months, and 8 months (see Supplementary Table 1).

Secondary outcomes for the person living with dementia are all proxy measures to minimise burden. These include neuropsychiatric symptoms (Neuropsychiatric Inventory (NPI))(46), daytime sleepiness (Epworth sleepiness scale (ESS))(47), and quality of life (DEMQOL-Proxy)(48). The DEMQOL-Proxy will also be used in the cost-effectiveness analysis to calculate Quality of life Adjusted Life Years (QALY). A Modified Client Service Receipt Inventory (CSRI)(49) and EQ-5D 5 level (EQ-5D-5L)(50) proxy will be collected to inform the cost-effectiveness analysis and we will also collect details of medication including side effects. We will collect one week of actigraphy for the person living with dementia (using the Axivity AX3)(51)) from baseline and at 4 and 8-month follow-ups) to inform understandings of the mechanism of change of the intervention. The AX3 is an actigraph, a small, non-invasive device that is worn on the wrist like a watch that measures movement. Typically sleep is inferred from this data from lack of movement. We used actigraphs during our feasibility DREAMS START study and found that they were acceptable to many people living with dementia. We will use the data collected in our process evaluation, exploring changes in activity and how this relates to other outcomes. For family carers, we will assess sleep disturbance (Sleep Condition Indicator (SCI))(52), carer anxiety and depression (Hospital Anxiety and Depression Scale (HADS))(53), carer burden (Zarit Burden Interview (ZBI))(54) and health related quality of life (Health Status Questionnaire (HSQ-12))(55). To inform the cost-effectiveness analysis a Modified CSRI(49) incorporating the Valuation of Informal Care Questionnaire (iVICQ) a measure of carer time and activity and the Brief Work Productivity and Activity Impairment (WPAI) a measure of productivity loss as well as the EQ-5D-5L(50) will be collected at each time point.

Sample size calculation

We used the standard deviation (SD) of baseline SDI scores (15.74) and the correlation between baseline and follow-up measurements (0.57) observed in our feasibility trial for the power calculation(31). There is no published SDI minimum clinically important difference. We aim to detect a difference of ≥5.5 points, consistent with important differences identified through consultation with experts. This corresponds to a small to medium effect size of 0.35 and is realistic (an average difference of 5.6 was observed in feasibility work). To account for potential facilitator clustering in the intervention arm, we assumed an intracluster correlation coefficient (ICC) as observed in earlier studies(40, 43) (0.03) and approximately 15 participants per facilitator. To achieve equal allocation,

For peer review only

the initial calculation, before adjustment for clustering, was based on unequal allocation (1:0.75) which, after inflation for clustering in the intervention arm resulted in 1:1 allocation.

Based on these assumptions, to detect a difference in average SDI of 5.5 between intervention and TAU with power 90% and 5% significance requires 370 participants; 185 in each arm. This calculation allows for up to 15% drop out and includes an inflation for potential non-normality of SDI (as observed in feasibility work)(56). Calculation of sample size was carried out using STATA version 14.

Adverse events

All adverse events (except those listed below as expected) will be recorded in the medical records/case notes/source data in the first instance and will be recorded with clinical symptoms and accompanied by a simple, brief description of the event, including dates as appropriate. All adverse events will be recorded until the participant has completed the intervention.

All serious adverse events (except those listed below as expected) will be recorded in the case report form (CRF) and SAE log. This is because the intervention is not invasive, already has a well-known safety profile and therefore, the collection of AE data will not add any value to the safety profile of the intervention. We will collect information on any planned hospitalization at baseline and these will not be considered adverse events. Based on our feasibility work we expect few side effects specific to the intervention. The events listed below describe expected procedural/disease-related AEs/SAEs:

- Emotional distress or discomfort as a result of the questionnaires or intervention.
- Falls as a result of increased physical activity or getting out and about more or because of other age-related conditions.
- Death, hospitalisation due to common causes related to dementia (e.g. UTIs, delirium, common infections) or transition to a care home as a result of the progression of illness.

Analysis plan

Statistical analysis

For each randomised group we will summarise the primary outcome (SDI at 8-month) using means with standard deviations and medians with interquartile ranges. We will also graphically examine the distribution of the score and average scores over time. We will describe the effect of the intervention using the difference in mean SDI score calculated with a 95% confidence interval. This estimate will be obtained from a 3 level linear mixed effects regression model which has random effects to allow for repeated outcome measurements at 4 and 8 months and for clustering by

For peer review only - http://bmjopen.bmj.com/site/about/guidelines.xhtml

facilitator in the intervention arm (57). The model will include as fixed effects a treatment group indicator, baseline SDI score, study site, a time indicator and an interaction between treatment group and time. If assumptions of the regression model are violated, the SDI score will be analysed after appropriate transformation (e.g. a log transformation). We will calculate the intra-cluster correlation coefficient (with 95% confidence interval) to describe facilitator clustering.

We will carry out all analyses by intention to treat (ITT), comparing the groups as randomised. Analyses of secondary clinical outcomes will take a similar approach to the analysis of the primary outcome. Analyses will be based on people with available outcome data at 4 or 8 months and will rely on an assumption that data is missing at random. We will describe the number (%) of participants with missing outcome in each group, look at reasons for missingness and consider characteristics of the patients excluded from the ITT analysis. In a sensitivity analysis, we will re-estimate the treatment effect with additional adjustment for baseline predictors of missingness. Further analyses based on multiple imputation methods will include use of pattern mixture models under pre-defined missing not at random scenarios(58).

A separate statistical analysis plan (available to download from https://doi.org/10.1186/ISRCTN13072268) will predefine the detail of all intended analyses.

Health economic analysis

We will calculate the incremental cost per QALY gained of DREAMS START plus TAU compared to TAU only, over 8 months from a health and social care cost perspective using the EQ-5D-5L proxy to calculate QALYs in line with NICE guidance. Secondary analyses will calculate QALYs using the DEMQOL and relevant tariff and include impact and cost of carers (paid as well as family and close others) out of pocket costs and relevant wider societal costs.

We will include the EQ-5D-5L (a generic measure of health-related quality of life) for carers in the economic evaluation. We will not measure carer productivity loss alone, some carers will be retired, so measuring productivity loss, which focuses on absenteeism from paid employment and presenteeism within paid employment, is not sufficient here. Instead, we will capture the equivalent of “productivity loss” in our carer population by measuring days off and changes in employment such as moving from full-time to part-time employment and retirement. This will be costed using the human capital approach. The most important component of this analysis will be the impact on carer time and healthcare resource use which will be measured using the Valuation of Informal Care Questionnaire and a modified CSRI respectively. We will also include the brief Work Productivity and

For peer review only

Activity Impairment (WPAI) questionnaire for caregivers, to ensure we fully capture productivity losses in carers that are in employment.

We will use the number of sessions attended, duration of the session and grade of the staff delivering the session to calculate participant-level costs of DREAMS START, in addition to the training and supervision cost. We modified the CSRI based on data from our feasibility study and will use costs from published sources to cost healthcare resource use.

Carers will complete the questionnaires about resource use, time spent caring and productivity loss in the past 4 months at baseline, 4-, and 8 -month. We will calculate QALYs as the area under the curve, adjusting for baseline, over 8 months using the EQ-5D-5L and DEMQOL-Proxy to calculate QALYs. We will report descriptive statistics for resource use, costs and QALYs for baseline, 4 and 8 -month. We will calculate the mean incremental costs and QALYs of DREAMS START compared to usual care over 8 months using patient-level data linear regression, adjusting for site and facilitator clustering in line with the statistical analysis plan, with 95% confidence intervals, cost-effectiveness planes and cost-effectiveness acceptability curves calculated using bootstrapping. We will utilize the "net monetary benefit approach" to construct cost-effectiveness acceptability curves. On top of this, we will report the probability that the intervention is cost-effective for a range of decision thresholds, ranging from £0 to £100,000. We will conduct sensitivity analyses for any assumptions made. We will assess the level and type of missing data and in conjunction with the statistician determine the most appropriate method for accounting for missing data, which is likely to be multiple imputation using chained equations.

The full Health Economics Analysis Plan (HEAP) is available to download from https://doi.org/10.1186/ISRCTN13072268.

Process evaluation

In line with the updated MRC framework for developing and evaluating complex interventions(59) we will conduct a process evaluation to explore how the intervention works and in what contexts, this will be informed by our draft logic model (see Figure 2) which prespecifies how we expect the intervention to work and what factors may contribute to this.

Qualitative analysis

We will purposively sample and conduct qualitative interviews with 15-20 participants (selected for maximum variation, men and women, completers and non-completers, spouses and non-spouse

For peer review only

family carers and people living with dementia, across sites) and 5 staff delivering the intervention. We will ask about their experiences of different elements of the intervention including the content, form and process of delivery, barriers and facilitators to implementing strategies and perceived impact of the intervention for carers and people living with dementia. We will transcribe qualitative data verbatim, anonymise the transcripts and use NVivo 12. We will use a thematic analytic approach(60) informed by our draft logic model and the Theoretical Domains Framework (61). The team will organise data into preliminary themes iteratively identifying similarities and differences in the data. We will triangulate qualitative data with fidelity ratings, intervention adherence (session attendance; action plans, strategy tried) and quantitative outcomes (including patterns in activity from actigraphy).

Quantitative analysis

In considering change mechanisms, in line with our draft logic model, the analysis will focus on movement as a potentially important indicator. The intervention incorporates increasing daytime activity and reducing napping to increase pressure to fall asleep at night for the person with dementia as well as strengthening the relationship between bed and sleep at night-time and using light to reinforce circadian rhythmicity. As such we would expect increased movement during the day and decreased movement at night which we will explore via actigraphy. We will consider rest-activity amplitude, reflecting the relative difference between the least active five hours (L5) and the most active ten hours (M10) in the day. These will be recorded via the actigraphs worn for a week at baseline and for a week at the 4 and 8--month follow-up points. The mediating effect of these measures on our primary and secondary outcomes will be examined using the stepped approach described by Whittle et al (62). Models fitted in all steps will be mixed models that account for facilitator clustering.

Fidelity analysis

To analyse the fidelity of delivery of DREAMS START, we will assess the number of appointments delivered across all intervention participants. Checklists will be applied independently of the facilitator to a random selection of one recorded intervention session for each participant. A mean fidelity score will be produced by dividing the number of items on the checklist identified as being delivered, by the number of items on the checklist that should have been delivered per appointment, per researcher and across all appointments. We will adopt thresholds used in other intervention fidelity work: where 81–100% constitutes high fidelity, 51-80% is moderate fidelity and 50% or lower constitutes low fidelity(63).

For peer review only

Patient and public involvement

We are partnered by Alzheimer's Society and their research network volunteers, including our co-applicant RH, who has experience as a carer and will continue her input, having contributed to our earlier feasibility study and development of the grant application for this RCT. We have a virtual PPI group and PPI members on our TSC and Trial Management Group (TMG), to provide partnership and enhance the relevance, practicality and utility of our materials and findings. They have advised on the language and content of information sheets, contributed to the revision of the intervention having been integral to the initial intervention co-production and are working with us on knowledge exchange and dissemination activities.

STRENGTHS AND LIMITATIONS

Few trials have tested interventions for sleep in dementia wholly with those living at home and this will be the largest RCT to date testing a multi-component, non-pharmacological intervention with those living with dementia and sleep disturbance. There are a number of potential limitations to our trial protocol. We have excluded those living alone without someone present at night which reduces the generalizability of our findings to the excluded group, who often have high levels of need and potential risk. This decision was based upon our earlier feasibility trial when we included those living alone. When no one else was there at night, carers (family or paid) were unable to implement strategies, like, a scheduled bedtime or wind down routine. It was also difficult to gain reliable information about the sleep patterns of people with dementia living alone and when the intervention was delivered to people living with dementia alone this was often confusing for them. We have also noted above that we could not conduct a double-blind trial of the DREAMS START intervention which potentially increases the risk of performance bias.

ETHICS AND DISSEMINATION

London – Camden & Kings Cross Research Ethics Committee (Reference: 20/LO/0894) approved the study on 18th September 2020.

We will disseminate our findings in high-impact peer-reviewed journals and at national and international conferences. We will present findings in appropriate local forums for health and social care professionals; participants who have indicated they are interested in the results will be sent a lay summary. We are working with an extensive network of academic researchers, policymakers, stakeholders and PPI, acting as a 'Community of Interest' to advise on and ensure acceptability for the dissemination of the research, and to ensure both national and international reach. We aim to

For peer review only

produce an implementation toolkit alongside our DREAMS START intervention and facilitator training materials.

This proposed research has the potential to improve sleep and quality of life for people living with dementia and their family carers, in a feasible and scalable intervention, without medication side effects, and to elucidate the mechanisms of impact. If clinically effective, our intervention should be cost-effective: it is cheap and potentially will reduce the health and social care burden, particularly if it delays care home admission.

TRIAL STATUS

The trial commenced recruitment in February 2021 and completed recruitment in March 2023 with final primary outcome data collected by November 2023 and the final report will be submitted in May 2024.

Author's contributions

All authors made a substantial contribution to this work. PR drafted the manuscript. All authors were involved in revising the manuscript, giving final approval of the version to be published and agree to be accountable for all aspects of the work. GL and PR conceived of the study and acquired funding. GL, PR, JB, RHu, CCl, SB, MR, GC, ZW, LW, CE, SDK, RHo, SA, MA, LG, MM, SM, ET, CCo and LP contributed to developing the protocol and its implementation.

Funding Statement

This study/project is funded by the National Institute for Health and Care Research (NIHR) Health Technology Assessment Programme (NIHR HTA 128761). The views expressed are those of the author(s) and not necessarily those of the NIHR or the Department of Health and Social Care. SDK and CAE are supported by the NIHR Oxford Health Biomedical Research Centre.

Competing interests

The present manuscript is supported by a National Institute for Health and Care Research (NIHR) Health Technology Assessment Programme (HTA) grant (NIHR HTA 128761). SB declares grants from NIHR, CIHR, ESRC, HEE, ESPRC, Alzheimer's Society, and the Alzheimer's Association with no COI with current work. CE declares grants from NIHR-HTA, NIHR-EME, NIHR-BRC, Wellcome Trust with no COI with current work. MR declares a grant from NIHR ARC KSS with no COI with current work. Outside the submitted work SK declares non-financial support from Big Health Ltd. in the form of no cost access to the digital sleep improvement programme, Sleepio, for use in clinical research. Outside the

For peer review only

submitted work CE declares stock/stock options and other salary contributions from Big Health Limited developers of Sleepio. Outside the submitted work SSB declares personal fees and non-financial support from Lilly, personal fees from Boehringer-Ingelheim, personal fees from Axovant, personal fees from Lundbeck, personal fees from Nutricia and honoraria from the Hamad Medical Service for lectures and talks. Outside the submitted work MR declares Honorarium for presentation on Lewy body dementias for GE. SB is a trustee of the Alzheimer's Society and NED at the Somerset NHS Foundation Trust. CE is deputy editor of the Journal of Sleep Research and on the editorial board of Sleep Medicine reviews.

Data Sharing statement

The qualitative and quantitative datasets generated during and/or analysed during the current study will be available upon request from PRIMENT CTU Data Management Group on priment@ucl.ac.uk in collaboration with members of DREAMS START Trial Team. Any request for data must come through to PRIMENT CTU in the first instance and where the request is reasonable, anonymised datasets, stored on the publicly available UCL Research Data Repository https://rdr.ucl.ac.uk/ will be shared once analysis and publication of the trial findings has occurred.

For peer review only

REFERENCES

1. Prince M, Wimo A, Guerchet M, Ali G, Wu Y, Prina M. World Alzheimer Report 2015. The global impact of dementia. An analysis of prevalence, incidence, cost & trends; Alzheimer's Disease International: London. 2015.
2. Dauvilliers Y. Insomnia in patients with neurodegenerative conditions. Sleep Medicine. 2007;8:S27-S34.
3. Moran M, Lynch CA, Walsh C, Coen R, Coakley D, Lawlor BA. Sleep disturbance in mild to moderate Alzheimer's disease. Sleep Medicine. 2005;6(4):347-52.
4. de Oliveira AM, Radanovic M, de Mello PC, Buchain PC, Vizzotto AD, Celestino DL, et al. Nonpharmacological Interventions to Reduce Behavioral and Psychological Symptoms of Dementia: A Systematic Review. Biomed Res Int. 2015;2015:218980.
5. Ooms S, Ju Y-E. Treatment of sleep disorders in dementia. Current treatment options in neurology. 2016;18(9):40.
6. Koren T, Fisher E, Webster L, Livingston G, Rapaport P. Prevalence of sleep disturbances in people with dementia living in the community: A systematic review and meta-analysis. Ageing Research Reviews. 2022:101782.
7. Kyle SD, Espie CA, Morgan K. "… Not just a minor thing, it is something major, which stops you from functioning daily": quality of life and daytime functioning in insomnia. Behavioral sleep medicine. 2010;8(3):123-40.
8. Ju Y-ES, Lucey BP, Holtzman DM. Sleep and Alzheimer disease pathology—a bidirectional relationship. Nature reviews Neurology. 2014;10(2):115.
9. Prince M, Knapp M, Guerchet M, McCrone P, Prina M, Comas-Herrera A, et al. Dementia UK: update. London: Alzheimer's Society; 2014.
10. Gehrman P, Gooneratne NS, Brewster GS, Richards KC, Karlawish J. Impact of Alzheimer disease patients' sleep disturbances on their caregivers. Geriatric Nursing. 2018;39(1):60-5.
11. Kinnunen KM, Vikhanova A, Livingston G. The management of sleep disorders in dementia: an update. Curr Opin Psychiatry. 2017;30(6):491-7.
12. Gehrman P, Gooneratne NS, Brewster GS, Richards KC, Karlawish J. Impact of Alzheimer disease patients' sleep disturbances on their caregivers. Geriatric Nursing. 2017.
13. McCurry SM, Logsdon RG, Teri L, Vitiello MV. Sleep disturbances in caregivers of persons with dementia: contributing factors and treatment implications. Sleep medicine reviews. 2007;11(2):143-53.
14. McCrae CS, Dzierzewski JM, McNamara JP, Vatthauer KE, Roth AJ, Rowe MA. Changes in sleep predict changes in affect in older caregivers of individuals with Alzheimer's dementia: a multilevel model approach. Journals of Gerontology Series B: Psychological Sciences and Social Sciences. 2014;71(3):458-62.
15. Wilfling D, Calo S, Dichter MN, Meyer G, Möhler R, Köpke S. Non-pharmacological interventions for sleep disturbances in people with dementia. Cochrane Database of Systematic Reviews. 2023(1).
16. Webster L, Powell K, Costafreda SG, Livingston G. The impact of sleep disturbances on care home residents with dementia: the SIESTA qualitative study. International Psychogeriatrics. 2020;32(7):839-47.
17. Browne J, Edwards DA, Rhodes KM, Brimicombe DJ, Payne RA. Association of comorbidity and health service usage among patients with dementia in the UK: a population-based study. BMJ open. 2017;7(3):e012546.
18. Parliamentary Office of Science and Technology (POST). POSTbrief 29: Sleep and Long-Term Health. UK: Houses of Parliament; 2018.
19. Wang C, Holtzman DM. Bidirectional relationship between sleep and Alzheimer's disease: role of amyloid, tau, and other factors. Neuropsychopharmacology. 2020;45(1):104-20.

For peer review only

20. Cagnin A, Fragiacomo F, Camporese G, Turco M, Bussè C, Ermani M, et al. Sleep-wake profile in dementia with Lewy bodies, Alzheimer's disease, and normal aging. Journal of Alzheimer's Disease. 2017;55(4):1529-36.
21. Mander BA, Winer JR, Jagust WJ, Walker MP. Sleep: A Novel Mechanistic Pathway, Biomarker, and Treatment Target in the Pathology of Alzheimer's Disease? Trends Neurosci. 2016;39(8):552-66.
22. Holth JK, Patel TK, Holtzman DM. Sleep in Alzheimer's Disease–beyond amyloid. Neurobiology of sleep and circadian rhythms. 2017;2:4-14.
23. Livingston G, Sommerlad A, Orgeta V, Costafreda SG, Huntley J, Ames D. Dementia prevention, intervention, and care. 2017;390:2673-734.
24. McCleery J, Sharpley AL. Pharmacotherapies for sleep disturbances in dementia. Cochrane Database of Systematic Reviews. 2020(11).
25. Berry SD, Placide SG, Mostofsky E, Zhang Y, Lipsitz LA, Mittleman MA, et al. Antipsychotic and benzodiazepine drug changes affect acute falls risk differently in the nursing home. Journals of Gerontology Series A: Biomedical Sciences and Medical Sciences. 2015;71(2):273-8.
26. Weich S, Pearce HL, Croft P, Singh S, Crome I, Bashford J, et al. Effect of anxiolytic and hypnotic drug prescriptions on mortality hazards: retrospective cohort study. Bmj. 2014;348:g1996.
27. National Institute for Health and Care Excellence (NICE). Dementia: assessment, management and support for people living with dementia and their carers. NICE guideline (NG97): Methods, evidence and recommendations. . 2018.
28. Dzierzewski JM, O'Brien EM, Kay D, McCrae CS. Tackling sleeplessness: psychological treatment options for insomnia in older adults. Nature and science of sleep. 2010;2:47.
29. Forbes D, Blake CM, Thiessen EJ, Peacock S, Hawranik P. Light therapy for improving cognition, activities of daily living, sleep, challenging behaviour, and psychiatric disturbances in dementia. The Cochrane Library. 2014.
30. Brown CA, Berry R, Tan MC, Khoshia A, Turlapati L, Swedlove F. A critique of the evidence base for non-pharmacological sleep interventions for persons with dementia. Dementia. 2013;12(2):210-37.
31. Livingston G, Barber JA, Kinnunen KM, Webster L, Kyle SD, Cooper C, et al. DREAMS-START (Dementia RElAted Manual for Sleep; STrAtegies for RelaTives) for people with dementia and sleep disturbances: a single-blind feasibility and acceptability randomized controlled trial. International psychogeriatrics. 2018:1-15.
32. Gibson RH, Gander PH, Dowell AC, Jones LM. Non-pharmacological interventions for managing dementia-related sleep problems within community dwelling pairs: A mixed-method approach. Dementia. 2017;16(8):967-84.
33. Tewary S, Cook N, Pandya N, McCurry SM. Pilot test of a six-week group delivery caregiver training program to reduce sleep disturbances among older adults with dementia (Innovative practice). Dementia. 2018;17(2):234-43.
34. Kinnunen KM, Rapaport P, Webster L, Barber J, Kyle SD, Hallam B, et al. A manual-based intervention for carers of people with dementia and sleep disturbances: an acceptability and feasibility RCT. Health Technology Assessment (Winchester, England). 2018;22(71):1-408.
35. Rapaport P, Webster L, Horsley R, Kyle SD, Kinnunen KM, Hallam B, et al. An intervention to improve sleep for people living with dementia: Reflections on the development and co-production of DREAMS:START (Dementia RElAted Manual for Sleep: STrAtegies for RelaTives). Dementia. 2018;17(8):976-89.
36. Montgomery P, Dennis JA. Cognitive behavioural interventions for sleep problems in adults aged 60+. The Cochrane Library. 2003.
37. Sivertsen B, Nordhus IH. Management of insomnia in older adults. The British Journal of Psychiatry. 2007;190(4):285-6.

For peer review only

38. Tractenberg RE, Singer CM, Cummings JL, Thal LJ. The Sleep Disorders Inventory: an instrument for studies of sleep disturbance in persons with Alzheimer's disease. Journal of sleep research. 2003;12(4):331-7.
39. Cape J, Leibowitz J, Whittington C, Espie C, Pilling S. Group cognitive behavioural treatment for insomnia in primary care: a randomized controlled trial. Psychological medicine. 2016;46(5):1015-25.
40. Livingston G, Barber J, Rapaport P, Knapp M, Griffin M, King D, et al. Clinical effectiveness of a manual based coping strategy programme (START, STrAtegies for RelaTives) in promoting the mental health of carers of family members with dementia: pragmatic randomised controlled trial. BMJ. 2013;347:f6276.
41. Livingston G, Barber J, Rapaport P, Knapp M, Griffin M, King D, et al. Long-term clinical and cost-effectiveness of psychological intervention for family carers of people with dementia: a single-blind, randomised, controlled trial. Lancet Psychiatry. 2014;1(7):539-48.
42. Juul S, Gluud C, Simonsen S, Frandsen FW, Kirsch I, Jakobsen JC. Blinding in randomised clinical trials of psychological interventions: a retrospective study of published trial reports. BMJ Evidence-Based Medicine. 2021;26(3):109-.
43. Livingston G, Barber J, Marston L, Stringer A, Panca M, Hunter R, et al. Clinical and cost-effectiveness of the Managing Agitation and Raising Quality of Life (MARQUE) intervention for agitation in people with dementia in care homes: a single-blind, cluster-randomised controlled trial. The Lancet Psychiatry. 2019.
44. McCurry SM, Pike KC, Vitiello MV, Logsdon RG, Larson EB, Teri L. Increasing walking and bright light exposure to improve sleep in community-dwelling persons with Alzheimer's Disease: results of a randomized, controlled trial. Journal of the American Geriatrics Society. 2011;59(8):1393-402.
45. Kinnunen K, P R, L W, J B, D KS, B H, et al. A manual-based intervention for carers of people with dementia and sleep disturbances: an acceptability and feasibility RCT. . Health Technol Assess. 2018;22(71).
46. Cummings JL. The Neuropsychiatric Inventory: assessing psychopathology in dementia patients. Neurology. 1997;48(5 Suppl 6):S10-6.
47. Johns MW. Sleepiness in different situations measured by the Epworth Sleepiness Scale. Sleep. 1994;17(8):703-10.
48. Smith SC, Lamping DL, Banerjee S, Harwood RH, Foley B, Smith P. Development of a new measure of health-related quality of life for people with dementia: DEMQOL. 2007;37:737-46.
49. Beecham J, Knapp M. Costing Psychiatric Interventions. In: Thornicroft G, Brewin CR, Wing J, editors. Measuring Mental Health Needs. London: Gaskell/Royal College of Psychiatrists; 1992.
50. Bryan S, Hardyman W, Bentham P, Buckley A, Laight A. Proxy completion of EQ-5D in patients with dementia. Quality of Life Research. 2005;14(1):107-18.
51. Clarke CL, Taylor J, Crighton LJ, Goodbrand JA, McMurdo ME, Witham MD. Validation of the AX3 triaxial accelerometer in older functionally impaired people. Aging clinical and experimental research. 2017;29(3):451-7.
52. Espie CA, Kyle SD, Hames P, Gardani M, Fleming L, Cape J. The Sleep Condition Indicator: a clinical screening tool to evaluate insomnia disorder. BMJ open. 2014;4(3):e004183.
53. Zigmond AS, Snaith RP. The hospital anxiety and depression scale. Acta psychiatrica scandinavica. 1983;67(6):361-70.
54. Zarit SH, Reever KE, Bach-Peterson J. Relatives of the impaired elderly: correlates of feelings of burden. Gerontologist. 1980;20(6):649-55.
55. Barry TL, Kaiser KL, Atwood JR. Reliability, validity, and scoring of the Health Status Questionnaire-12 version 2.0. J Nurs Meas. 2007;15(1):24-35.
56. Randles R, Wolfe D. Introduction to the theory of nonparametric statistics. : Krieger; 1991.

For peer review only

57. Walwyn R, Roberts C. Therapist variation within randomised trials of psychotherapy: implications for precision, internal and external validity. Statistical Methods in Medical Research. 2010;19(3):291-315.
58. Cro S, Morris TP, Kenward MG, Carpenter JR. Sensitivity analysis for clinical trials with missing continuous outcome data using controlled multiple imputation: A practical guide. Statistics in Medicine. 2020;39(21):2815-42.
59. Skivington K, Matthews L, Simpson SA, Craig P, Baird J, Blazeby JM, et al. A new framework for developing and evaluating complex interventions: update of Medical Research Council guidance. BMJ. 2021;374:n2061.
60. Braun V, Clarke V. Using thematic analysis in psychology. Qualitative Research in Psychology. 2006;3(2):77-101.
61. French SD, Green SE, O'Connor DA, McKenzie JE, Francis JJ, Michie S, et al. Developing theory-informed behaviour change interventions to implement evidence into practice: a systematic approach using the Theoretical Domains Framework. Implementation Science. 2012;7.
62. Whittle R, Mansell G, Jellema P, van der Windt D. Applying causal mediation methods to clinical trial data: What can we learn about why our interventions (don't) work? European Journal of Pain. 2017;21(4):614-22.
63. Noell GG, FM; Gansle, K. Does treatment integrity matter? A preliminary investigation of instructional implementation and mathematical performance. Journal of Behavioral Education 2002;11:15.

For peer review only

Figure Legends:

Figure 1 - DREAMS START RCT flow diagram

Figure 2 – Draft logic model

For peer review only

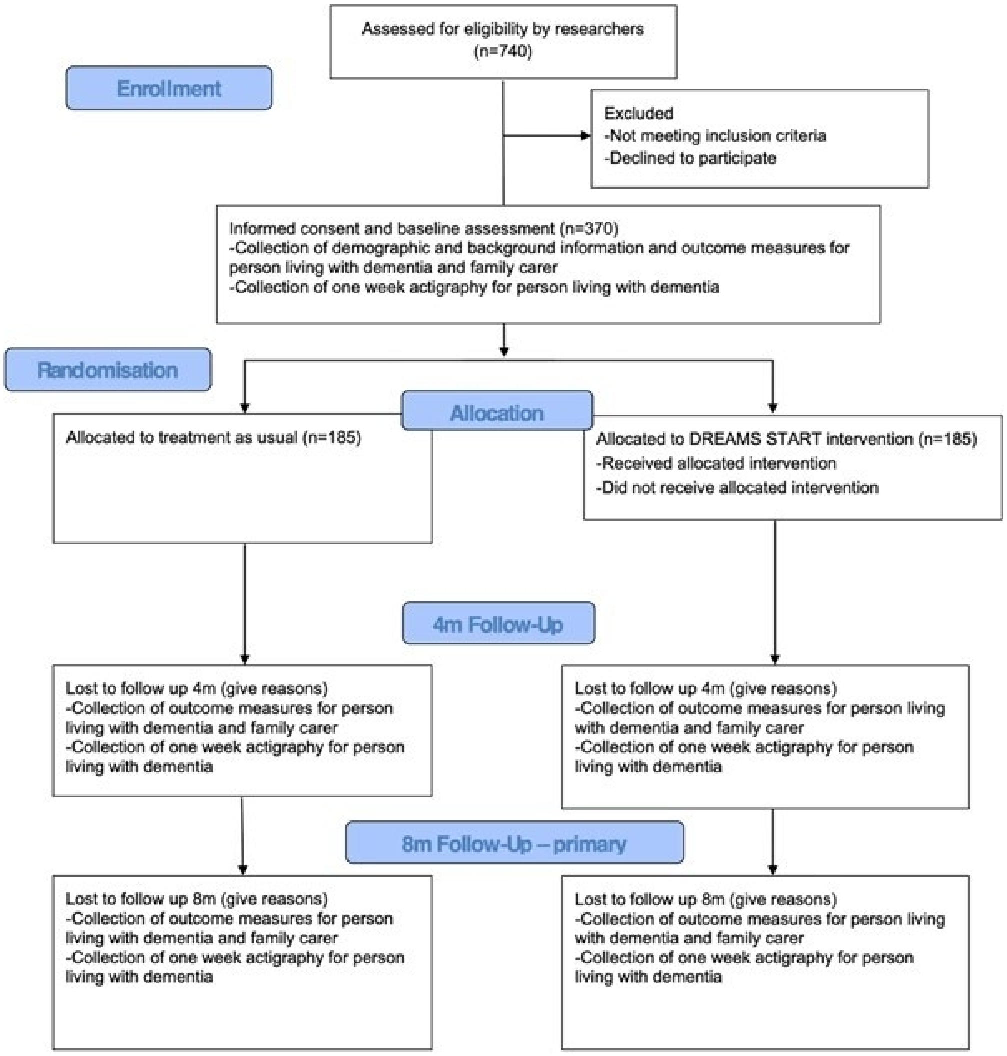

DREAMS START RCT flow diagram

50x56mm (300 x 300 DPI)

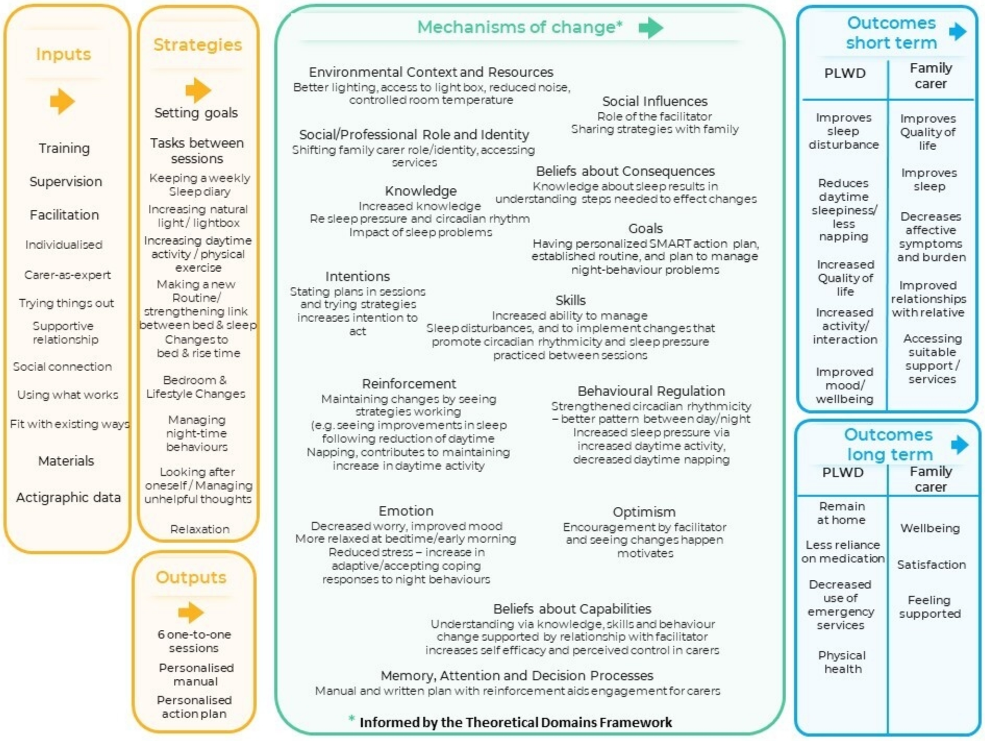

Draft logic model

81x60mm (300 x 300 DPI)

Supplementary Table 1 Schedule of visits and assessments

	Screening / Baseline	Randomisation	Session 1	Session 2	Session 3	Session 4	Session 5	Session 6	4mth follow up	8mth follow up	+ 8month
Informed Consent	x										
Sleep disorders inventory	x								x	x	
Eligibility confirmation	x										
Demographics (person with dementia)	x										
Neuropsychiatric Inventory	x								x	x	
Epworth Sleepiness Scale	x								x	x	
DEMQOL-Proxy	x								x	x	
CSRI/medication (proxy, for person with dementia)	x								x	x	
EQ-5D-5L (proxy, for person with dementia)	x								x	x	
Side effects	x								x	x	
Actigraphy	x								x	x	
Demographics (carer)	x										
Sleep Condition Indicator (carer)	x										

For peer review only

Hospital Anxiety and Depression Scale (carer)	x								x	x	
Zarit Burden Interview (carer)	x								x	x	
Health Status Questionnaire (12) (carer)	x								x	x	
CSRI/medication (carer)	x								x	x	
EQ-5D-5L (carer)	x								x	x	
Randomisation		x									
DREAMS START intervention			x	x	x	x	x	x			
Intervention acceptability (qualitative interview)											x

For peer review only

Appendix 1 Informed consent forms

**Site/Centre Number:**
**Dyad Number:**
**Participant Identification Number:**

[INSERT NHS TRUST LOGO HERE]

FAMILY CARER CONSENT FORM

Title: DREAMS START: Dementia-related manual for sleep strategies for relatives

Name of Researcher:

Please initial box

1. I confirm that I have read the information sheet dated 20-DEC-2020 (version 2.1) for the above study and understand the purpose of the research and I have received a copy of the information sheet and informed consent form to keep
2. I have had the opportunity to consider the information, ask questions and have had these answered satisfactorily
3. I understand that my participation is voluntary and that I am free to withdraw from the research study at any time without my legal rights or medical care being affected.
4. I understand that that my personal information will be processed only for the purposes of research and that all material and personal information will be kept in accordance with General Data Protection Regulation (GDPR) and the Data Protection Act 2018.
5. I understand that relevant data collected during the study may be looked at by individuals from the sponsor of the trial (Camden & Islington NHS Foundation Trust), UCL, regulatory authorities or from the NHS Trust, where it is relevant to my taking part in this research. I give permission for these individuals to have access to this data
6. I understand that a UCL-approved transcription company will have temporary access to the information I provide in the audio recordings and I agree for my sessions and interview with the researcher to be audiotaped *(Optional)*
7. I agree to be contacted again for future research studies *(Optional)*
8. I understand that the information I share, including anonymised direct quotes, may be included in any resulting report, publications and conference presentations *(Optional)*
9. I agree that the information collected about me can be used to support other research in the future, and may be shared anonymously with other researchers *(Optional)*
10. I would like to receive a summary of the research findings *(Optional)*
11. I agree to take part in the above study
12. I agree to share my mobile phone number to receive text reminders as part of the intervention and for my mobile number only to be transferred to a UCL-approved text messaging service

Name of Participant	Date	Signature
Name of Person taking consent	Date	Signature

DREAMS START FC_CONSENT_IRAS 272935_v2.1_20-DEC-2020 Page 1 of 1

Site/Centre Number:

Dyad Number:

Participant Identification Number:

[INSERT NHS TRUST LOGO HERE]

CONSULTEE DECLARATION FORM

Title: DREAMS START: Dementia-related manual for sleep strategies for relatives

Please initial box

1. I [insert name] have been consulted about [insert name of participant]'s participation in this research project. I confirm that I have read the information sheet dated 20-DEC-2020 (version 2.1) for the above study and understand the purpose of the research and I have received a copy of the information sheet and consultee declaration form to keep. ☐
2. I have had the opportunity to consider the information, ask questions and have had these answered satisfactorily. ☐
3. I understand that his/her participation is voluntary and that I am free to request that they are withdrawn from the study at any time without their legal or medical rights being affected. ☐
4. I understand that his/her personal information will be processed only for the purposes of research and that all material and personal information will be kept in accordance with General Data Protection Regulation (GDPR) and the Data Protection Act 2018. ☐
5. I understand that relevant data collected during the study may be looked at by individuals from the sponsor of the trial (Camden & Islington NHS Foundation Trust), UCL, regulatory authorities or from the NHS Trust, where it is relevant to their taking part in this research. I give permission for these individuals to have access to this data. ☐
6. I agree for his/her GP being informed of their participation in the study, including any necessary exchange of information about him/her between their GP and the research team ☐
7. I understand that an external UCL-approved transcription company will have temporary access to the information he/she provides in the audio recording and I agree for their sessions and interview with the researcher to be audiotaped. *(optional)* ☐
8. I agree to he/she being contacted again for future research studies *(optional)* ☐
9. I understand that the information he/shares, including anonymised direct quotes, may be included in any resulting report, publications and conference presentations *(optional)* ☐
10. I understand that the information collected regarding he/she will be used to support other research in the future, and may be shared anonymously with other researchers *(optional)* ☐
11. He/she would like to receive a summary of the research findings *(optional)* ☐
12. In my opinion he/she would have no objection to taking part in the above study. ☐

Name of Consultee	Relationship to participant	Date	Signature

Name of Person taking consent	Date	Signature

For peer review only - http://bmjopen.bmj.com/site/about/guidelines.xhtml

Site/Centre Number:

Dyad Number:

Participant Identification Number:

[INSERT TRUST LOGO HERE]

PERSON LIVING WITH DEMENTIA CONSENT FORM

Title: DREAMS START: Dementia-related manual for sleep strategies for relatives

Please initial box

1. I confirm that I have read the information sheet dated 20-DEC-2020 (version 2.1) for the above study and understand the purpose of the research and I have received a copy of the information sheet and consent form to keep.
2. I have had the opportunity to consider the information, ask questions and have had these answered satisfactorily
3. I understand that my participation is voluntary and that I am free to withdraw from the research study at any time without my legal rights or medical care being affected.
4. I agree that if I lose capacity to consent during the study, I would/I would not be willing for my relative to act as a personal legal representative and give consent on my behalf
5. I understand that that my personal information will be processed only for the purposes of research and that all material and personal information will be kept in accordance with General Data Protection Regulation (GDPR) and the Data Protection Act 2018.
6. I understand that relevant data collected during the study may be looked at by individuals from the sponsor of the trial (Camden & Islington NHS Foundation Trust), UCL, regulatory authorities or from the NHS Trust, where it is relevant to my taking part in this research. I give permission for these individuals to have access to this data.
7. I agree to my General Practitioner being informed of my participation in the study, including any necessary exchange of information about me between my GP and the research team
8. I understand that an external UCL-approved transcription company will have temporary access to the information I provide in the audio recordings I agree for my sessions and interview with the researcher to be audiotaped *(optional)*
9. I agree to be contacted again for future research studies *(optional)*
10. I understand that the information I share, including anonymised direct quotes, may be included in any resulting report, publications and conference presentations *(optional)*
11. I agree that the information collected about me can be used to support other research in the future, and may be shared anonymously with other researchers *(optional)*
12. I would like to receive a summary of the research findings *(optional)*
13. I agree to take part in the above study

Name of Participant	Date	Signature
Name of Person taking consent	Date	Signature

DREAMS START PLWD_CONSENT_IRAS 272935_v2.1_20-DEC-2020 Page 1 of 1

Appendix 2 Intervention components

Session/Section	Derived from
**Session 1**	
Sleep and dementia –	material provided by Simon D Kyle.
What is sleep? –	material provided by Simon D Kyle.
What causes sleep problems in dementia? –	written by Penny Rapaport, Simon D Kyle and Gill Livingston for DREAMS.
Making changes to improve sleep –	adapted from cognitive–behavioural therapy (CBT) work by Colin A Espie.
The impact of sleep problems on you and our relative	developed for DREAMS.
Managing the stress that sleep problems can bring –	adapted from START*.
Managing stress: the signal breath –	adapted from START.
Summary –	adapted from START.
Putting it into practice –	adapted from START.
**Session 2**	
Recap on understanding sleep and dementia.	
Establishing a good day and night routine	adapted from CBT work by Colin A Espie.
Your relative's sleep pattern –	developed for DREAMS.
Light and sleep –	material provided by Simon D Kyle.
Light, dementia and the body clock –	material provided by Simon D Kyle.
Making a light therapy plan –	developed for DREAMS.
Making a new sleep routine: your relative's plan –	developed for DREAMS based on work on sleep efficiency by Colin A Espie and Simon D Kyle.
Managing stress 2: focused breathing –	adapted from START.
Summary –	adapted from START.
Putting it into practice –	adapted from START.
**Session 3**	
Recap on making a plan.	
The importance of daytime activity and routine –	adapted from START.
Planning daytime activity –	adapted from START.

For peer review only

Sleep, exercise and physical activity –	developed for DREAMS by Penny Rapaport, Gill Livingston and Simon D Kyle.
Managing stress 3: guided imagery –	adapted from START.
Summary –	adapted from START.
Putting it into practice –	adapted from START.
Seated exercises visual guide –	from NHS Choices website.66
**Session 4**	
Recap on daytime activity and routine.	
Troubleshooting: putting plans into action –	developed for DREAMS.
Your relative's plan	developed for DREAMS.
Managing night-time behaviour problems –	adapted from MARQUE**/START.
Describing and investigating behaviours –	adapted from MARQUE/START.
Managing stress 4: stretching –	adapted from START.
Summary –	adapted from START.
Putting it into practice –	adapted from START.
**Session 5**	
Recap on night-time behaviour problems.	
Creating strategies for managing behaviours –	adapted from MARQUE.
Managing your own sleep –	developed for DREAMS.
Managing thoughts and feelings –	adapted from CBT work by Colin A Espie.
Challenging unhelpful thoughts and feelings –	adapted from START.
Making time for yourself	adapted from START.
Managing stress 5: guided imagery – ocean escape –	adapted from START.
Summary –	adapted from START.
Putting it into practice –	adapted from START.
**Session 6**	**Overall structure based on that developed in START and refined in MARQUE.**
Putting it all together.	
What works? Light, sleep and dementia –	written by Simon D Kyle for DREAMS.
What works? The importance of daytime activity –	adapted from START.

For peer review only

What works? Making a new sleep routine –	based on the sleep manual by Colin A Espie and Simon D Kyle.
What works? Making changes to improve sleep –	based on the sleep manual by Colin A Espie and Simon D Kyle.
What works? Managing night-time behaviours –	based on MARQUE/START.
What works? Challenging unhelpful thoughts and feelings –	based on START and CBT work by Colin A Espie.
What works? Relaxation –	adapted from START.
Keeping it going – developing an action plan –	developed for DREAMS.
Action plan for you and your relative –	developed for DREAMS.
Summary –	developed for DREAMS.

* STrAtegies for RelaTives (START) for further information see https://www.thelancet.com/journals/lanpsy/article/PIIS2215-0366(14)00073-X/fulltext

** Managing Agitation and Raising Quality of Life (MARQUE) for further information see https://pubmed.ncbi.nlm.nih.gov/30872010/

For peer review only

Reporting checklist for protocol of a clinical trial.

Based on the SPIRIT guidelines.

Instructions to authors

Complete this checklist by entering the page numbers from your manuscript where readers will find each of the items listed below.

Your article may not currently address all the items on the checklist. Please modify your text to include the missing information. If you are certain that an item does not apply, please write "n/a" and provide a short explanation.

Upload your completed checklist as an extra file when you submit to a journal.

In your methods section, say that you used the SPIRITreporting guidelines, and cite them as:

Chan A-W, Tetzlaff JM, Gøtzsche PC, Altman DG, Mann H, Berlin J, Dickersin K, Hróbjartsson A, Schulz KF, Parulekar WR, Krleža-Jerić K, Laupacis A, Moher D. SPIRIT 2013 Explanation and Elaboration: Guidance for protocols of clinical trials. BMJ. 2013;346:e7586

		Reporting Item	Page Number (in manuscript)	Page Number (in full protocol 13-JAN-2023 Version 4 available for download https://doi.org/10.1186/ISRCTN13072268)
**Administrative information**				

For peer review only

Title	#1	Descriptive title identifying the study design, population, interventions, and, if applicable, trial acronym	1	
Trial registration	#2a	Trial identifier and registry name. If not yet registered, name of intended registry	3	
Trial registration: data set	#2b	All items from the World Health Organization Trial Registration Data Set	18	
Protocol version	#3	Date and version identifier	13-JAN-2023 Version 4	
Funding	#4	Sources and types of financial, material, and other support	17	

For peer review only

Roles and responsibilities: contributorship	#5a	Names, affiliations, and roles of protocol contributors	17	
Roles and responsibilities: sponsor contact information	#5b	Name and contact information for the trial sponsor		1
Roles and responsibilities: sponsor and funder	#5c	Role of study sponsor and funders, if any, in study design; collection, management, analysis, and interpretation of data; writing of the report; and the decision to submit the report for publication, including whether they will have ultimate authority	n/a	

For peer review only

		over any of these activities		
Roles and responsibilities: committees	#5d	Composition, roles, and responsibilities of the coordinating centre, steering committee, endpoint adjudication committee, data management team, and other individuals or groups overseeing the trial, if applicable (see Item 21a for data monitoring committee)	7	50
**Introduction**				
Background and rationale	#6a	Description of research question and justification for	4, 5	

For peer review only

		undertaking the trial, including summary of relevant studies (published and unpublished) examining benefits and harms for each intervention		
Background and rationale: choice of comparators	#6b	Explanation for choice of comparators	6	
Objectives	#7	Specific objectives or hypotheses	6	
Trial design	#8	Description of trial design including type of trial (eg, parallel group, crossover, factorial, single group), allocation ratio, and	6	

For peer review only

		framework (eg, superiority, equivalence, non-inferiority, exploratory)		
**Methods: Participants, interventions, and outcomes**				
Study setting	#9	Description of study settings (eg, community clinic, academic hospital) and list of countries where data will be collected. Reference to where list of study sites can be obtained	6	
Eligibility criteria	#10	Inclusion and exclusion criteria for participants. If applicable,	7, 8	

For peer review only

		eligibility criteria for study centres and individuals who will perform the interventions (eg, surgeons, psychotherapists)		
Interventions: description	#11a	Interventions for each group with sufficient detail to allow replication, including how and when they will be administered	8, 9	
Interventions: modifications	#11b	Criteria for discontinuing or modifying allocated interventions for a given trial participant (eg, drug dose change in response to	n/a (non-CTIMPS)	

For peer review only

		harms, participant request, or improving / worsening disease)		
Interventions: adherance	#11c	Strategies to improve adherence to intervention protocols, and any procedures for monitoring adherence (eg, drug tablet return; laboratory tests)	8	
Interventions: concomitant care	#11d	Relevant concomitant care and interventions that are permitted or prohibited during the trial	9	
Outcomes	#12	Primary, secondary, and	10, 11	

For peer review only

		other outcomes, including the specific measurement variable (eg, systolic blood pressure), analysis metric (eg, change from baseline, final value, time to event), method of aggregation (eg, median, proportion), and time point for each outcome. Explanation of the clinical relevance of chosen efficacy and harm outcomes is strongly recommended		

For peer review only

Participant timeline	#13	Time schedule of enrolment, interventions (including any run-ins and washouts), assessments, and visits for participants. A schematic diagram is highly recommended (see Figure)	See table 1, page 25, 26	
Sample size	#14	Estimated number of participants needed to achieve study objectives and how it was determined, including clinical and statistical assumptions supporting any	11	

For peer review only

		sample size calculations		
Recruitment	#15	Strategies for achieving adequate participant enrolment to reach target sample size		48, 49
**Methods: Assignment of interventions (for controlled trials)**				
Allocation: sequence generation	#16a	Method of generating the allocation sequence (eg, computer-generated random numbers), and list of any factors for stratification. To reduce	9, 10	

For peer review only

		predictability of a random sequence, details of any planned restriction (eg, blocking) should be provided in a separate document that is unavailable to those who enrol participants or assign interventions		
Allocation concealment mechanism	#16b	Mechanism of implementing the allocation sequence (eg, central telephone; sequentially numbered, opaque, sealed envelopes), describing any steps to conceal	9, 10	

For peer review only

		the sequence until interventions are assigned		
Allocation: implementation	#16c	Who will generate the allocation sequence, who will enrol participants, and who will assign participants to interventions	9, 10	
Blinding (masking)	#17a	Who will be blinded after assignment to interventions (eg, trial participants, care providers, outcome assessors, data analysts), and how	9, 10	
Blinding (masking):	#17b	If blinded, circumstances under which	n/a	

For peer review only

emergency unblinding		unblinding is permissible, and procedure for revealing a participant's allocated intervention during the trial		
**Methods: Data collection, management, and analysis**				
Data collection plan	#18a	Plans for assessment and collection of outcome, baseline, and other trial data, including any related processes to promote data quality (eg, duplicate measurements,	10, 11	

For peer review only

		training of assessors) and a description of study instruments (eg, questionnaires, laboratory tests) along with their reliability and validity, if known. Reference to where data collection forms can be found, if not in the protocol		
Data collection plan: retention	#18b	Plans to promote participant retention and complete follow-up, including list of any outcome data to be collected for participants who discontinue or	10, 11	

For peer review only

		deviate from intervention protocols		
Data management	#19	Plans for data entry, coding, security, and storage, including any related processes to promote data quality (eg, double data entry; range checks for data values). Reference to where details of data management procedures can be found, if not in the protocol		40-42
Statistics: outcomes	#20a	Statistical methods for	12, 13, 14	

For peer review only

		analysing primary and secondary outcomes. Reference to where other details of the statistical analysis plan can be found, if not in the protocol		
Statistics: additional analyses	#20b	Methods for any additional analyses (eg, subgroup and adjusted analyses)	13, 14	
Statistics: analysis population and missing data	#20c	Definition of analysis population relating to protocol non-adherence (eg, as randomised analysis), and any statistical	13, 14	

For peer review only

		methods to handle missing data (eg, multiple imputation)		
**Methods: Monitoring**				
Data monitoring: formal committee	#21a	Composition of data monitoring committee (DMC); summary of its role and reporting structure; statement of whether it is independent from the sponsor and competing interests; and reference to where further details about its charter can be found, if not in the protocol.		50

For peer review only

		Alternatively, an explanation of why a DMC is not needed		
Data monitoring: interim analysis	#21b	Description of any interim analyses and stopping guidelines, including who will have access to these interim results and make the final decision to terminate the trial		35
Harms	#22	Plans for collecting, assessing, reporting, and managing solicited and spontaneously reported adverse events and other	12	

For peer review only

		unintended effects of trial interventions or trial conduct		
Auditing	#23	Frequency and procedures for auditing trial conduct, if any, and whether the process will be independent from investigators and the sponsor		52
**Ethics and dissemination**				
Research ethics approval	#24	Plans for seeking research ethics committee / institutional review board (REC / IRB) approval	3	
Protocol amendments	#25	Plans for communicating		51

For peer review only

		important protocol modifications (eg, changes to eligibility criteria, outcomes, analyses) to relevant parties (eg, investigators, REC / IRBs, trial participants, trial registries, journals, regulators)		
Consent or assent	#26a	Who will obtain informed consent or assent from potential trial participants or authorised surrogates, and how (see Item 32)	7, 8	

For peer review only

Consent or assent: ancillary studies	#26b	Additional consent provisions for collection and use of participant data and biological specimens in ancillary studies, if applicable	n/a	
Confidentiality	#27	How personal information about potential and enrolled participants will be collected, shared, and maintained in order to protect confidentiality before, during, and after the trial		
Declaration of interests	#28	Financial and other competing interests for	17	

For peer review only

		principal investigators for the overall trial and each study site		
Data access	#29	Statement of who will have access to the final trial dataset, and disclosure of contractual agreements that limit such access for investigators		42
Ancillary and post trial care	#30	Provisions, if any, for ancillary and post-trial care, and for compensation to those who suffer harm from trial participation	n/a	
Dissemination policy: trial results	#31a	Plans for investigators and sponsor to		52, 53

For peer review only

		communicate trial results to participants, healthcare professionals, the public, and other relevant groups (eg, via publication, reporting in results databases, or other data sharing arrangements), including any publication restrictions		
Dissemination policy: authorship	#31b	Authorship eligibility guidelines and any intended use of professional writers		52, 53

For peer review only

Dissemination policy: reproducible research	#31c	Plans, if any, for granting public access to the full protocol, participant-level dataset, and statistical code	18	
**Appendices**				
Informed consent materials	#32	Model consent form and other related documentation given to participants and authorised surrogates	Supplemental material	
Biological specimens	#33	Plans for collection, laboratory evaluation, and storage of biological specimens for genetic or	n/a	

For peer review only

		molecular analysis in the current trial and for future use in ancillary studies, if applicable		

None The SPIRIT Explanation and Elaboration paper is distributed under the terms of the Creative Commons Attribution License CC-BY-NC. This checklist can be completed online using https://www.goodreports.org/, a tool made by the EQUATOR Network in collaboration with Penelope.ai

For peer review only

The TIDieR (Template for Intervention Description and Replication) Checklist*:

TIDieR
Template for Intervention Description and Replication

Number	Item	Detailed description	Where located **	
			Protocol paper (page or appendix number)	Other † (details)
**1.**	**BRIEF NAME**<br>Provide the name or a phrase that describes the intervention.	DREAMS START (Dementia Related Manual for Sleep: Strategies for Relatives)	Page.1 line 1	
**2.**	**WHY**<br>Describe any rationale, theory, or goal of the elements essential to the intervention.	The co-designed intervention supports carers to develop strategies to improve their relative with dementia's sleep. DREAMS START provides information about sleep and dementia, supports carers to use practical *zeitgebers* (cues that influence the person's biological rhythms e.g. regular timing of bed and rising, morning wake-up light, regular meal times) and to establish adaptive stimulus control (e.g. pre-bed settling routine, management of wakeful episodes). It uses strategies to promote de-arousal at night (e.g. relaxation, bedroom comfort, no caffeine or alcohol pre-bed, relaxation) and daytime behavioural activation to maintain alertness and reduce daytime naps. The rationale for this is that it will strengthen the natural	Appendix 2 (Intervention components)<br><br>Page.5 line 20-32<br><br>Page 8 lines 14-30	Rapaport P et al: Dementia. 2018;17(8):976-89.<br><br>Full protocol at https://doi.org/10.1186/ISRCTN13072268

For peer review only - http://bmjopen.bmj.com/site/about/guidelines.xhtml

		circadian rhythmicity and increase sleep pressure in the person living with dementia. The intervention also focuses on helping carers to look after their own (sleep) health and to use behavioural strategies for reducing distress behaviours at night. Each session teaches different relaxation strategies. The rationale for this is that family carers will be able to reduce the impact of the sleep disturbance on their own sleep and having a greater understanding and acceptance of the situation may reduce their own distress.		
**3.**	**WHAT**<br>Materials: Describe any physical or informational materials used in the intervention, including those provided to participants or used in intervention delivery or in training of intervention providers. Provide information on where the materials can be accessed (e.g. online appendix, URL).	The participants receive a paper copy of the DREAMS START manual that they can write in with personalised strategies, sleep diaries and record forms and an individual action plan (once the trial is complete the manuals will be made available online). The participants are also provided with a phototherapy light box and access to recordings of relaxation exercises. If their relative has worn an actigraph prior to the intervention they are given a summary of their relatives sleep pattern with an interpretation alongside their manual.	Page 8 lines 14-30	Full protocol at https://doi.org/10.1186/ISRCTN13072268
**4.**	Procedures: Describe each of the procedures, activities, and/or processes used in the	DREAMS START is delivered over six sessions (see below for mode of delivery). The intervention is delivered by a facilitator who is trained in therapeutic engagement skills and in how to support	Abstract<br><br>Page 8 lines 14-	Full protocol at https://doi.org/10.1186/ISRCTN

For peer review only - http://bmjopen.bmj.com/site/about/guidelines.xhtml

	intervention, including any enabling or support activities.	participants (family carers) to implement and change behaviours in themselves and their relatives. Each session includes practical exercises to develop tailored strategies, between session monitoring and practice tasks, reflection on the previous session and building an individual and written action plan, adding strategies each week. Participants are encouraged to share ideas and strategies with their wider support network and facilitators are encouraged to build on the carer's existing skills and expertise, focusing on what works well and validating challenging or distressing experiences.	30	13072268
**5.**	**WHO PROVIDED**<br>For each category of intervention provider (e.g. psychologist, nursing assistant), describe their expertise, background and any specific training given.	The intervention facilitators are non-clinically qualified psychology graduates. They will receive a two-day training which will focus on dementia and sleep-wake regulation, using actigraphs, empathic listening skills, facilitating behaviour change, using supervision effectively and working collaboratively with family carers and people living with dementia. It will emphasise the need to operate from an inclusive values base and to respect diversity and the existing knowledge and skills of family carers. Facilitators will learn through a combination of seminars, discussion, reflective learning, and guided reading. Facilitators will have fortnightly group supervision with a clinical psychologist with additional individual supervision as requested by facilitators or the study team. The group supervision format makes most effective use of available resources and enables	Page 8 line 32-page 9 line 13	Full protocol at https://doi.org/10.1186/ISRCTN13072268

For peer review only - http://bmjopen.bmj.com/site/about/guidelines.xhtml

		the facilitators to benefit from the professional expertise of their supervisor and the experiences of their peers. Supervision will include case management, skills development, risk management and safe practice and staff support.		
**6.**	**HOW** Describe the modes of delivery (e.g. face-to-face or by some other mechanism, such as internet or telephone) of the intervention and whether it was provided individually or in a group.	The intervention will be delivered flexibly depending on the preference and practical considerations for the participants and facilitators. DREAMS START can be delivered face-to-face, by video conferencing or by telephone, or in a combination of these. Participants will always have a copy of the manual with them for the sessions as well as being guided by the facilitator. The sessions will be delivered to family carers individually unless the person living with dementia wishes (and is able) to participate and other family members or paid care staff can join sessions if that is felt to be useful.	Page 8 lines 29-30	Full protocol at https://doi.org/10.1186/ISRCTN13072268
**7.**	**WHERE** Describe the type(s) of location(s) where the intervention occurred, including any necessary infrastructure or relevant features.	DREAMS START will be delivered in people's homes if face to face (unless participants would prefer to be seen at a clinic or university building for convenience).	Page 8 lines 29-30	Full protocol at https://doi.org/10.1186/ISRCTN13072268
	**WHEN AND HOW MUCH**			

For peer review only - http://bmjopen.bmj.com/site/about/guidelines.xhtml

**8.**	Describe the number of times the intervention was delivered and over what period of time including the number of sessions, their schedule, and their duration, intensity or dose.	The intervention is delivered over six-hour long sessions. This is delivered flexibly with a session every one to two weeks depending on family carer availability.	Abstract<br><br>Page 8 lines 14-30	Full protocol at https://doi.org/10.1186/ISRCTN13072268
**9.**	**TAILORING**<br>If the intervention was planned to be personalised, titrated or adapted, then describe what, why, when, and how.	The intervention is planned to be tailored by the facilitator working with the participant to identify and prioritise specific concerns, develop personalised strategies and note these in the manual and then develop a smart action plan. The individual sleep data from actigraphy can be used by the facilitator to make specific recommendations around time to bed and rise and other actions such as exercise or steps to reduce daytime sleep.	Page 8 lines 14-30	Full protocol at https://doi.org/10.1186/ISRCTN13072268
**10.‡**	**MODIFICATIONS**<br>If the intervention was modified during the course of the study, describe the changes (what, why, when, and how).	Since the intervention was feasible and acceptable during our initial feasibility RCT, with carers responding positively in our qualitative study, we did not make major changes prior to the full RCT. The main change we made was that during the feasibility trial we did deliver the intervention to people living with dementia living alone without a carer present and found that this was impractical and challenging for participants and facilitators. We made minor revisions	Page 8 lines 29-30	Livingston et al (2018) International Psychogeriatrics 2019;31(2):251-265.

For peer review only - http://bmjopen.bmj.com/site/about/guidelines.xhtml

		are based on feedback from family carers for example adding further detail on use of assistive technology and adapted our record forms to make them simpler and easier to use. As the Covid 19 pandemic occurred between the feasibility and full RCT we also adapted the mode of delivery (see question 6 above) away from a fully face-to-face model.		
**11.**	**HOW WELL**<br>Planned: If intervention adherence or fidelity was assessed, describe how and by whom, and if any strategies were used to maintain or improve fidelity, describe them.	To improve fidelity we have a manualised intervention, training and supervision of facilitators (see question 5) and to ensure treatment integrity, facilitators are required to demonstrate competence in delivering the intervention by role-play before we begin recruitment with one of the study clinician co-applicants. We will collect information on trial adherence and attendance. In addition to supervision and training, we will formally monitor fidelity to the intervention. Facilitators will, with participant's permission, record one intervention session per participant, decided by the trial manager randomly generating session numbers. A researcher not involved in the therapy will independently rate fidelity to the manual, using our existing checklist incorporating the key content of each session and four process factors ("keeping the session to time", "keeping the carer focused on the manual", "keeping the carer engaged in the session" and "managing the concerns of the carer").	Page 8 line 32 – page 9 line13	Full protocol at https://doi.org/10.1186/ISRCTN13072268
**12.**‡	Actual: If intervention	N/A as this is protocol.	N/A	

For peer review only - http://bmjopen.bmj.com/site/about/guidelines.xhtml

	adherence or fidelity was assessed, describe the extent to which the intervention was delivered as planned.			

** **Authors** - use N/A if an item is not applicable for the intervention being described. **Reviewers** – use '?' if information about the element is not reported/not sufficiently reported.

† If the information is not provided in the primary paper, give details of where this information is available. This may include locations such as a published protocol or other published papers (provide citation details) or a website (provide the URL).

‡ If completing the TIDieR checklist for a protocol, these items are not relevant to the protocol and cannot be described until the study is complete.

* We strongly recommend using this checklist in conjunction with the TIDieR guide (see *BMJ* 2014;348:g1687) which contains an explanation and elaboration for each item.

* The focus of TIDieR is on reporting details of the intervention elements (and where relevant, comparison elements) of a study. Other elements and methodological features of studies are covered by other reporting statements and checklists and have not been duplicated as part of the TIDieR checklist. When a **randomised trial** is being reported, the TIDieR checklist should be used in conjunction with the CONSORT statement (see www.consort-statement.org) as an extension of **Item 5 of the CONSORT 2010 Statement.** When a **clinical trial protocol** is being reported, the TIDieR checklist should be used in conjunction with the SPIRIT statement as an extension of **Item 11 of the SPIRIT 2013 Statement** (see www.spirit-statement.org). For alternate study designs, TIDieR can be used in conjunction with the appropriate checklist for that study design (see www.equator-network.org).

For peer review only

For peer review only - http://bmjopen.bmj.com/site/about/guidelines.xhtml

BMJ Open

Clinical and cost-effectiveness of DREAMS START (Dementia RElAted Manual for Sleep; STrAtegies for RelaTives) for people living with dementia and their carers. A study protocol for a parallel multi-centre randomised controlled trial.

Journal:	*BMJ Open*
Manuscript ID	bmjopen-2023-075273.R2
Article Type:	Protocol
Date Submitted by the Author:	16-Jan-2024
Complete List of Authors:	Rapaport, Penny; University College London, Division of Psychiatry<br>Amador, Sarah ; University College London, Division of Psychiatry<br>Adeleke, Mariam; University College London, Department of Statistical Science<br>Banerjee, Sube; Brighton and Sussex Medical School, Centre for Dementia Studies<br>Barber, Julie; University College London, Department of Statistical Science<br>Charlesworth, Georgina; University College London, Division of Psychology and Language Sciences; North East London NHS Foundation Trust<br>Clarke, Christopher; Tees Esk and Wear Valleys NHS Foundation Trust<br>Connell, Caroline; Tees Esk and Wear Valleys NHS Foundation Trust<br>Espie, Colin; University of Oxford, Sleep and Circadian Neuroscience Institute<br>Gonzalez, Lina; University College London, Research Department of Primary Care and Population Health<br>Horsley, Rossana; Alzheimer's Society<br>Hunter, Rachael; University College London, Research Dept of Primary Care and Population Health<br>Kyle, Simon; University of Oxford, Sleep and Circadian Neuroscience Institute<br>Manela, Monica; University College London, Division of Psychiatry<br>Morris, Sarah; Tees Esk and Wear Valleys NHS Foundation Trust<br>Pikett, Liam; University College London<br>Raczek, Malgorzata; BSMS Centre for Dementia Studies<br>Thornton, Emma; Tees Esk and Wear Valleys NHS Foundation Trust<br>Walker, Zuzana; University College London, Division of Psychiatry<br>Webster, Lucy; University College London, Division of Psychiatry; University of Kent<br>Livingston, Gill; University College London, Division of Psychiatry
**Primary Subject Heading**:	Neurology
Secondary Subject Heading:	Mental health

Keywords:	Dementia, Health Services for the Aged, Clinical Trial

SCHOLARONE™
Manuscripts

Clinical and cost-effectiveness of DREAMS START (Dementia RElAted Manual for Sleep; STrAtegies for RelaTives) for people living with dementia and their carers. A study protocol for a parallel multi-centre randomised controlled trial.

Corresponding author:
Dr Penny Rapaport
Principal Research Fellow / Clinical Psychologist*
Tel: 0207 679 9647
Email: p.rapaport@ucl.ac.uk

Authors:
Penny Rapaport[1]
Sarah Amador[1]
Mariam Adeleke[2]
Sube Banerjee[3]
Julie Barber[2]
Georgina Charlesworth[4]
Christopher Clarke[5]
Caroline Connell[6]
Colin A. Espie[7]
Lina Gonzalez[9]
Rossana Horsley[8]
Rachael Hunter[9]
Simon D Kyle[7]
Monica Manela[1]
Sarah Morris[5]
Liam Pikett[1]
Malgorzata Raczek[6]
Emma Thornton[5]
Zuzana Walker[1]
Lucy Webster[1, 10]
Gill Livingston[1]

For peer review only

1 Department of Mental Health of Older People, Division of Psychiatry, University College London, London, UK

2 Department of Statistical Science, University College London, London, UK

3 Faculty of Health, University of Plymouth, England, UK

4 Division of Psychology and Language Sciences, University College London, London, UK and North East London NHS Foundation Trust, London, UK

5 Tees, Esk and Wear Valley NHS Foundation Trust , UK

6 BSMS Centre for Dementia Studies, Brighton, UK

7 Sir Jules Thorn Sleep & Circadian Neurosciences Institute, University of Oxford, UK

8 Patient and Public Involvement member

9 Department of Primary Care and Population Health, Institute of Epidemiology and Health Care, University College London, London, UK

10 University of Kent, UK

Word count: 5530

Figures: 2

Supplemental materials:

Supplementary Table 1: Schedule of visits and assessments

Appendix 1: Informed consent form

Appendix 2: Summary of intervention components

ABSTRACT (299/300)

Introduction: Many people living with dementia experience sleep disturbance and there are no known effective treatments. Non-pharmacological treatment options should be first-line sleep management. For family carers relatives' sleep disturbance leads to interruption of their sleep, low mood, and breakdown of care. Our team developed and delivered DREAMS START (Dementia RElAted Manual for Sleep; STrAtegies for RelaTives), a multimodal non-pharmacological intervention, showing it to be feasible and acceptable. The aim of this randomised controlled trial (RCT) is to establish whether DREAMS START is clinically and cost-effective in reducing sleep disturbances in people living with dementia living at home compared to usual care.

For peer review only

Methods and analysis: We will recruit 370 participant dyads (people living with dementia and family carers) from memory services, community mental health teams and the Join Dementia Research Website in England. Those meeting inclusion criteria will be randomised (1:1) either to DREAMS START or to usual treatment. DREAMS START is a six-session (1hour/session), manualised intervention delivered every 1-2 weeks by supervised, non-clinically trained graduates. Outcomes will be collected at baseline, 4 months and 8 months with the primary outcome the Sleep Disorders Inventory (SDI) score at 8 months. Secondary outcomes for the person with dementia (all proxy) include quality of life, daytime sleepiness, neuropsychiatric symptoms and cost-effectiveness. Secondary outcomes for the family carer include quality of life, sleep disturbance, mood, burden and service use and caring/work activity. Analyses will be intention-to-treat and we will conduct a process evaluation.

Ethics and dissemination: London – Camden & Kings Cross Ethics Committee (20/LO/0894) approved the study. We will disseminate our findings in high-impact peer-reviewed journals and at national and international conferences. This research has the potential to improve sleep and quality of life for people living with dementia and their carers, in a feasible and scalable intervention.

Trial registration number: ISRCTN13072268

ARTICLE SUMMARY

Strengths and limitations of this study:

- This multi-centre randomised controlled trial recruiting 370 participants will be the largest, and only fully powered randomised controlled trial of a multicomponent non-pharmacological intervention targeting sleep disturbance in people living at home with dementia to date.
- The study will provide conclusive clinical and cost-effectiveness evidence for the DREAMS START intervention.
- Robust cost-effectiveness data and a mixed-methods process evaluation will, if the intervention is effective, support the potential roll out of the intervention into UK NHS services and other international care settings.
- In our feasibility trial, we found that actigraphy had lower acceptability, and validity than needed as an outcome measure, we are therefore using proxy questionnaires to measure outcomes and using actigraphy as a measure of movement to inform our process evaluation.
- The nature of the intervention is such that participants and those delivering the intervention will not be blinded to treatment allocation.

For peer review only

For peer review only

INTRODUCTION

The 47 million people living with dementia worldwide is projected to increase to 131 million by 2050; at an estimated trillion United States Dollar (USD) cost(1). Sleep disturbance affects 25-40% of people living with dementia(2-6) with a meta-analysis finding a pooled prevalence of 26% among people living at home with dementia, (24% of people living with Alzheimer's disease (AD) and 49% of people living with Lewy body dementia(6). Sleep disturbance affects all aspects of mental and physical functioning and quality of life(7) and may increase the risk of developing or worsening of AD(8). People living with dementia may wake during the night unaware of the time or be distressed or disorientated. Family members provide most care for the two-thirds of people living with dementia living at home(9), finding it difficult to cope with persistently disturbed sleep, impacting on quality of life(10, 11). Sleep disturbances also predict family carer depressive symptoms, burden and care home admission, elevating the individual, societal and economic impact of dementia(12-14). Although there is not currently data available for reliable estimates for the costs associated with sleep disturbances and dementia(15), we do know that sleep disturbances can be a contributing factor to transition to care homes which increases health and social care resource costs. It may also increase the likelihood of falls and loss of weight as people are more tired during the day and this may increase use of resources(16).

As with other older people, most people living with dementia have other illnesses and over 90% have at least one long-term health condition and may experience pain, discomfort or mood disturbances(17) which can all contribute to sleep disturbance(18). Moreover, neurodegeneration of brain structures involved in the regulation of sleep and circadian rhythms, including the suprachiasmatic nucleus (SCN), likely mediate changes in sleep timing, sleep continuity, and sleep architecture in dementia(19). Dementia can therefore lead to impaired sleep initiation, reduced night-time sleep, difficulty maintaining sleep, increased night-time wandering, and excessive daytime sleepiness(2, 3, 20). Treating sleep problems may not only improve wellbeing, daytime functioning, and quality of life of those living with dementia but given the bi-directional relationship between sleep disruption and amyloid deposition and tau pathology(21) could potentially slow disease progression(22).

There is no conclusive evidence that any therapy to treat sleep disturbance in dementia is effective(11, 15, 23). A Cochrane review of pharmacotherapies found no conclusive randomised controlled trial (RCT) evidence for people living with dementia(24). Antipsychotics and benzodiazepines have adverse effects(25) and increase mortality in older adults(26). Pharmacological interventions, including melatonin, are not recommended as treatments(27) and

For peer review only

patients and their doctors prefer non-drug approaches for sleep problems(28). Cochrane reviews have found insufficient evidence for light therapy alone in dementia(15, 29). A recent Cochrane review of non-pharmacological interventions for sleep disturbance in people living with dementia found no conclusive evidence from the 19 included RCTs with none of the included studies identified to be at low risk of bias(15). This review found some positive effects for carer focused interventions and for interventions which promoted physical and social activities. Additionally, only four of the included studies recruited people living at home in the community, with most conducted in nursing homes which highlights a gap in good quality evidence conducted with people living with dementia in their own homes. As sleep disturbances in people living with dementia have mixed causes, promising interventions are likely to be multi-component. Wilfling et al conclude that multimodal and complex interventions have the strongest potential to improve sleep disturbance in people living with dementia(15). Although there is no conclusive evidence of any effective non-pharmacological treatment for sleep disturbances in people living with dementia(30), two pilot studies in community-dwelling people living with dementia, and our DREAMS START (Dementia RElAted Manual for Sleep; STrAtegies for RelaTives) feasibility RCT, found potential benefits of combining light with other components, including sleep education and hygiene, exercise, daytime activities and cognitive behavioural therapy (CBT) (31-33). We need clinically and cost-effective ways to improve disrupted sleep for people living with dementia, their families, health and social care systems, society and global economies.

This research builds on our successful feasibility RCT (NIHR/HTA 14/220/06)(31, 34). Our vision was to synthesise and add to incomplete evidence, co-produce a multi-component non-pharmacological intervention, and deliver it individually, tailoring it to fit each participant's needs. We co-produced DREAMS START using the best available evidence, patient and public involvement (PPI) and our clinical and research expertise in sleep and dementia(35). We incorporated the different components, including light, increased activity and exercise and carer's support which show promise in improving outcomes for people living with dementia and sleep disturbance(15) The intervention is delivered to family carers, who implement strategies to reduce the person with dementia's sleep disturbances. It uses natural daylight (where feasible) and if necessary, timed phototherapy to strengthen and stabilise sleep-wake timing. Additionally, it alerts carers to consider pain, uses strategies to increase comfort, reduce anxiety, increase daytime activity and uses CBT for sleep management, which Cochrane reviews found effective for older adults and family carers of people without dementia(36, 37). Our feasibility RCT found that the study design and the intervention were feasible and acceptable; 63/95 (65%; 95% CI 55% to 75%) eligible referrals consented, 62/95 (65%;

For peer review only

95% CI 55% to 75%) were randomised, and 37/42 (88%; 95% CI 75% to 96%) randomised to the intervention adhered to it. Qualitative interviews indicated that it was acceptable.

Study objectives

This study aims to determine whether our manualised, multi-component, non-pharmacological treatment package delivered by supervised non-clinically qualified psychology graduates, will deliver significant benefits for people living with dementia and their family carers. It is important to demonstrate our intervention is clinically and cost effective immediately post-intervention and to establish whether this effect is sustained. Since this is an intervention focused upon sustaining behaviour change and continuing to use successful strategies, we would anticipate that the effect will be sustained over time and may increase as behaviour change is embedded. This would have clear impact for individual and within health services. Therefore, our primary outcome is a measure of sustained effectiveness at eight-months We have also included analysis at four-months to establish if the intervention is effective immediately post intervention. Additional aims are to assess the process and fidelity of delivery of the intervention and explore the experiences of family carers who are the recipients of the intervention sessions to change the sleep of the person with dementia. We will use the data generated to make evidence-based clinical practice recommendations on managing sleep disturbance in dementia.

Primary objective: To determine whether the DREAMS START intervention improves sleep disturbance in people living with dementia at home at 8 months compared to usual NHS treatment.

***Secondary objectives*:** To determine:

1. Whether the DREAMS START intervention improves sleep disturbance in people living with dementia at home at 4-months.
2. Whether it reduces daytime sleepiness (at 4 and 8 months).
3. Whether it improves people living with dementia's quality of life (at 4 and 8 months).
4. The probability that it is cost-effective compared to usual NHS treatment in regard to costs and preference based health related quality of life
5. The role of psychotropic medication and melatonin in any change.
6. Whether it improves family carer quality of life (at 4 and 8 months).
7. Whether it improves family carer's sleep and decreases their affective symptoms and burden (at 4 and 8 months).
8. The mechanisms of change.
9. If effective, how we can optimise the intervention for implementation at scale in the NHS.

For peer review only

METHODS AND ANALYSIS

Trial design

This is a multi-centre, parallel-group, superiority randomised controlled trial (RCT) with masked outcome assessment to test whether the DREAMS START intervention improves sleep disturbance in people living with dementia compared to usual treatment. Participants will be enrolled in the trial for eight months, assessments will take place at baseline, four months, and eight months post-randomisation (see Figure 1 for trial flow diagram). The trial is registered with the ISRCTN and an independent trial steering committee (TSC) and data monitoring and ethics committee (DMEC) provide oversight.

Participants and recruitment

Potential participant dyads (family carers and people living with dementia) will be recruited from memory services and older adult mental health services in NHS Trusts in London, Essex, Sussex, and Tees, Esk and Wear Valleys and the Join Dementia Research (JDR) website. JDR is a free, secure online and telephone service developed and launched in 2014/2015 by the National Institute of Health and Care Research (NIHR).

Eligibility will be checked by researchers during initial screening calls to potential participants. People will be included in the study if they meet the following criteria:

1. People living with dementia (any type/severity/on any or no medication), except those currently drinking alcohol heavily.
2. Sleep disorders inventory (SDI) score ≥4. The SDI is a valid and reliable standalone tool for sleep disorder in people with dementia(38). Those who score ≥4 have clinically significant sleep disturbance.
3. Sleep that patient or their family judge as problematic. This is a pragmatic study and if the patient and family are unconcerned, treatment is unnecessary, as is the case in clinical practice. We asked the referrers at the point of entry into the study and assessors asked the family carers and person living with dementia again as part of eligibility criteria by during screening calls and initial assessment meetings.
4. Patient with capacity gives consent, or if not capacitous, consultee gives consent with patient not unwilling.
5. Family carer gives informed consent.
6. Family carer supports the person with dementia emotionally or practically at least weekly.

For peer review only

7. Person with dementia lives in their own home at the beginning of the study with someone present at night.

Exclusion criteria (any):

1. Known primary sleep breathing disorder diagnosis preceding dementia (e.g. sleep apnoea).
2. Current known heavy alcohol use (Alcohol Use Disorders Identification Test – Consumption (AUDIT C) Score ≥8). We have this criterion as during our earlier feasibility trial two participants drank alcohol during the intervention sessions during the day. These people were unable to work with a plan to change their sleep, which involved reducing alcohol, and we were concerned for the safety of our facilitators visiting their homes alone. The participants dropped out.
3. People unavailable for >3 weeks (e.g. planned holiday or hospital admission) of intervention and follow-up.
4. Currently enrolled in another non-pharmacological dementia RCT.

If potential participants meet eligibility criteria, researchers will offer either a baseline home visit or if necessary, an appointment via telephone or video call. At this appointment, if potential participants are willing, researchers will obtain written informed consent (see Appendix 1) or where necessary (audio-recorded) verbal consent to take part in the study from family carers and people living with dementia who have capacity (or a personal consultee will sign for people living with dementia who lack capacity to consent). They will then complete the baseline assessments (see Assessments below, and Supplementary Table 1).

Intervention

DREAMS START is a six-session manual-based intervention for carers of people living with dementia to make changes to improve their relatives' sleep(31, 34). People living with dementia can join their carers in sessions if they wish. (see Appendix 2 for a summary of intervention components). The intervention provides information about sleep and dementia, supports carers to use practical *zeitgebers* (cues that influence the person's biological rhythms e.g. regular timing of bed and rising, morning wake-up light, regular mealtimes) and to establish adaptive stimulus control (e.g. pre-bed settling routine, management of wakeful episodes). It uses strategies to promote de-arousal at night (e.g. relaxation, bedroom comfort, no caffeine or alcohol pre-bed, relaxation) and daytime behavioural activation to maintain alertness and reduce daytime naps. The intervention also focuses on helping carers to look after their own (sleep) health, incorporating aspects of CBT. We use actigraphic data from devices worn by participants with dementia at baseline to inform the

personalisation of intervention strategies. Over the sessions, carers will build a personalised action plan to continue using after the sessions are completed. We will aim to deliver the sessions weekly or fortnightly depending on the availability of the carer but will be flexible to maximise adherence. We will deliver either in person, online via video call or by telephone depending on the preferences of the carer.

Training and supervising facilitators: DREAMS START Facilitators will be non-clinically qualified psychology graduates who have participated in a two-day training programme delivered by the study team online. We have successfully adopted this approach in previous trials(39-41). This level of expertise ensures the breadth of necessary skills (to impart information, knowledge of dementia, mental health and knowing when to ask for help). Our team has extensive experience in training psychology graduates and psychology assistants in memory and mental health services to deliver non-pharmacological interventions. DREAMS START was designed to be delivered by this same group, to maximise the potential for delivery at scale and at low cost (compared to less available and more expensive qualified psychologists or nurses delivering the intervention) and is compatible with a range of commissioning models. Training will focus on dementia and sleep-wake regulation, empathic listening skills, facilitating behaviour change, interpreting actigraphy, using supervision effectively and working collaboratively with family carers and people living with dementia. Facilitators will receive fortnightly group supervision with a clinical psychologist with additional individual supervision as requested by facilitators or the study team. We used this model in our feasibility RCT and found that the intervention was delivered to a high level of fidelity and was acceptable to and the facilitators positively experienced by participants.

Usual treatment: We are comparing the new treatment with treatment as usual (TAU). Participants randomised to the new treatment will also receive usual care. TAU varies according to where the person with dementia is treated and their individual needs, but incorporates National Institute for Health and Care Excellence (NICE) guidelines for dementia and consists of assessment, diagnosis, symptomatic interventions, risk assessment and management, advice and information(27). There is currently no consistent approach to the treatment of sleep difficulties in dementia. We expect usual treatment to reflect that identified during our feasibility trial, where interventions were medical, psychological and social(31). During our feasibility trial, 45% of participants were prescribed one or more psychotropic medications, with 11% prescribed anxiolytic or hypnotic medication. We will not exclude those taking medication for sleep but will note psychotropic medication prescribed and taken.

For peer review only

Randomisation, allocation concealment and blinding

The Trial Manager will perform the randomisation procedure, after consent and baseline data collection are completed. If the Trial Manager is not available, randomisation will be by a member of the research team who is not involved in participant recruitment or follow-up. Randomisation will be provided by a web based system using the company Sealed Envelope. Randomisation will occur at the level of the patient and will be blocked and stratified by site using a 1:1 intervention: treatment as usual ratio. Participants will be assigned to treatment groups through consecutive allocation of participant numbers and the use of a Trial Participant Log. The Trial Manager will notify the intervention facilitators of allocation, who will either arrange the intervention sessions or inform the participant that they have been randomised to the control arm of the trial. Researchers collecting data will be masked to group allocations. The person facilitating the intervention will be different from the researcher conducting the follow-up assessments, if an individual has both roles this will not be in the same research sites. Clinical supervision will be conducted in separate groups based on different research sites to avoid unmasking of intervention facilitators who may also be collecting research data in a different site to that they are delivering in.

Participants and those delivering the intervention will be unmasked and aware of intervention status, which is common in behavioural and psychological intervention trials(42). As follow up assessments will be masked, there is a small risk that assessors may become unmasked accidentally by the participant or carer. We will minimise this risk by the following: assessors will remind participants at each stage that they must not discuss their intervention with their assessor and remind participants to hide any study related materials or equipment. If an assessor does become unmasked, we will make a note of this and ask an alternative assessor to complete future outcome measures for this participant. We have successfully adopted these procedures in previous trials of non-pharmacological interventions(31, 40, 43).

Assessments

The primary outcome is sleep disturbance at 8 months and will be measured using the SDI. The SDI is validated for measuring sleep disturbance in people living with dementia and describes the frequency and severity of sleep-disturbed behaviours. It is validated against clinical variables and used in recent promising pilot studies of non-pharmacological interventions(31-33, 44). The SDI will be collected at baseline, 4 months, and 8 months post-randomisation. We originally envisaged the primary outcome in this study would be sleep inferred through actigraphy, which fits with chosen primary outcomes in the recent Cochrane review of non-pharmacological interventions for sleep

For peer review only

disturbance in dementia (15), However, the feasibility study indicated its unsuitability. Actigraphy is not validated in people with dementia and infers sleep from lack of movement. Many carers in our feasibility study informed us it was inaccurate e.g. one participant lay still in bed and screamed at night and actigraph output indicated excellent sleep. Others had movement disorders which are common in some dementias and at times did not lie still although they and their family thought they were asleep. In addition, markers of going to bed and getting up, which are necessary for actigraphy data interpretation (for at least seven days; the "gold" standard), were only available for 68% of people randomised in our earlier study(45). Conversely, in our feasibility RCT, the SDI was completed at follow-up by 92% of those recruited. This appeared the best way to measure sleep disturbance, and family members in the trial and PPI said that they found it relevant and reflected their difficulties. All secondary outcome measures will be taken at baseline, 4 months, and 8 months (see Supplementary Table 1).

Secondary outcomes for the person living with dementia are all proxy measures to minimise burden. These include neuropsychiatric symptoms (Neuropsychiatric Inventory (NPI))(46), daytime sleepiness (Epworth sleepiness scale (ESS))(47), and quality of life (DEMQOL-Proxy)(48). The DEMQOL-Proxy will also be used in the cost-effectiveness analysis to calculate Quality of life Adjusted Life Years (QALY). A Modified Client Service Receipt Inventory (CSRI)(49) and EQ-5D 5 level (EQ-5D-5L)(50) proxy will be collected to inform the cost-effectiveness analysis and we will also collect details of medication including side effects. We will collect one week of actigraphy for the person living with dementia (using the Axivity AX3)(51)) from baseline and at 4 and 8-month follow-ups) to inform understandings of the mechanism of change of the intervention. The AX3 is an actigraph, a small, non-invasive device that is worn on the wrist like a watch that measures movement. Typically sleep is inferred from this data from lack of movement. We used actigraphs during our feasibility DREAMS START study and found that they were acceptable to many people living with dementia. We will use the data collected in our process evaluation, exploring changes in activity and how this relates to other outcomes. For family carers, we will assess sleep disturbance (Sleep Condition Indicator (SCI))(52), carer anxiety and depression (Hospital Anxiety and Depression Scale (HADS))(53), carer burden (Zarit Burden Interview (ZBI))(54) and health related quality of life (Health Status Questionnaire (HSQ-12))(55). To inform the cost-effectiveness analysis a Modified CSRI(49) incorporating the Valuation of Informal Care Questionnaire (iVICQ) a measure of carer time and activity and the Brief Work Productivity and Activity Impairment (WPAI) a measure of productivity loss as well as the EQ-5D-5L(50) will be collected at each time point.

For peer review only

Sample size calculation

We used the standard deviation (SD) of baseline SDI scores (15.74) and the correlation between baseline and follow-up measurements (0.57) observed in our feasibility trial for the power calculation(31). There is no published SDI minimum clinically important difference. We aim to detect a difference of ≥5.5 points, consistent with important differences identified through consultation with experts. This corresponds to a small to medium effect size of 0.35 and is realistic (an average difference of 5.6 was observed in feasibility work). To account for potential facilitator clustering in the intervention arm, we assumed an intracluster correlation coefficient (ICC) as observed in earlier studies(40, 43) (0.03) and approximately 15 participants per facilitator. To achieve equal allocation, the initial calculation, before adjustment for clustering, was based on unequal allocation (1:0.75) which, after inflation for clustering in the intervention arm resulted in 1:1 allocation.

Based on these assumptions, to detect a difference in average SDI of 5.5 between intervention and TAU with power 90% and 5% significance requires 370 participants; 185 in each arm. This calculation allows for up to 15% drop out and includes an inflation for potential non-normality of SDI (as observed in feasibility work)(56). Calculation of sample size was carried out using STATA version 14.

Adverse events

All adverse events (except those listed below as expected) will be recorded in the medical records/case notes/source data in the first instance and will be recorded with clinical symptoms and accompanied by a simple, brief description of the event, including dates as appropriate. All adverse events will be recorded until the participant has completed the intervention.

All serious adverse events (except those listed below as expected) will be recorded in the case report form (CRF) and SAE log. This is because the intervention is not invasive, already has a well-known safety profile and therefore, the collection of AE data will not add any value to the safety profile of the intervention. We will collect information on any planned hospitalization at baseline and these will not be considered adverse events. Based on our feasibility work we expect few side effects specific to the intervention. The events listed below describe expected procedural/disease-related AEs/SAEs:

- Emotional distress or discomfort as a result of the questionnaires or intervention.
- Falls as a result of increased physical activity or getting out and about more or because of other age-related conditions.
- Death, hospitalisation due to common causes related to dementia (e.g. UTIs, delirium, common infections) or transition to a care home as a result of the progression of illness.

For peer review only

Analysis plan

Statistical analysis

For each randomised group we will summarise the primary outcome (SDI at 8-month) using means with standard deviations and medians with interquartile ranges. We will also graphically examine the distribution of the score and average scores over time. We will describe the effect of the intervention using the difference in mean SDI score calculated with a 95% confidence interval. This estimate will be obtained from a 3 level linear mixed effects regression model which has random effects to allow for repeated outcome measurements at 4 and 8 months and for clustering by facilitator in the intervention arm (57). The model will include as fixed effects a treatment group indicator, baseline SDI score, study site, a time indicator and an interaction between treatment group and time. If assumptions of the regression model are violated, the SDI score will be analysed after appropriate transformation (e.g. a log transformation). We will calculate the intra-cluster correlation coefficient (with 95% confidence interval) to describe facilitator clustering.

We will carry out all analyses by intention to treat (ITT), comparing the groups as randomised. Analyses of secondary clinical outcomes will take a similar approach to the analysis of the primary outcome. Analyses will be based on people with available outcome data at 4 or 8 months and will rely on an assumption that data is missing at random. We will describe the number (%) of participants with missing outcome in each group, look at reasons for missingness and consider characteristics of the patients excluded from the ITT analysis. In a sensitivity analysis, we will re-estimate the treatment effect with additional adjustment for baseline predictors of missingness. Further analyses based on multiple imputation methods will include use of pattern mixture models under pre-defined missing not at random scenarios(58).

A separate statistical analysis plan (available to download from https://doi.org/10.1186/ISRCTN13072268) will predefine the detail of all intended analyses.

Health economic analysis

We will calculate the incremental cost per QALY gained of DREAMS START plus TAU compared to TAU only, over 8 months from a health and social care cost perspective using the EQ-5D-5L proxy to calculate QALYs in line with NICE guidance. Secondary analyses will calculate QALYs using the DEMQOL and relevant tariff and include impact and cost of carers (paid as well as family and close others) out of pocket costs and relevant wider societal costs.

For peer review only

We will include the EQ-5D-5L (a generic measure of health-related quality of life) for carers in the economic evaluation. We will not measure carer productivity loss alone, some carers will be retired, so measuring productivity loss, which focuses on absenteeism from paid employment and presenteeism within paid employment, is not sufficient here. Instead, we will capture the equivalent of "productivity loss" in our carer population by measuring days off and changes in employment such as moving from full-time to part-time employment and retirement. This will be costed using the human capital approach. The most important component of this analysis will be the impact on carer time and healthcare resource use which will be measured using the Valuation of Informal Care Questionnaire and a modified CSRI respectively. We will also include the brief Work Productivity and Activity Impairment (WPAI) questionnaire for caregivers, to ensure we fully capture productivity losses in carers that are in employment.

We will use the number of sessions attended, duration of the session and grade of the staff delivering the session to calculate participant-level costs of DREAMS START, in addition to the training and supervision cost. We modified the CSRI based on data from our feasibility study and will use costs from published sources to cost healthcare resource use.

Carers will complete the questionnaires about resource use, time spent caring and productivity loss in the past 4 months at baseline, 4-, and 8 -month. We will calculate QALYs as the area under the curve, adjusting for baseline, over 8 months using the EQ-5D-5L and DEMQOL-Proxy to calculate QALYs. We will report descriptive statistics for resource use, costs and QALYs for baseline, 4 and 8 -month. We will calculate the mean incremental costs and QALYs of DREAMS START compared to usual care over 8 months using patient-level data linear regression, adjusting for site and facilitator clustering in line with the statistical analysis plan, with 95% confidence intervals, cost-effectiveness planes and cost-effectiveness acceptability curves calculated using bootstrapping. We will utilise the "net monetary benefit approach" to construct cost-effectiveness acceptability curves to calculate the probability that the intervention is cost-effective for a range of decision thresholds, ranging from £0 to £100,000. We will conduct sensitivity analyses for any assumptions made. We will assess the level and type of missing data and in conjunction with the statistician determine the most appropriate method for accounting for missing data, which is likely to be multiple imputation using chained equations.

The full Health Economics Analysis Plan (HEAP) is available to download from https://doi.org/10.1186/ISRCTN13072268.

For peer review

Process evaluation

In line with the updated MRC framework for developing and evaluating complex interventions(59) we will conduct a process evaluation to explore how the intervention works and in what contexts, this will be informed by our draft logic model (see Figure 2) which prespecifies how we expect the intervention to work and what factors may contribute to this.

Qualitative analysis

We will purposively sample and conduct qualitative interviews with 15-20 participants (selected for maximum variation, men and women, completers and non-completers, spouses and non-spouse family carers and people living with dementia, across sites) and 5 staff delivering the intervention. We will ask about their experiences of different elements of the intervention including the content, form and process of delivery, barriers and facilitators to implementing strategies and perceived impact of the intervention for carers and people living with dementia. We will transcribe qualitative data verbatim, anonymise the transcripts and use NVivo 12. We will use a thematic analytic approach(60) informed by our draft logic model and the Theoretical Domains Framework (61). The team will organise data into preliminary themes iteratively identifying similarities and differences in the data. We will triangulate qualitative data with fidelity ratings, intervention adherence (session attendance; action plans, strategy tried) and quantitative outcomes (including patterns in activity from actigraphy).

Quantitative analysis

In considering change mechanisms, in line with our draft logic model, the analysis will focus on movement as a potentially important indicator. The intervention incorporates increasing daytime activity and reducing napping to increase pressure to fall asleep at night for the person with dementia as well as strengthening the relationship between bed and sleep at night-time and using light to reinforce circadian rhythmicity. As such we would expect increased movement during the day and decreased movement at night which we will explore via actigraphy. We will consider rest-activity amplitude, reflecting the relative difference between the least active five hours (L5) and the most active ten hours (M10) in the day. These will be recorded via the actigraphs worn for a week at baseline and for a week at the 4 and 8--month follow-up points. The mediating effect of these measures on our primary and secondary outcomes will be examined using the stepped approach described by Whittle et al (62). Models fitted in all steps will be mixed models that account for facilitator clustering.

For peer review only

Fidelity analysis

To analyse the fidelity of delivery of DREAMS START, we will assess the number of appointments delivered across all intervention participants. Checklists will be applied independently of the facilitator to a random selection of one recorded intervention session for each participant. A mean fidelity score will be produced by dividing the number of items on the checklist identified as being delivered, by the number of items on the checklist that should have been delivered per appointment, per researcher and across all appointments. We will adopt thresholds used in other intervention fidelity work: where 81–100% constitutes high fidelity, 51-80% is moderate fidelity and 50% or lower constitutes low fidelity(63).

Patient and public involvement

We are partnered by Alzheimer's Society and their research network volunteers, including our co-applicant RH, who has experience as a carer and will continue her input, having contributed to our earlier feasibility study and development of the grant application for this RCT. We have a virtual PPI group and PPI members on our TSC and Trial Management Group (TMG), to provide partnership and enhance the relevance, practicality and utility of our materials and findings. They have advised on the language and content of information sheets, contributed to the revision of the intervention having been integral to the initial intervention co-production and are working with us on knowledge exchange and dissemination activities.

STRENGTHS AND LIMITATIONS

Few trials have tested interventions for sleep in dementia wholly with those living at home and this will be the largest RCT to date testing a multi-component, non-pharmacological intervention with those living with dementia and sleep disturbance. There are a number of potential limitations to our trial protocol. We have excluded those living alone without someone present at night which reduces the generalizability of our findings to the excluded group, who often have high levels of need and potential risk. This decision was based upon our earlier feasibility trial when we included those living alone. When no one else was there at night, carers (family or paid) were unable to implement strategies, like, a scheduled bedtime or wind down routine. It was also difficult to gain reliable information about the sleep patterns of people with dementia living alone and when the intervention was delivered to people living with dementia alone this was often confusing for them. We have also noted above that we could not conduct a double-blind trial of the DREAMS START intervention which potentially increases the risk of performance bias.

For peer review only

ETHICS AND DISSEMINATION

London – Camden & Kings Cross Research Ethics Committee (Reference: 20/LO/0894) approved the study on 18th September 2020.

We will disseminate our findings in high-impact peer-reviewed journals and at national and international conferences. We will present findings in appropriate local forums for health and social care professionals; participants who have indicated they are interested in the results will be sent a lay summary. We are working with an extensive network of academic researchers, policymakers, stakeholders and PPI, acting as a 'Community of Interest' to advise on and ensure acceptability for the dissemination of the research, and to ensure both national and international reach. We aim to produce an implementation toolkit alongside our DREAMS START intervention and facilitator training materials.

This proposed research has the potential to improve sleep and quality of life for people living with dementia and their family carers, in a feasible and scalable intervention, without medication side effects, and to elucidate the mechanisms of impact. If clinically effective, our intervention should be cost-effective: it is cheap and potentially will reduce the health and social care burden, particularly if it delays care home admission.

TRIAL STATUS

The trial commenced recruitment in February 2021 and completed recruitment in March 2023 with final primary outcome data collected by November 2023 and the final report will be submitted in May 2024.

Author's contributions

All authors made a substantial contribution to this work. PR drafted the manuscript. All authors were involved in revising the manuscript, giving final approval of the version to be published and agree to be accountable for all aspects of the work. GL and PR conceived of the study and acquired funding. GL, PR, JB, RHu, CCl, SB, MR, GC, ZW, LW, CE, SDK, RHo, SA, MA, LG, MM, SM, ET, CCo and LP contributed to developing the protocol and its implementation.

Funding Statement

This study/project is funded by the National Institute for Health and Care Research (NIHR) Health Technology Assessment Programme (NIHR HTA 128761). The views expressed are those of the author(s) and not necessarily those of the NIHR or the Department of Health and Social Care.

For peer review only

SDK and CAE are supported by the NIHR Oxford Health Biomedical Research Centre.

Competing interests

The present manuscript is supported by a National Institute for Health and Care Research (NIHR) Health Technology Assessment Programme (HTA) grant (NIHR HTA 128761). SB declares grants from NIHR, CIHR, ESRC, HEE, ESPRC, Alzheimer's Society, and the Alzheimer's Association with no COI with current work. CE declares grants from NIHR-HTA, NIHR-EME, NIHR-BRC, Wellcome Trust with no COI with current work. MR declares a grant from NIHR ARC KSS with no COI with current work. Outside the submitted work SK declares non-financial support from Big Health Ltd. in the form of no cost access to the digital sleep improvement programme, Sleepio, for use in clinical research. Outside the submitted work CE declares stock/stock options and other salary contributions from Big Health Limited developers of Sleepio. Outside the submitted work SSB declares personal fees and non-financial support from Lilly, personal fees from Boehringer-Ingelheim, personal fees from Axovant, personal fees from Lundbeck, personal fees from Nutricia and honoraria from the Hamad Medical Service for lectures and talks. Outside the submitted work MR declares Honorarium for presentation on Lewy body dementias for GE. SB is a trustee of the Alzheimer's Society and NED at the Somerset NHS Foundation Trust. CE is deputy editor of the Journal of Sleep Research and on the editorial board of Sleep Medicine reviews.

Data Sharing statement

The qualitative and quantitative datasets generated during and/or analysed during the current study will be available upon request from PRIMENT CTU Data Management Group on priment@ucl.ac.uk in collaboration with members of DREAMS START Trial Team. Any request for data must come through to PRIMENT CTU in the first instance and where the request is reasonable, anonymised datasets, stored on the publicly available UCL Research Data Repository https://rdr.ucl.ac.uk/ will be shared once analysis and publication of the trial findings has occurred.

For peer review only

REFERENCES

1. Prince M, Wimo A, Guerchet M, Ali G, Wu Y, Prina M. World Alzheimer Report 2015. The global impact of dementia. An analysis of prevalence, incidence, cost & trends; Alzheimer's Disease International: London. 2015.
2. Dauvilliers Y. Insomnia in patients with neurodegenerative conditions. Sleep Medicine. 2007;8:S27-S34.
3. Moran M, Lynch CA, Walsh C, Coen R, Coakley D, Lawlor BA. Sleep disturbance in mild to moderate Alzheimer's disease. Sleep Medicine. 2005;6(4):347-52.
4. de Oliveira AM, Radanovic M, de Mello PC, Buchain PC, Vizzotto AD, Celestino DL, et al. Nonpharmacological Interventions to Reduce Behavioral and Psychological Symptoms of Dementia: A Systematic Review. Biomed Res Int. 2015;2015:218980.
5. Ooms S, Ju Y-E. Treatment of sleep disorders in dementia. Current treatment options in neurology. 2016;18(9):40.
6. Koren T, Fisher E, Webster L, Livingston G, Rapaport P. Prevalence of sleep disturbances in people with dementia living in the community: A systematic review and meta-analysis. Ageing Research Reviews. 2022:101782.
7. Kyle SD, Espie CA, Morgan K. "… Not just a minor thing, it is something major, which stops you from functioning daily": quality of life and daytime functioning in insomnia. Behavioral sleep medicine. 2010;8(3):123-40.
8. Ju Y-ES, Lucey BP, Holtzman DM. Sleep and Alzheimer disease pathology—a bidirectional relationship. Nature reviews Neurology. 2014;10(2):115.
9. Prince M, Knapp M, Guerchet M, McCrone P, Prina M, Comas-Herrera A, et al. Dementia UK: update. London: Alzheimer's Society; 2014.
10. Gehrman P, Gooneratne NS, Brewster GS, Richards KC, Karlawish J. Impact of Alzheimer disease patients' sleep disturbances on their caregivers. Geriatric Nursing. 2018;39(1):60-5.
11. Kinnunen KM, Vikhanova A, Livingston G. The management of sleep disorders in dementia: an update. Curr Opin Psychiatry. 2017;30(6):491-7.
12. Gehrman P, Gooneratne NS, Brewster GS, Richards KC, Karlawish J. Impact of Alzheimer disease patients' sleep disturbances on their caregivers. Geriatric Nursing. 2017.
13. McCurry SM, Logsdon RG, Teri L, Vitiello MV. Sleep disturbances in caregivers of persons with dementia: contributing factors and treatment implications. Sleep medicine reviews. 2007;11(2):143-53.
14. McCrae CS, Dzierzewski JM, McNamara JP, Vatthauer KE, Roth AJ, Rowe MA. Changes in sleep predict changes in affect in older caregivers of individuals with Alzheimer's dementia: a multilevel model approach. Journals of Gerontology Series B: Psychological Sciences and Social Sciences. 2014;71(3):458-62.
15. Wilfling D, Calo S, Dichter MN, Meyer G, Möhler R, Köpke S. Non-pharmacological interventions for sleep disturbances in people with dementia. Cochrane Database of Systematic Reviews. 2023(1).
16. Webster L, Powell K, Costafreda SG, Livingston G. The impact of sleep disturbances on care home residents with dementia: the SIESTA qualitative study. International Psychogeriatrics. 2020;32(7):839-47.
17. Browne J, Edwards DA, Rhodes KM, Brimicombe DJ, Payne RA. Association of comorbidity and health service usage among patients with dementia in the UK: a population-based study. BMJ open. 2017;7(3):e012546.
18. Parliamentary Office of Science and Technology (POST). POSTbrief 29: Sleep and Long-Term Health. UK: Houses of Parliament; 2018.
19. Wang C, Holtzman DM. Bidirectional relationship between sleep and Alzheimer's disease: role of amyloid, tau, and other factors. Neuropsychopharmacology. 2020;45(1):104-20.

For peer review only

20. Cagnin A, Fragiacomo F, Camporese G, Turco M, Bussè C, Ermani M, et al. Sleep-wake profile in dementia with Lewy bodies, Alzheimer's disease, and normal aging. Journal of Alzheimer's Disease. 2017;55(4):1529-36.
21. Mander BA, Winer JR, Jagust WJ, Walker MP. Sleep: A Novel Mechanistic Pathway, Biomarker, and Treatment Target in the Pathology of Alzheimer's Disease? Trends Neurosci. 2016;39(8):552-66.
22. Holth JK, Patel TK, Holtzman DM. Sleep in Alzheimer's Disease–beyond amyloid. Neurobiology of sleep and circadian rhythms. 2017;2:4-14.
23. Livingston G, Sommerlad A, Orgeta V, Costafreda SG, Huntley J, Ames D. Dementia prevention, intervention, and care. 2017;390:2673-734.
24. McCleery J, Sharpley AL. Pharmacotherapies for sleep disturbances in dementia. Cochrane Database of Systematic Reviews. 2020(11).
25. Berry SD, Placide SG, Mostofsky E, Zhang Y, Lipsitz LA, Mittleman MA, et al. Antipsychotic and benzodiazepine drug changes affect acute falls risk differently in the nursing home. Journals of Gerontology Series A: Biomedical Sciences and Medical Sciences. 2015;71(2):273-8.
26. Weich S, Pearce HL, Croft P, Singh S, Crome I, Bashford J, et al. Effect of anxiolytic and hypnotic drug prescriptions on mortality hazards: retrospective cohort study. Bmj. 2014;348:g1996.
27. National Institute for Health and Care Excellence (NICE). Dementia: assessment, management and support for people living with dementia and their carers. NICE guideline (NG97): Methods, evidence and recommendations. . 2018.
28. Dzierzewski JM, O'Brien EM, Kay D, McCrae CS. Tackling sleeplessness: psychological treatment options for insomnia in older adults. Nature and science of sleep. 2010;2:47.
29. Forbes D, Blake CM, Thiessen EJ, Peacock S, Hawranik P. Light therapy for improving cognition, activities of daily living, sleep, challenging behaviour, and psychiatric disturbances in dementia. The Cochrane Library. 2014.
30. Brown CA, Berry R, Tan MC, Khoshia A, Turlapati L, Swedlove F. A critique of the evidence base for non-pharmacological sleep interventions for persons with dementia. Dementia. 2013;12(2):210-37.
31. Livingston G, Barber JA, Kinnunen KM, Webster L, Kyle SD, Cooper C, et al. DREAMS-START (Dementia RElAted Manual for Sleep; STrAtegies for RelaTives) for people with dementia and sleep disturbances: a single-blind feasibility and acceptability randomized controlled trial. International psychogeriatrics. 2018:1-15.
32. Gibson RH, Gander PH, Dowell AC, Jones LM. Non-pharmacological interventions for managing dementia-related sleep problems within community dwelling pairs: A mixed-method approach. Dementia. 2017;16(8):967-84.
33. Tewary S, Cook N, Pandya N, McCurry SM. Pilot test of a six-week group delivery caregiver training program to reduce sleep disturbances among older adults with dementia (Innovative practice). Dementia. 2018;17(2):234-43.
34. Kinnunen KM, Rapaport P, Webster L, Barber J, Kyle SD, Hallam B, et al. A manual-based intervention for carers of people with dementia and sleep disturbances: an acceptability and feasibility RCT. Health Technology Assessment (Winchester, England). 2018;22(71):1-408.
35. Rapaport P, Webster L, Horsley R, Kyle SD, Kinnunen KM, Hallam B, et al. An intervention to improve sleep for people living with dementia: Reflections on the development and co-production of DREAMS:START (Dementia RElAted Manual for Sleep: STrAtegies for RelaTives). Dementia. 2018;17(8):976-89.
36. Montgomery P, Dennis JA. Cognitive behavioural interventions for sleep problems in adults aged 60+. The Cochrane Library. 2003.
37. Sivertsen B, Nordhus IH. Management of insomnia in older adults. The British Journal of Psychiatry. 2007;190(4):285-6.

For peer review only

38. Tractenberg RE, Singer CM, Cummings JL, Thal LJ. The Sleep Disorders Inventory: an instrument for studies of sleep disturbance in persons with Alzheimer's disease. Journal of sleep research. 2003;12(4):331-7.
39. Cape J, Leibowitz J, Whittington C, Espie C, Pilling S. Group cognitive behavioural treatment for insomnia in primary care: a randomized controlled trial. Psychological medicine. 2016;46(5):1015-25.
40. Livingston G, Barber J, Rapaport P, Knapp M, Griffin M, King D, et al. Clinical effectiveness of a manual based coping strategy programme (START, STrAtegies for RelaTives) in promoting the mental health of carers of family members with dementia: pragmatic randomised controlled trial. BMJ. 2013;347:f6276.
41. Livingston G, Barber J, Rapaport P, Knapp M, Griffin M, King D, et al. Long-term clinical and cost-effectiveness of psychological intervention for family carers of people with dementia: a single-blind, randomised, controlled trial. Lancet Psychiatry. 2014;1(7):539-48.
42. Juul S, Gluud C, Simonsen S, Frandsen FW, Kirsch I, Jakobsen JC. Blinding in randomised clinical trials of psychological interventions: a retrospective study of published trial reports. BMJ Evidence-Based Medicine. 2021;26(3):109-.
43. Livingston G, Barber J, Marston L, Stringer A, Panca M, Hunter R, et al. Clinical and cost-effectiveness of the Managing Agitation and Raising Quality of Life (MARQUE) intervention for agitation in people with dementia in care homes: a single-blind, cluster-randomised controlled trial. The Lancet Psychiatry. 2019.
44. McCurry SM, Pike KC, Vitiello MV, Logsdon RG, Larson EB, Teri L. Increasing walking and bright light exposure to improve sleep in community-dwelling persons with Alzheimer's Disease: results of a randomized, controlled trial. Journal of the American Geriatrics Society. 2011;59(8):1393-402.
45. Kinnunen K, P R, L W, J B, D KS, B H, et al. A manual-based intervention for carers of people with dementia and sleep disturbances: an acceptability and feasibility RCT. . Health Technol Assess. 2018;22(71).
46. Cummings JL. The Neuropsychiatric Inventory: assessing psychopathology in dementia patients. Neurology. 1997;48(5 Suppl 6):S10-6.
47. Johns MW. Sleepiness in different situations measured by the Epworth Sleepiness Scale. Sleep. 1994;17(8):703-10.
48. Smith SC, Lamping DL, Banerjee S, Harwood RH, Foley B, Smith P. Development of a new measure of health-related quality of life for people with dementia: DEMQOL. 2007;37:737-46.
49. Beecham J, Knapp M. Costing Psychiatric Interventions. In: Thornicroft G, Brewin CR, Wing J, editors. Measuring Mental Health Needs. London: Gaskell/Royal College of Psychiatrists; 1992.
50. Bryan S, Hardyman W, Bentham P, Buckley A, Laight A. Proxy completion of EQ-5D in patients with dementia. Quality of Life Research. 2005;14(1):107-18.
51. Clarke CL, Taylor J, Crighton LJ, Goodbrand JA, McMurdo ME, Witham MD. Validation of the AX3 triaxial accelerometer in older functionally impaired people. Aging clinical and experimental research. 2017;29(3):451-7.
52. Espie CA, Kyle SD, Hames P, Gardani M, Fleming L, Cape J. The Sleep Condition Indicator: a clinical screening tool to evaluate insomnia disorder. BMJ open. 2014;4(3):e004183.
53. Zigmond AS, Snaith RP. The hospital anxiety and depression scale. Acta psychiatrica scandinavica. 1983;67(6):361-70.
54. Zarit SH, Reever KE, Bach-Peterson J. Relatives of the impaired elderly: correlates of feelings of burden. Gerontologist. 1980;20(6):649-55.
55. Barry TL, Kaiser KL, Atwood JR. Reliability, validity, and scoring of the Health Status Questionnaire-12 version 2.0. J Nurs Meas. 2007;15(1):24-35.
56. Randles R, Wolfe D. Introduction to the theory of nonparametric statistics. : Krieger; 1991.

For peer review only

57. Walwyn R, Roberts C. Therapist variation within randomised trials of psychotherapy: implications for precision, internal and external validity. Statistical Methods in Medical Research. 2010;19(3):291-315.

58. Cro S, Morris TP, Kenward MG, Carpenter JR. Sensitivity analysis for clinical trials with missing continuous outcome data using controlled multiple imputation: A practical guide. Statistics in Medicine. 2020;39(21):2815-42.

59. Skivington K, Matthews L, Simpson SA, Craig P, Baird J, Blazeby JM, et al. A new framework for developing and evaluating complex interventions: update of Medical Research Council guidance. BMJ. 2021;374:n2061.

60. Braun V, Clarke V. Using thematic analysis in psychology. Qualitative Research in Psychology. 2006;3(2):77-101.

61. French SD, Green SE, O'Connor DA, McKenzie JE, Francis JJ, Michie S, et al. Developing theory-informed behaviour change interventions to implement evidence into practice: a systematic approach using the Theoretical Domains Framework. Implementation Science. 2012;7.

62. Whittle R, Mansell G, Jellema P, van der Windt D. Applying causal mediation methods to clinical trial data: What can we learn about why our interventions (don't) work? European Journal of Pain. 2017;21(4):614-22.

63. Noell GG, FM; Gansle, K. Does treatment integrity matter? A preliminary investigation of instructional implementation and mathematical performance. Journal of Behavioral Education 2002;11:15.

For peer review only

1 Figure Legends:
2 Figure 1 - DREAMS START RCT flow diagram
3 Figure 2 – Draft logic model

For peer review only

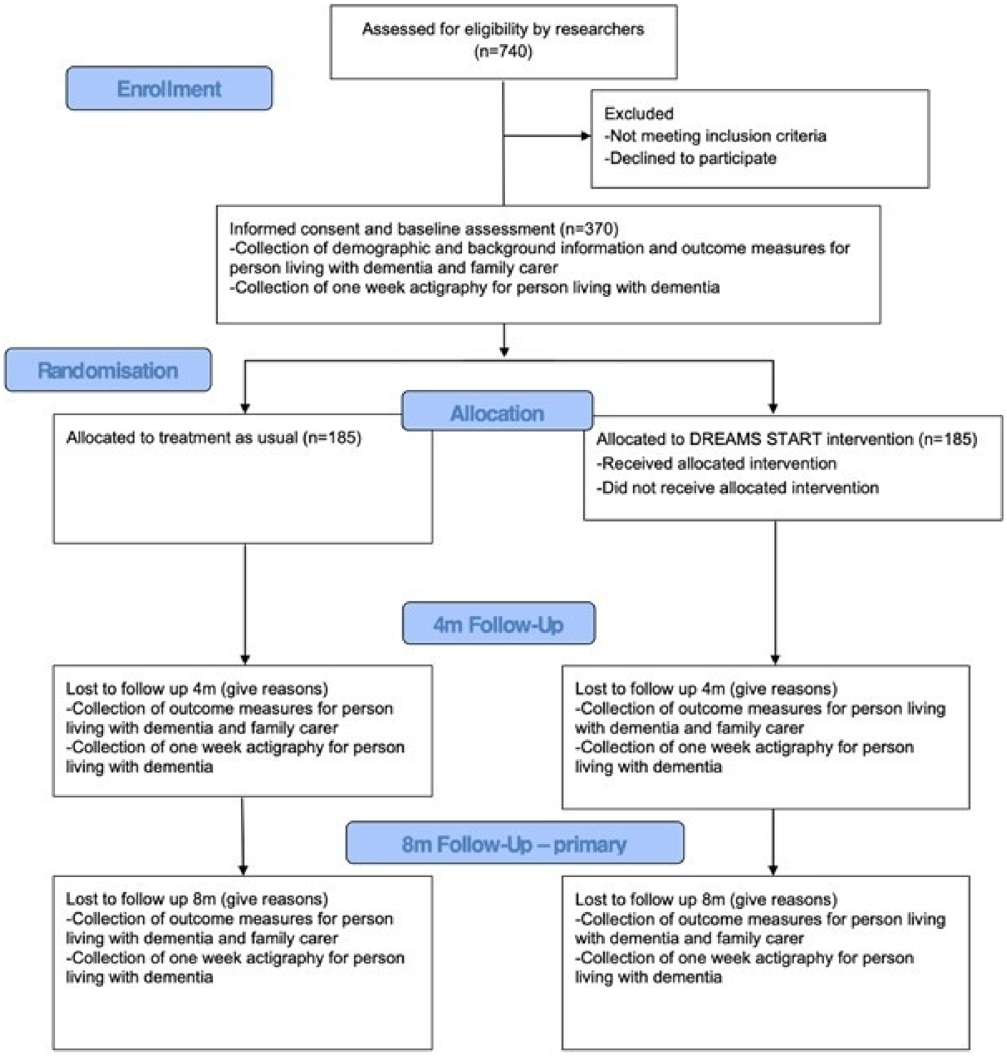

DREAMS START RCT flow diagram

50x56mm (300 x 300 DPI)

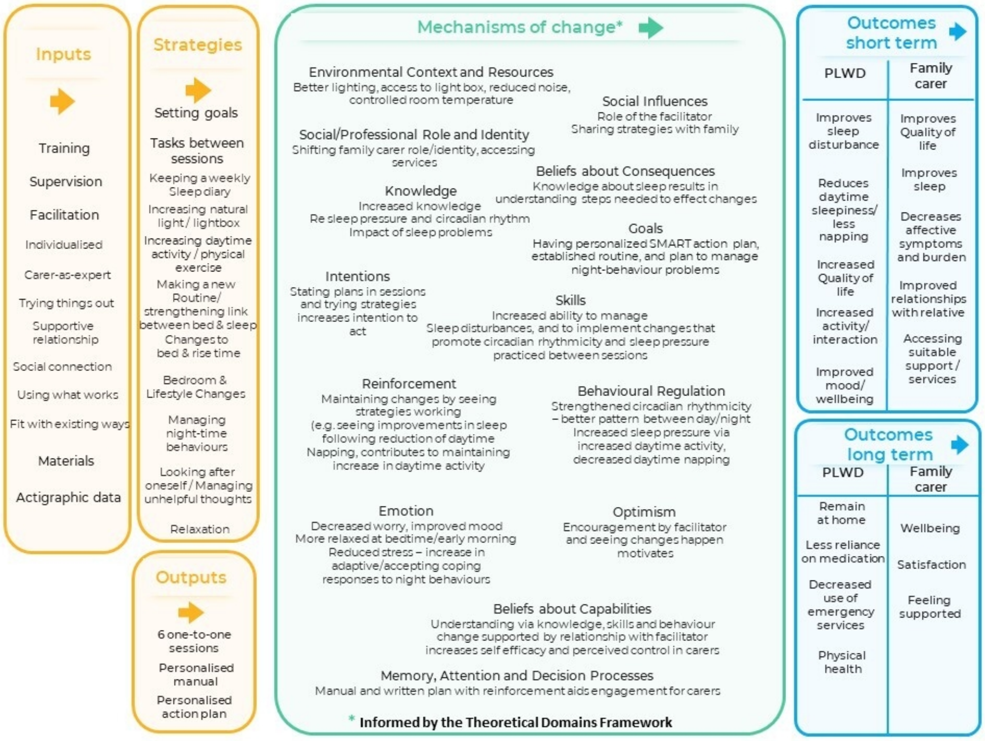

Draft logic model

81x60mm (300 x 300 DPI)

Supplementary Table 1 Schedule of visits and assessments

	Screening / Baseline	Randomisation	Session 1	Session 2	Session 3	Session 4	Session 5	Session 6	4mth follow up	8mth follow up	+ 8month
Informed Consent	x										
Sleep disorders inventory	x								x	x	
Eligibility confirmation	x										
Demographics (person with dementia)	x										
Neuropsychiatric Inventory	x								x	x	
Epworth Sleepiness Scale	x								x	x	
DEMQOL-Proxy	x								x	x	
CSRI/medication (proxy, for person with dementia)	x								x	x	
EQ-5D-5L (proxy, for person with dementia)	x								x	x	
Side effects	x								x	x	
Actigraphy	x								x	x	
Demographics (carer)	x										
Sleep Condition Indicator (carer)	x										

For peer review only

Hospital Anxiety and Depression Scale (carer)	x								x	x	
Zarit Burden Interview (carer)	x								x	x	
Health Status Questionnaire (12) (carer)	x								x	x	
CSRI/medication (carer)	x								x	x	
EQ-5D-5L (carer)	x								x	x	
Randomisation		x									
DREAMS START intervention			x	x	x	x	x	x			
Intervention acceptability (qualitative interview)											x

For peer review only

Appendix 1 Informed consent forms

**Site/Centre Number:**
**Dyad Number:**
**Participant Identification Number:**

[INSERT NHS TRUST LOGO HERE]

FAMILY CARER CONSENT FORM

Title: DREAMS START: Dementia-related manual for sleep strategies for relatives

Name of Researcher:

Please initial box

1. I confirm that I have read the information sheet dated 20-DEC-2020 (version 2.1) for the above study and understand the purpose of the research and I have received a copy of the information sheet and informed consent form to keep
2. I have had the opportunity to consider the information, ask questions and have had these answered satisfactorily
3. I understand that my participation is voluntary and that I am free to withdraw from the research study at any time without my legal rights or medical care being affected.
4. I understand that that my personal information will be processed only for the purposes of research and that all material and personal information will be kept in accordance with General Data Protection Regulation (GDPR) and the Data Protection Act 2018.
5. I understand that relevant data collected during the study may be looked at by individuals from the sponsor of the trial (Camden & Islington NHS Foundation Trust), UCL, regulatory authorities or from the NHS Trust, where it is relevant to my taking part in this research. I give permission for these individuals to have access to this data
6. I understand that a UCL-approved transcription company will have temporary access to the information I provide in the audio recordings and I agree for my sessions and interview with the researcher to be audiotaped *(Optional)*
7. I agree to be contacted again for future research studies *(Optional)*
8. I understand that the information I share, including anonymised direct quotes, may be included in any resulting report, publications and conference presentations *(Optional)*
9. I agree that the information collected about me can be used to support other research in the future, and may be shared anonymously with other researchers *(Optional)*
10. I would like to receive a summary of the research findings *(Optional)*
11. I agree to take part in the above study
12. I agree to share my mobile phone number to receive text reminders as part of the intervention and for my mobile number only to be transferred to a UCL-approved text messaging service

Name of Participant	Date	Signature
Name of Person taking consent	Date	Signature

DREAMS START FC_CONSENT_IRAS 272935_v2.1_20-DEC-2020 Page 1 of 1

Site/Centre Number:

Dyad Number:

Participant Identification Number:

[INSERT NHS TRUST LOGO HERE]

CONSULTEE DECLARATION FORM

Title: DREAMS START: Dementia-related manual for sleep strategies for relatives

Please initial box

1. I [insert name] have been consulted about [insert name of participant]'s participation in this research project. I confirm that I have read the information sheet dated 20-DEC-2020 (version 2.1) for the above study and understand the purpose of the research and I have received a copy of the information sheet and consultee declaration form to keep.
2. I have had the opportunity to consider the information, ask questions and have had these answered satisfactorily.
3. I understand that his/her participation is voluntary and that I am free to request that they are withdrawn from the study at any time without their legal or medical rights being affected.
4. I understand that his/her personal information will be processed only for the purposes of research and that all material and personal information will be kept in accordance with General Data Protection Regulation (GDPR) and the Data Protection Act 2018.
5. I understand that relevant data collected during the study may be looked at by individuals from the sponsor of the trial (Camden & Islington NHS Foundation Trust), UCL, regulatory authorities or from the NHS Trust, where it is relevant to their taking part in this research. I give permission for these individuals to have access to this data.
6. I agree for his/her GP being informed of their participation in the study, including any necessary exchange of information about him/her between their GP and the research team
7. I understand that an external UCL-approved transcription company will have temporary access to the information he/she provides in the audio recording and I agree for their sessions and interview with the researcher to be audiotaped. *(optional)*
8. I agree to he/she being contacted again for future research studies *(optional)*
9. I understand that the information he/shares, including anonymised direct quotes, may be included in any resulting report, publications and conference presentations *(optional)*
10. I understand that the information collected regarding he/she will be used to support other research in the future, and may be shared anonymously with other researchers *(optional)*
11. He/she would like to receive a summary of the research findings *(optional)*
12. In my opinion he/she would have no objection to taking part in the above study.

Name of Consultee	Relationship to participant	Date	Signature

Name of Person taking consent	Date	Signature

Site/Centre Number:

Dyad Number:

Participant Identification Number:

[INSERT TRUST LOGO HERE]

PERSON LIVING WITH DEMENTIA CONSENT FORM

Title: DREAMS START: Dementia-related manual for sleep strategies for relatives

Please initial box

1. I confirm that I have read the information sheet dated 20-DEC-2020 (version 2.1) for the above study and understand the purpose of the research and I have received a copy of the information sheet and consent form to keep.
2. I have had the opportunity to consider the information, ask questions and have had these answered satisfactorily
3. I understand that my participation is voluntary and that I am free to withdraw from the research study at any time without my legal rights or medical care being affected.
4. I agree that if I lose capacity to consent during the study, I would/I would not be willing for my relative to act as a personal legal representative and give consent on my behalf
5. I understand that that my personal information will be processed only for the purposes of research and that all material and personal information will be kept in accordance with General Data Protection Regulation (GDPR) and the Data Protection Act 2018.
6. I understand that relevant data collected during the study may be looked at by individuals from the sponsor of the trial (Camden & Islington NHS Foundation Trust), UCL, regulatory authorities or from the NHS Trust, where it is relevant to my taking part in this research. I give permission for these individuals to have access to this data.
7. I agree to my General Practitioner being informed of my participation in the study, including any necessary exchange of information about me between my GP and the research team
8. I understand that an external UCL-approved transcription company will have temporary access to the information I provide in the audio recordings I agree for my sessions and interview with the researcher to be audiotaped *(optional)*
9. I agree to be contacted again for future research studies *(optional)*
10. I understand that the information I share, including anonymised direct quotes, may be included in any resulting report, publications and conference presentations *(optional)*
11. I agree that the information collected about me can be used to support other research in the future, and may be shared anonymously with other researchers *(optional)*
12. I would like to receive a summary of the research findings *(optional)*
13. I agree to take part in the above study

Name of Participant	Date	Signature
Name of Person taking consent	Date	Signature

For peer review only - http://bmjopen.bmj.com/site/about/guidelines.xhtml

Appendix 2 Intervention components

Session/Section	Derived from
**Session 1**	
Sleep and dementia –	material provided by Simon D Kyle.
What is sleep? –	material provided by Simon D Kyle.
What causes sleep problems in dementia? –	written by Penny Rapaport, Simon D Kyle and Gill Livingston for DREAMS.
Making changes to improve sleep –	adapted from cognitive–behavioural therapy (CBT) work by Colin A Espie.
The impact of sleep problems on you and our relative	developed for DREAMS.
Managing the stress that sleep problems can bring –	adapted from START*.
Managing stress: the signal breath –	adapted from START.
Summary –	adapted from START.
Putting it into practice –	adapted from START.
**Session 2**	
Recap on understanding sleep and dementia.	
Establishing a good day and night routine	adapted from CBT work by Colin A Espie.
Your relative's sleep pattern –	developed for DREAMS.
Light and sleep –	material provided by Simon D Kyle.
Light, dementia and the body clock –	material provided by Simon D Kyle.
Making a light therapy plan –	developed for DREAMS.
Making a new sleep routine: your relative's plan –	developed for DREAMS based on work on sleep efficiency by Colin A Espie and Simon D Kyle.
Managing stress 2: focused breathing –	adapted from START.
Summary –	adapted from START.
Putting it into practice –	adapted from START.
**Session 3**	
Recap on making a plan.	
The importance of daytime activity and routine –	adapted from START.
Planning daytime activity –	adapted from START.

For peer review only

Sleep, exercise and physical activity –	developed for DREAMS by Penny Rapaport, Gill Livingston and Simon D Kyle.
Managing stress 3: guided imagery –	adapted from START.
Summary –	adapted from START.
Putting it into practice –	adapted from START.
Seated exercises visual guide –	from NHS Choices website.66
**Session 4**	
Recap on daytime activity and routine.	
Troubleshooting: putting plans into action –	developed for DREAMS.
Your relative's plan	developed for DREAMS.
Managing night-time behaviour problems –	adapted from MARQUE**/START.
Describing and investigating behaviours –	adapted from MARQUE/START.
Managing stress 4: stretching –	adapted from START.
Summary –	adapted from START.
Putting it into practice –	adapted from START.
**Session 5**	
Recap on night-time behaviour problems.	
Creating strategies for managing behaviours –	adapted from MARQUE.
Managing your own sleep –	developed for DREAMS.
Managing thoughts and feelings –	adapted from CBT work by Colin A Espie.
Challenging unhelpful thoughts and feelings –	adapted from START.
Making time for yourself	adapted from START.
Managing stress 5: guided imagery – ocean escape –	adapted from START.
Summary –	adapted from START.
Putting it into practice –	adapted from START.
**Session 6**	**Overall structure based on that developed in START and refined in MARQUE.**
Putting it all together.	
What works? Light, sleep and dementia –	written by Simon D Kyle for DREAMS.
What works? The importance of daytime activity –	adapted from START.

For peer review only

What works? Making a new sleep routine –	based on the sleep manual by Colin A Espie and Simon D Kyle.
What works? Making changes to improve sleep –	based on the sleep manual by Colin A Espie and Simon D Kyle.
What works? Managing night-time behaviours –	based on MARQUE/START.
What works? Challenging unhelpful thoughts and feelings –	based on START and CBT work by Colin A Espie.
What works? Relaxation –	adapted from START.
Keeping it going – developing an action plan –	developed for DREAMS.
Action plan for you and your relative –	developed for DREAMS.
Summary –	developed for DREAMS.

* STrAtegies for RelaTives (START) for further information see https://www.thelancet.com/journals/lanpsy/article/PIIS2215-0366(14)00073-X/fulltext

** Managing Agitation and Raising Quality of Life (MARQUE) for further information see https://pubmed.ncbi.nlm.nih.gov/30872010/

For peer review only

Reporting checklist for protocol of a clinical trial.

Based on the SPIRIT guidelines.

Instructions to authors

Complete this checklist by entering the page numbers from your manuscript where readers will find each of the items listed below.

Your article may not currently address all the items on the checklist. Please modify your text to include the missing information. If you are certain that an item does not apply, please write "n/a" and provide a short explanation.

Upload your completed checklist as an extra file when you submit to a journal.

In your methods section, say that you used the SPIRITreporting guidelines, and cite them as:

Chan A-W, Tetzlaff JM, Gøtzsche PC, Altman DG, Mann H, Berlin J, Dickersin K, Hróbjartsson A, Schulz KF, Parulekar WR, Krleža-Jerić K, Laupacis A, Moher D. SPIRIT 2013 Explanation and Elaboration: Guidance for protocols of clinical trials. BMJ. 2013;346:e7586

		Reporting Item	Page Number (in manuscript)	Page Number (in full protocol 13-JAN-2023 Version 4 available for download https://doi.org/10.1186/ISRCTN13072268)
**Administrative information**				

For peer review only

Title	#1	Descriptive title identifying the study design, population, interventions, and, if applicable, trial acronym	1	
Trial registration	#2a	Trial identifier and registry name. If not yet registered, name of intended registry	3	
Trial registration: data set	#2b	All items from the World Health Organization Trial Registration Data Set	18	
Protocol version	#3	Date and version identifier	13-JAN-2023 Version 4	
Funding	#4	Sources and types of financial, material, and other support	17	

For peer review only

Roles and responsibilities: contributorship	#5a	Names, affiliations, and roles of protocol contributors	17	
Roles and responsibilities: sponsor contact information	#5b	Name and contact information for the trial sponsor		1
Roles and responsibilities: sponsor and funder	#5c	Role of study sponsor and funders, if any, in study design; collection, management, analysis, and interpretation of data; writing of the report; and the decision to submit the report for publication, including whether they will have ultimate authority	n/a	

For peer review only

		over any of these activities		
Roles and responsibilities: committees	#5d	Composition, roles, and responsibilities of the coordinating centre, steering committee, endpoint adjudication committee, data management team, and other individuals or groups overseeing the trial, if applicable (see Item 21a for data monitoring committee)	7	50
**Introduction**				
Background and rationale	#6a	Description of research question and justification for	4, 5	

For peer review only

		undertaking the trial, including summary of relevant studies (published and unpublished) examining benefits and harms for each intervention		
Background and rationale: choice of comparators	#6b	Explanation for choice of comparators	6	
Objectives	#7	Specific objectives or hypotheses	6	
Trial design	#8	Description of trial design including type of trial (eg, parallel group, crossover, factorial, single group), allocation ratio, and	6	

For peer review only

		framework (eg, superiority, equivalence, non-inferiority, exploratory)		
**Methods: Participants, interventions, and outcomes**				
Study setting	#9	Description of study settings (eg, community clinic, academic hospital) and list of countries where data will be collected. Reference to where list of study sites can be obtained	6	
Eligibility criteria	#10	Inclusion and exclusion criteria for participants. If applicable,	7, 8	

For peer review only

		eligibility criteria for study centres and individuals who will perform the interventions (eg, surgeons, psychotherapists)		
Interventions: description	#11a	Interventions for each group with sufficient detail to allow replication, including how and when they will be administered	8, 9	
Interventions: modifications	#11b	Criteria for discontinuing or modifying allocated interventions for a given trial participant (eg, drug dose change in response to	n/a (non-CTIMPS)	

For peer review only

		harms, participant request, or improving / worsening disease)		
Interventions: adherance	#11c	Strategies to improve adherence to intervention protocols, and any procedures for monitoring adherence (eg, drug tablet return; laboratory tests)	8	
Interventions: concomitant care	#11d	Relevant concomitant care and interventions that are permitted or prohibited during the trial	9	
Outcomes	#12	Primary, secondary, and	10, 11	

For peer review only

		other outcomes, including the specific measurement variable (eg, systolic blood pressure), analysis metric (eg, change from baseline, final value, time to event), method of aggregation (eg, median, proportion), and time point for each outcome. Explanation of the clinical relevance of chosen efficacy and harm outcomes is strongly recommended		

For peer review only

Participant timeline	#13	Time schedule of enrolment, interventions (including any run-ins and washouts), assessments, and visits for participants. A schematic diagram is highly recommended (see Figure)	See table 1, page 25, 26	
Sample size	#14	Estimated number of participants needed to achieve study objectives and how it was determined, including clinical and statistical assumptions supporting any	11	

For peer review only

		sample size calculations		
Recruitment	#15	Strategies for achieving adequate participant enrolment to reach target sample size		48, 49
**Methods: Assignment of interventions (for controlled trials)**				
Allocation: sequence generation	#16a	Method of generating the allocation sequence (eg, computer-generated random numbers), and list of any factors for stratification. To reduce	9, 10	

For peer review only

		predictability of a random sequence, details of any planned restriction (eg, blocking) should be provided in a separate document that is unavailable to those who enrol participants or assign interventions		
Allocation concealment mechanism	#16b	Mechanism of implementing the allocation sequence (eg, central telephone; sequentially numbered, opaque, sealed envelopes), describing any steps to conceal	9, 10	

For peer review only

		the sequence until interventions are assigned		
Allocation: implementation	#16c	Who will generate the allocation sequence, who will enrol participants, and who will assign participants to interventions	9, 10	
Blinding (masking)	#17a	Who will be blinded after assignment to interventions (eg, trial participants, care providers, outcome assessors, data analysts), and how	9, 10	
Blinding (masking):	#17b	If blinded, circumstances under which	n/a	

For peer review only

emergency unblinding		unblinding is permissible, and procedure for revealing a participant's allocated intervention during the trial		
**Methods: Data collection, management, and analysis**				
Data collection plan	#18a	Plans for assessment and collection of outcome, baseline, and other trial data, including any related processes to promote data quality (eg, duplicate measurements,	10, 11	

For peer review only

		training of assessors) and a description of study instruments (eg, questionnaires, laboratory tests) along with their reliability and validity, if known. Reference to where data collection forms can be found, if not in the protocol		
Data collection plan: retention	#18b	Plans to promote participant retention and complete follow-up, including list of any outcome data to be collected for participants who discontinue or	10, 11	

For peer review only

		deviate from intervention protocols		
Data management	#19	Plans for data entry, coding, security, and storage, including any related processes to promote data quality (eg, double data entry; range checks for data values). Reference to where details of data management procedures can be found, if not in the protocol		40-42
Statistics: outcomes	#20a	Statistical methods for	12, 13, 14	

For peer review only

		analysing primary and secondary outcomes. Reference to where other details of the statistical analysis plan can be found, if not in the protocol		
Statistics: additional analyses	#20b	Methods for any additional analyses (eg, subgroup and adjusted analyses)	13, 14	
Statistics: analysis population and missing data	#20c	Definition of analysis population relating to protocol non-adherence (eg, as randomised analysis), and any statistical	13, 14	

For peer review only

		methods to handle missing data (eg, multiple imputation)		
**Methods: Monitoring**				
Data monitoring: formal committee	#21a	Composition of data monitoring committee (DMC); summary of its role and reporting structure; statement of whether it is independent from the sponsor and competing interests; and reference to where further details about its charter can be found, if not in the protocol.		50

For peer review only

		Alternatively, an explanation of why a DMC is not needed		
Data monitoring: interim analysis	#21b	Description of any interim analyses and stopping guidelines, including who will have access to these interim results and make the final decision to terminate the trial		35
Harms	#22	Plans for collecting, assessing, reporting, and managing solicited and spontaneously reported adverse events and other	12	

For peer review only

		unintended effects of trial interventions or trial conduct		
Auditing	#23	Frequency and procedures for auditing trial conduct, if any, and whether the process will be independent from investigators and the sponsor		52
**Ethics and dissemination**				
Research ethics approval	#24	Plans for seeking research ethics committee / institutional review board (REC / IRB) approval	3	
Protocol amendments	#25	Plans for communicating		51

For peer review only

		important protocol modifications (eg, changes to eligibility criteria, outcomes, analyses) to relevant parties (eg, investigators, REC / IRBs, trial participants, trial registries, journals, regulators)		
Consent or assent	#26a	Who will obtain informed consent or assent from potential trial participants or authorised surrogates, and how (see Item 32)	7, 8	

For peer review only

Consent or assent: ancillary studies	#26b	Additional consent provisions for collection and use of participant data and biological specimens in ancillary studies, if applicable	n/a	
Confidentiality	#27	How personal information about potential and enrolled participants will be collected, shared, and maintained in order to protect confidentiality before, during, and after the trial		
Declaration of interests	#28	Financial and other competing interests for	17	

For peer review only

		principal investigators for the overall trial and each study site		
Data access	#29	Statement of who will have access to the final trial dataset, and disclosure of contractual agreements that limit such access for investigators		42
Ancillary and post trial care	#30	Provisions, if any, for ancillary and post-trial care, and for compensation to those who suffer harm from trial participation	n/a	
Dissemination policy: trial results	#31a	Plans for investigators and sponsor to		52, 53

For peer review only

		communicate trial results to participants, healthcare professionals, the public, and other relevant groups (eg, via publication, reporting in results databases, or other data sharing arrangements), including any publication restrictions		
Dissemination policy: authorship	#31b	Authorship eligibility guidelines and any intended use of professional writers		52, 53

For peer review only

Dissemination policy: reproducible research	#31c	Plans, if any, for granting public access to the full protocol, participant-level dataset, and statistical code	18	
**Appendices**				
Informed consent materials	#32	Model consent form and other related documentation given to participants and authorised surrogates	Supplemental material	
Biological specimens	#33	Plans for collection, laboratory evaluation, and storage of biological specimens for genetic or	n/a	

For peer review only

		molecular analysis in the current trial and for future use in ancillary studies, if applicable		

None The SPIRIT Explanation and Elaboration paper is distributed under the terms of the Creative Commons Attribution License CC-BY-NC. This checklist can be completed online using https://www.goodreports.org/, a tool made by the EQUATOR Network in collaboration with Penelope.ai

For peer review only

The TIDieR (Template for Intervention Description and Replication) Checklist*:

Number	Item	Detailed description	Where located **	
			Protocol paper (page or appendix number)	Other † (details)
**1.**	**BRIEF NAME**<br>Provide the name or a phrase that describes the intervention.	DREAMS START (Dementia Related Manual for Sleep: Strategies for Relatives)	Page.1 line 1	
**2.**	**WHY**<br>Describe any rationale, theory, or goal of the elements essential to the intervention.	The co-designed intervention supports carers to develop strategies to improve their relative with dementia's sleep. DREAMS START provides information about sleep and dementia, supports carers to use practical *zeitgebers* (cues that influence the person's biological rhythms e.g. regular timing of bed and rising, morning wake-up light, regular meal times) and to establish adaptive stimulus control (e.g. pre-bed settling routine, management of wakeful episodes). It uses strategies to promote de-arousal at night (e.g. relaxation, bedroom comfort, no caffeine or alcohol pre-bed, relaxation) and daytime behavioural activation to maintain alertness and reduce daytime naps. The rationale for this is that it will strengthen the natural	Appendix 2 (Intervention components)<br><br>Page.5 line 20-32<br><br>Page 8 lines 14-30	Rapaport P et al: Dementia. 2018;17(8):976-89.<br><br>Full protocol at https://doi.org/10.1186/ISRCTN13072268

For peer review only - http://bmjopen.bmj.com/site/about/guidelines.xhtml

		circadian rhythmicity and increase sleep pressure in the person living with dementia. The intervention also focuses on helping carers to look after their own (sleep) health and to use behavioural strategies for reducing distress behaviours at night. Each session teaches different relaxation strategies. The rationale for this is that family carers will be able to reduce the impact of the sleep disturbance on their own sleep and having a greater understanding and acceptance of the situation may reduce their own distress.		
**3.**	**WHAT**<br>Materials: Describe any physical or informational materials used in the intervention, including those provided to participants or used in intervention delivery or in training of intervention providers. Provide information on where the materials can be accessed (e.g. online appendix, URL).	The participants receive a paper copy of the DREAMS START manual that they can write in with personalised strategies, sleep diaries and record forms and an individual action plan (once the trial is complete the manuals will be made available online). The participants are also provided with a phototherapy light box and access to recordings of relaxation exercises. If their relative has worn an actigraph prior to the intervention they are given a summary of their relatives sleep pattern with an interpretation alongside their manual.	Page 8 lines 14-30	Full protocol at https://doi.org/10.1186/ISRCTN13072268
**4.**	Procedures: Describe each of the procedures, activities, and/or processes used in the	DREAMS START is delivered over six sessions (see below for mode of delivery). The intervention is delivered by a facilitator who is trained in therapeutic engagement skills and in how to support	Abstract<br><br>Page 8 lines 14-	Full protocol at https://doi.org/10.1186/ISRCTN

For peer review only - http://bmjopen.bmj.com/site/about/guidelines.xhtml

	intervention, including any enabling or support activities.	participants (family carers) to implement and change behaviours in themselves and their relatives. Each session includes practical exercises to develop tailored strategies, between session monitoring and practice tasks, reflection on the previous session and building an individual and written action plan, adding strategies each week. Participants are encouraged to share ideas and strategies with their wider support network and facilitators are encouraged to build on the carer's existing skills and expertise, focusing on what works well and validating challenging or distressing experiences.	30	13072268
**5.**	**WHO PROVIDED** For each category of intervention provider (e.g. psychologist, nursing assistant), describe their expertise, background and any specific training given.	The intervention facilitators are non-clinically qualified psychology graduates. They will receive a two-day training which will focus on dementia and sleep-wake regulation, using actigraphs, empathic listening skills, facilitating behaviour change, using supervision effectively and working collaboratively with family carers and people living with dementia. It will emphasise the need to operate from an inclusive values base and to respect diversity and the existing knowledge and skills of family carers. Facilitators will learn through a combination of seminars, discussion, reflective learning, and guided reading. Facilitators will have fortnightly group supervision with a clinical psychologist with additional individual supervision as requested by facilitators or the study team. The group supervision format makes most effective use of available resources and enables	Page 8 line 32-page 9 line 13	Full protocol at https://doi.org/10.1186/ISRCTN13072268

For peer review only - http://bmjopen.bmj.com/site/about/guidelines.xhtml

		the facilitators to benefit from the professional expertise of their supervisor and the experiences of their peers. Supervision will include case management, skills development, risk management and safe practice and staff support.		
6.	**HOW** Describe the modes of delivery (e.g. face-to-face or by some other mechanism, such as internet or telephone) of the intervention and whether it was provided individually or in a group.	The intervention will be delivered flexibly depending on the preference and practical considerations for the participants and facilitators. DREAMS START can be delivered face-to-face, by video conferencing or by telephone, or in a combination of these. Participants will always have a copy of the manual with them for the sessions as well as being guided by the facilitator. The sessions will be delivered to family carers individually unless the person living with dementia wishes (and is able) to participate and other family members or paid care staff can join sessions if that is felt to be useful.	Page 8 lines 29-30	Full protocol at https://doi.org/10.1186/ISRCTN13072268
7.	**WHERE** Describe the type(s) of location(s) where the intervention occurred, including any necessary infrastructure or relevant features.	DREAMS START will be delivered in people's homes if face to face (unless participants would prefer to be seen at a clinic or university building for convenience).	Page 8 lines 29-30	Full protocol at https://doi.org/10.1186/ISRCTN13072268
	**WHEN AND HOW MUCH**			

For peer review only - http://bmjopen.bmj.com/site/about/guidelines.xhtml

**8.**	Describe the number of times the intervention was delivered and over what period of time including the number of sessions, their schedule, and their duration, intensity or dose.	The intervention is delivered over six-hour long sessions. This is delivered flexibly with a session every one to two weeks depending on family carer availability.	Abstract<br><br>Page 8 lines 14-30	Full protocol at https://doi.org/10.1186/ISRCTN13072268
	**TAILORING**			
**9.**	If the intervention was planned to be personalised, titrated or adapted, then describe what, why, when, and how.	The intervention is planned to be tailored by the facilitator working with the participant to identify and prioritise specific concerns, develop personalised strategies and note these in the manual and then develop a smart action plan. The individual sleep data from actigraphy can be used by the facilitator to make specific recommendations around time to bed and rise and other actions such as exercise or steps to reduce daytime sleep.	Page 8 lines 14-30	Full protocol at https://doi.org/10.1186/ISRCTN13072268
	**MODIFICATIONS**			
**10.‡**	If the intervention was modified during the course of the study, describe the changes (what, why, when, and how).	Since the intervention was feasible and acceptable during our initial feasibility RCT, with carers responding positively in our qualitative study, we did not make major changes prior to the full RCT. The main change we made was that during the feasibility trial we did deliver the intervention to people living with dementia living alone without a carer present and found that this was impractical and challenging for participants and facilitators. We made minor revisions	Page 8 lines 29-30	Livingston et al (2018) International Psychogeriatrics 2019;31(2):251-265.

For peer review only - http://bmjopen.bmj.com/site/about/guidelines.xhtml

		are based on feedback from family carers for example adding further detail on use of assistive technology and adapted our record forms to make them simpler and easier to use. As the Covid 19 pandemic occurred between the feasibility and full RCT we also adapted the mode of delivery (see question 6 above) away from a fully face-to-face model.		
**11.**	**HOW WELL**<br>Planned: If intervention adherence or fidelity was assessed, describe how and by whom, and if any strategies were used to maintain or improve fidelity, describe them.	To improve fidelity we have a manualised intervention, training and supervision of facilitators (see question 5) and to ensure treatment integrity, facilitators are required to demonstrate competence in delivering the intervention by role-play before we begin recruitment with one of the study clinician co-applicants. We will collect information on trial adherence and attendance. In addition to supervision and training, we will formally monitor fidelity to the intervention. Facilitators will, with participant's permission, record one intervention session per participant, decided by the trial manager randomly generating session numbers. A researcher not involved in the therapy will independently rate fidelity to the manual, using our existing checklist incorporating the key content of each session and four process factors ("keeping the session to time", "keeping the carer focused on the manual", "keeping the carer engaged in the session" and "managing the concerns of the carer").	Page 8 line 32 – page 9 line13	Full protocol at https://doi.org/10.1186/ISRCTN13072268
**12.**[‡]	Actual: If intervention	N/A as this is protocol.	N/A	

For peer review only - http://bmjopen.bmj.com/site/about/guidelines.xhtml

	adherence or fidelity was assessed, describe the extent to which the intervention was delivered as planned.			

** **Authors** - use N/A if an item is not applicable for the intervention being described. **Reviewers** – use '?' if information about the element is not reported/not sufficiently reported.

† If the information is not provided in the primary paper, give details of where this information is available. This may include locations such as a published protocol or other published papers (provide citation details) or a website (provide the URL).
‡ If completing the TIDieR checklist for a protocol, these items are not relevant to the protocol and cannot be described until the study is complete.

* We strongly recommend using this checklist in conjunction with the TIDieR guide (see *BMJ* 2014;348:g1687) which contains an explanation and elaboration for each item.

* The focus of TIDieR is on reporting details of the intervention elements (and where relevant, comparison elements) of a study. Other elements and methodological features of studies are covered by other reporting statements and checklists and have not been duplicated as part of the TIDieR checklist. When a **randomised trial** is being reported, the TIDieR checklist should be used in conjunction with the CONSORT statement (see www.consort-statement.org) as an extension of **Item 5 of the CONSORT 2010 Statement.** When a **clinical trial protocol** is being reported, the TIDieR checklist should be used in conjunction with the SPIRIT statement as an extension of **Item 11 of the SPIRIT 2013 Statement** (see www.spirit-statement.org). For alternate study designs, TIDieR can be used in conjunction with the appropriate checklist for that study design (see www.equator-network.org).

For peer review only - http://bmjopen.bmj.com/site/about/guidelines.xhtml
